# The origins of phagocytosis and eukaryogenesis

**DOI:** 10.1186/1745-6150-4-9

**Published:** 2009-02-26

**Authors:** Natalya Yutin, Maxim Y Wolf, Yuri I Wolf, Eugene V Koonin

**Affiliations:** 1 National Center for Biotechnology Information, National Library of Medicine, National Institutes of Health, Bethesda, MD 20894, USA

## Abstract

**Background:**

Phagocytosis, that is, engulfment of large particles by eukaryotic cells, is found in diverse organisms and is often thought to be central to the very origin of the eukaryotic cell, in particular, for the acquisition of bacterial endosymbionts including the ancestor of the mitochondrion.

**Results:**

Comparisons of the sets of proteins implicated in phagocytosis in different eukaryotes reveal extreme diversity, with very few highly conserved components that typically do not possess readily identifiable prokaryotic homologs. Nevertheless, phylogenetic analysis of those proteins for which such homologs do exist yields clues to the possible origin of phagocytosis. The central finding is that a subset of archaea encode actins that are not only monophyletic with eukaryotic actins but also share unique structural features with actin-related proteins (Arp) 2 and 3. All phagocytic processes are strictly dependent on remodeling of the actin cytoskeleton and the formation of branched filaments for which Arp2/3 are responsible. The presence of common structural features in Arp2/3 and the archaeal actins suggests that the common ancestors of the archaeal and eukaryotic actins were capable of forming branched filaments, like modern Arp2/3. The Rho family GTPases that are ubiquitous regulators of phagocytosis in eukaryotes appear to be of bacterial origin, so assuming that the host of the mitochondrial endosymbiont was an archaeon, the genes for these GTPases come via horizontal gene transfer from the endosymbiont or in an earlier event.

**Conclusion:**

The present findings suggest a hypothetical scenario of eukaryogenesis under which the archaeal ancestor of eukaryotes had no cell wall (like modern *Thermoplasma*) but had an actin-based cytoskeleton including branched actin filaments that allowed this organism to produce actin-supported membrane protrusions. These protrusions would facilitate accidental, occasional engulfment of bacteria, one of which eventually became the mitochondrion. The acquisition of the endosymbiont triggered eukaryogenesis, in particular, the emergence of the endomembrane system that eventually led to the evolution of modern-type phagocytosis, independently in several eukaryotic lineages.

**Reviewers:**

This article was reviewed by Simonetta Gribaldo, Gaspar Jekely, and Pierre Pontarotti. For the full reviews, please go to the Reviewers' Reports section.

## Background

It is universally accepted that mitochondria and related organelles, that so far have been discovered in all eukaryotes studied in sufficient detail, have evolved via endosymbiosis, most likely, a single endosymbiotic event that involved an alpha-proteobacterium, the apparent ancestor of the mitochondria [1-4]. However, the place of the mitochondrial endosymbiosis in the course of eukaryogenesis and the nature of the host of the alpha-proteobacterial endosymbiont remain hotly debated matters [1,5,6]. Under the so-called archezoan hypothesis, the organism that acquired the endosymbiont was a proto-eukaryote (dubbed the archezoan) that already possessed the nucleus, the endomembrane system, the cytoskeleton, and other hallmark structures of the eukaryotic cell [5,7,8]. In other words, the hypothetical archezoan is envisaged as an amitochondrial, unicellular eukaryotic organism. The major difficulty faced by the archezoan hypothesis is that so far all candidate archezoa, such as *Diplomonada*, *Parabasalia*, and *Microsporidia*, have been shown to possess organelles derived from or, at least, related to mitochondria (hydrogenosomes, mitosomes, and others) as well as some nuclear genes of apparent mitochondrial (alpha-proteobacterial) origin [1,6]. Thus, the proponents of the archezoan hypothesis are forced to postulate that the archezoa represent an extinct lineage of primitive eukaryotes [8].

The hypotheses that oppose the archezoan concept are symbiotic scenarios in which the mitochondrial endosymbiosis is seen as the event that triggered eukaryogenesis in the first place. This idea traces back to the classic 1967 paper of Sagan (Margulis) [4] but received a major boost from the discovery of mitochondria-related organelles and genes of apparent mitochondrial origin in all thoroughly characterized eukaryotic cells [1,9,10]. Under the symbiotic scenarios that differ in details, the host that engulfed the alpha-proteobacterial ancestor of the mitochondria is posited to have been not a proto-eukaryote but rather an archaeon that closely resembled the currently known archaea, at least, in terms of the cell organization [1,11-13]. The advantage of the symbiotic scenarios is that they provide plausible, even if rather general explanations for the origin of the remarkable organizational and functional complexity of the eukaryotic cell as a result of diverse interactions between the host and the endosymbiont. However, the potentially serious difficulty faced by these scenarios is that prokaryotes have no known mechanisms for engulfing other prokaryotic cells (although at least one case of endosymbiosis among bacteria has been reported [14]). Thus, under these scenarios, the symbiosis between two prokaryotic cells would depend on an extremely rare, if not unique, spurious event – the "fateful encounter" hypothesis using the memorable phrase of De Duve [15].

By contrast, many cells in a variety of eukaryotes possess elaborate mechanisms for the internalization of bacteria and other large particles, collectively named phagocytosis [16]. In some unicellular eukaryotes, such as amoebas, phagocytosis can lead to the establishment of new endosymbiotic relationships [17]. Accordingly, adepts of the archezoan hypothesis of eukaryogenesis maintain that the amitochondrial protoeukaryotes have already evolved the phagocytic capacity [18] – the "primitive phagocyte" hypothesis according to De Duve [15]. This cellular function would provide the protoeukaryotes with the possibility of numerous trials and errors in their relationship with bacteria, so that one of these trials would end up in the acquisition of the proto-mitochondrion that would allow the mitochondrial eukaryotes to outcompete the primitive, amitochondrial forms (which is why, under this scenario, the latter are no longer around) [19].

Thus, the origin of phagocytosis appears to be one of the key aspects of eukaryogenesis, and reconstruction of the evolution of the phagocytic function could substantially inform our thinking on the origin of the eukaryotic cell and, in particular, might help to distinguish between the archezoan and symbiotic scenarios.

Phagocytosis is defined as internalization of particles larger than 0.4 μm in diameter [20]. Phagocytosis serves diverse functions, from feeding on bacteria in various unicellular eukaryotes [21,22], to apoptotic sell removal [23], tissue remodeling, and immune defense in animals [16,24-28]. Plant cells are not phagocytic owing to their rigid cell walls. However, the engulfment of rhizobia symbionts into root hairs occurs via a phagocytic mechanism [29]. Other cases of bacterial-plant symbioses involve mechanisms of entry that are apparently distinct from phagocytosis [30]. To date no phagocytosis has been reported in fungi, with the interesting exception of the basal parasitic fungus *Rozella allomycis *that was reported to phagocytose organelles of its host [31].

Phagocytosis (uptake of large particles) is a distinct form of the more general mechanism, endocytosis (uptake of extracellular substances), the other form of which is pinocytosis, the uptake of solutes and small particles [32]. In addition to the classic phagocytosis, many eukaryotes are capable of macropinocytosis, a process that is characterized by the formation of large compartments of irregular size. During macropinocytosis, the ingested particle initiates ruffling of the adjacent area of the plasma membrane; the mechanism induced mainly by phosphatidylserine (PS); in contrast, "classic" phagocytosis occurs by tight flow-over of the plasma membrane on the particle surface ("zipper" model) [25]. Unlike phagocytosis, macropinocytosis yields spacious compartments in which extracellular liquid is internalized along with a bacterial or apoptotic cell. Importantly, both macropinocytosis and phagocytosis depend on the formation of membrane protrusions supported by actin filaments, whereas other types of endocytosis occur by membrane invagination.

Phagocytosis depends on the endoplasmic reticulum (ER) compartments in a complex manner. An early phagosome has been reported to fuse, consecutively, with the early/sorting/recycling endosome, late endosome and lisosome [20,33]. The ER involvement in phagocytosis was also supported by the study of the Ap1 protein that is required for endocytic vesicle formation: Ap1-mutant *Dictyostelium *cells showed defects in the phagocytosis and macropinocytosis, but not in cell adhesion [34]. However, evidence to the contrary was presented as well: the absence of visible internal vesicle fusion and the existence of a continuous layer of F-actin surrounding the phagocytotic cup have been interpreted as indications that fusion between the phagosome and ER compartments was unlikely [35].

Phagocytosis is one of the numerous and diverse actin-dependent processes that occur in all eukaryotic cells. Actin polymerization requires one or more of the following assembly factors: (i) the Arp2/3 complex that consists of 7 distinct subunits and produces branched filaments (actin network, or mesh) and is activated by either WASp/N-WASp or Scar/WAVE proteins; (ii) formins that produce linear filaments and are activated by profilin; (iii) spire that contains four WASP homology domains and also produces linear filaments [36]. Actin filaments comprise the structural basis of at least 15 distinct structures in metazoan cells including sheet-like protrusions such as lamellipodia and lamella (with the involvement of Arp2/3, formins, cortactin, ADF/cofilin, and Scar/WAVE [37]), ruffles (Arp2/3, formins, RhoA, Scar/WAVE), phagocytic cups (Arp2/3, formins, Scar/WAVE or WASp/N-WASp), finger-like protrusions such as filopodia [require formins and, possibly, Arp2/3 for the assembly of parallel filaments but do not require WASp proteins, and pits including those involved in clathrin-dependent endocytosis (require Arp 2/3, N-WASP and other proteins, and in caveolin-dependent endocytosis (caveolin, flotillin) [36]). In addition, actin polymerization is required for membrane traffic, cytokinesis, focal adhesion structures, exocytosis [38]. Despite the high variability of phagocytosis mechanisms, they share the same mechanism of actin polymerization that requires the input of the Arp2/3 complex, WASp/WAVE proteins, and Rho GTPases as key regulators [28].

Many pathogens exploit the phagocytic abilities of eukaryotic cells for the entry into the host cell. Although bacteria utilize different invasive strategies, and entry sites show different morphology, triggering actin polymerization via Rac, Cdc42, WASp or WAVE, and the Arp2/3 complex seems to be a common denominator for all cases [39,40].

Owing to the universality of molecular mechanisms of cytoskeleton-driven processes, there are notable similarities between phagocytosis and some of these other processes. The generation of the phagocytic cup has been compared mechanistically to the formation of lamellipodia and filopodia, a process that requires actin nucleation on the membrane face [37,41]. Membrane-associated signaling GTPases, Rac, Rho or cdc42, recruit and activate nucleation-promoting factors (NPF), such as the Wiskott-Aldrich syndrome protein (WASP), which in turn bind the ARP2/3 complex [42,43]. The Arp2/3 complex then nucleates the assembly of an actin filament. The critical role of this cascade has been demonstrated experimentally through the inhibition of both C3- and IgG-mediated phagocytosis by overexpression of the Arp 2/3 activating fragment of the NFP Scar1 [44]. The phagosome formation also requires cofilin which severs actin filaments and generates new barbed-ends for elongation. The two processes are thought to occur synergistically to facilitate active ruffling that occurs during the phagocytic process [45].

Chemotaxis in eukaryotes also shares common features with phagocytosis [46], namely, the central role of actin polymerization (Chen et al., 2003) that involves regulation by Rho family GTPases and Wasp activation of Arp2/3 [47] as well as regulation by phosphoinositide 3-kinase (PI3K) [48].

Phagocytosis (at least, the CR3-mediated form) has been viewed as an adapted cell-adhesion mechanism [24]. Talin, vinculin, paxillin and focal adhesion kinase (FAK) are adhesion proteins that might also be involved in phagocytosis [24]. Furthermore, phagocytosis is regulated via many of the same pathways that are involved in cell adhesion mechanisms and rely on a distinct set of regulatory proteins including Rho, Rac, Cdc42, Src-kinases, Arf6, and PI3K [49-51]. It even has been proposed that cell spreading during adhesion represents abortive phagocytosis of an infinitely large virtual particle. Indeed, recent genetic studies have confirmed that adhesion and motility were impaired in many of the same mutants of *Dictyostelium discoideum *and *Caenorhabditis elegans *that displayed defects in phagocytosis, and *vice versa *[49].

As with other organelles, proteomics of phagosomes isolated from diverse organisms provides for a detailed characterization of the protein machinery that is involved in phagocytosis. In principle, comparative proteomic approaches should allow researchers to identify phagosomal proteins that are conserved in different species and to track changes in the protein content during phagosome maturation [52]. However, the differentiation between the true phagosomal components and contaminants remains a difficult problem. Furthermore, many proteins that play important roles in phagocytosis are only transiently associated with the phagosome, and so could escape detection by proteomic methods.

With the ultimate goal of gaining insight into the origin of the eukaryotic cell and making inroads into distinguishing between the "primitive phagocyte" and "fateful encounter" models, we compared 7 available phagosome proteomes from 5 diverse eukaryotes, examined the phyletic distributions of major proteins involved in phagocytosis, and investigated the phylogenies of two key protein groups that have readily identifiable prokaryotic homologs, namely, the actin-related family and the Ras superfamily of small GTPases. We use the results of this analysis to propose a tentative scenario for the origin of phagocytosis in relation to the origin of eukaryotes.

## Results

### The core set of proteins involved in phagocytosis identified by comparison of the proteomics results

To delineate the complement of the evolutionarily conserved protein components of the phagosome, we compared the sets of proteins identified in proteomic studies of phagosomes isolated from mouse macrophages [53,54], *Drosophila *S2 cells [55], *Dictyostelium discoideum *[56], *Entamoeba histolytica *[57,58], and *Tetrahymena thermophila *[59]. Altogether, these studies yielded approximately 2000 proteins associated with the phagosomes. We first clustered these proteins into sets of likely orthologs by mapping them to eukaryotic clusters of orthologous domains (KODs; [60]). Proteins that mapped to the same KOD were considered orthologous. With this approach, over 900 sets of orthologous phagosomal proteins were identified. However, the level of conservation of phagosomal proteomes, as revealed by the comparison of the protein sets identified in the proteomic analyses, was surprisingly low: 118 proteins were detected in at least 3 of the 5 organisms for which proteomic analysis of phagosomes has been reported, of which 29 proteins were found in 4 species, and only 9 proteins were seen in all 5 species (Tables 1, 2 and 3, Additional File 1 and Additional File 2) [58,61-103]. This lack of phylogenetic coherence could be due to the imperfect detection of phagosomal proteins with the applied proteomics methods, the substantial variability of the phagocytosis machinery or, most likely, by a combination of both these factors. For instance, actin-related protein 3 (Arp3) was identified only in 3 of the 5 species, and actin-related protein 2 (Arp2) was detected in only one species (and therefore failed to make the list of phagosomal proteins that was automatically compiled for the purpose of the present study). As there is no reasonable doubt that the Arp2/3 complex is engaged in phagocytosis in all phagocytic organisms, these discrepancies point to the limitations of the proteomic analysis. Conversely, even interstrain differences in the phagosome composition, apparently, can be substantial. For instance, proteomic analysis of phagosomes isolated from the laboratory strain of *Entamoeba histolytica *and two clinical isolates of the same species yielded only ~20% of common proteins in all three strains; given that the proteomic analysis was performed by the same method and on essentially identical amoeba cultures, it appears extremely unlikely that most of these differences are proteomic artifacts [57].

**Table 1:** Key receptor proteins involved in phagocytosis^a^

**Protein family; a representative (GI)**	**Detection in proteomic studies (number of species, out of 5 studied)**	**Role in phagocytosis**	**Domain architecture (with CDD IDs)^**b **^and structural features (N-C)**^**c**^	**Range of orthologs in eukaryotes**	**Prokaryotic homologs**	**References**
Fcγ 204121	1 (mouse)	Fcγ binds IgG- opsonized particles and initiates assembly of protein complexes inside the macrophage	Two tandem immunoglobulin domains (cl00093); one membrane-spanning domain	This domain architecture is present only in Mammalia; immunoglobulin domains are found in all animals	Only distantly related immunoglobulin domains in some bacteria	[66]

Complement receptor C3 (CR3) (α_M_β_2 _integrin)		Binds C3bi- opsonized particles, initiates intracellular signal cascade leading to particle uptake (macrophages)				[63,68]
			
α_M _integrin 88501734	2 (mouse, *Drosophila*)		Von Willebrand factor type A (vWA) domain (cd014690), two Integrin alpha (beta-propellor repeats) domains (smart00191), Integrin alpha domain (pfam08441); a transmembrane domain	All animals	No orthologs, but vWA domain is common in both archaea and bacteria; integrin alpha repeats are found in some bacteria	

β2 integrin 124056465	2 (mouse, *Drosophila*)		vWA domain (cl000057), Integrin beta tail domain (pfam07965), transmembrane region; Integrin beta cytoplasmic domain (pfamo8725)	All animals	Integrin beta chain-like proteins are present in cyanobacteria (e.g., 113475558); vWA domains are present in many archaea and bacteria.	

epidermal growth factor (EGF) receptor 6478868	0	growth factors enhance macropinocytosis in some mammalian cell lines	A tandem repeat of a unit containing Receptor l domain (pfam01030) and Furin-like cysteine rich region (pfam00757); a transmembrane region; cytoplasmic Protein Tyrosine Kinase domain (cd001920).	All animals	No orthologs but Protein Tyrosine Kinase domain is common in archaea and bacteria.	[61,62,64]

Mannose receptor 109895388	1 (mouse)	Mannose receptor is the main phagocytic receptor of human alveolar macrophages that perform opsonin-independent phagocytosis. Mannose receptor is required for phagocytosis in dinoflagellates (*Oxyrrhis marina*), *T. vaginalis*, and microglia	Carbohydrate-binding domain RICIN (cd00161); Fibronectin Type II domain (cd00062); eight C-type lectin-like domains (CLECT; cd00037); a transmembrane domain	This domain architecture is conserved inl *Chordata*. Other animals, plants, *Choanoflagellida*, *Kinetoplastida*, and green algae have various arrangements of CLECT domains, from single domain to tandem arrays of 11 domains (*S. purpuratus*).	No orthologs; CLECT domain is also present in some bacteria	[22,65,67,69,70]

CED-1/MEGF-10/Draper/LRP1 12597465	0	The main receptor in apoptotic cell (AC) phagocytosis	EMI domain, a cysteine-rich domain of EMILINs and other extracellular proteins; multiple EGF-like motifs (cl02497); a transmembrane domain	All animals. Although putative homolog of MEGF10/CED-1 is present in Entamoeba [148], our screening did not reveal it, perhaps due to lack of sequence similarity.	None	[67]

SibA-SibD of *Dictyostelium* 60465670	0	Adhesion receptor with structural and functional similarities to metazoan integrin beta chains.	vWA domain; four bacterial-like repeats (RTX family/adhesion- like protein); a transmembrane domain	*Dictyostelium *only	Partial: RTX family protein, adhesin like protein in various bacteria	[75]

Transmembrane 9 (TM9) proteins/Phg1 74859302	1 (*Drosophila*)	Membrane proteins essential for cellular adhesion and phagocytosis	Endomembrane protein 70 (pfam02990): N-terminal signal peptide followed by a large extracellular domain and 9 transmembrane domains	All except for *Giardia *and *Entamoeba*	None	[71,72,76]

SadA 60465074	0	Involved in phagocytosis of *Dictyostelium*, possibly, as an adhesion receptor.	Three EGF-like repeats (pfam07974); nine transmembrane domains	*Dictyostelium *only; EGF repeats of SadA are similar to corresponding regions of tenascins and integrins.	None	[77]

^a^Only proteins whose role in phagocytosis was characterized in detail are included.

^b^The domain identifier from the Conserved Domain Database [96] is given in parentheses.

^c^The domains are listed from the N-terminus to the C-terminus of the respective protein.

**Table 2:** The actin-centered core of the phagocytosis machinery: components of actin filaments and proteins involved in filament remodeling^a^

**Protein family; a representative (GI)**	**Detection in proteomic studies (number of species, out of 5 studied)**	**Role in phagocytosis**	**Domain architecture (with CDD IDs)^**b **^and structural features (N-C)**^**c**^	**Range of orthologs in eukaryotes**	**Prokaryotic homologs**	**References**
Actin 66826069	4 (except for *Tetrahymena*)	Actin filament rearrangement is the universal mechanical basis for phagosome formation	Actin (cd00012)	All eukaryotes	A group of Crenarchaeal-Korarchaeal proteins are the closest prokaryotic orthologs of actins; MreB, FtsA, ParM, MamK and their euryarchaeal homologs (Ta0583) are distant prokaryotic homologs (see main text for details).	[24,74] [113]

*Actin-related protein 2/3 (ARP2/3) complex – 7 subunits*						

Actin-related protein 2 (ARP2) 4093161	1(*Entamoeba*)	Enables branching of actin filaments by providing new nucleation sites for G-actin. Arp2/3 complex is essential for apparently all known types of phagocytosis (not documented in *Ciliata*)	Actin (cd00012)	All eukaryotes except for *Diplomonadida*	Same as for actins	[44,118]
				
Actin-related protein 3 (ARP3) 60467470	3 (except for *Tetrahymena *and *Entamoeba*)		Actin (cd00012)	All eukaryotes except for *Diplomonadida*		

ARP complex(ARPC) protein 1 (p41-Arc) 66816255	2 (*Drosophila *and *Entamoeba*)		WD40 domain (cl02567)	All eukaryotes except for *Diplomonadida and Chlorophyta*	No orthologs but proteins containing WD40 domains are common in bacteria, particularly, those with complex signal transduction systems.	
			
ARPC2 (p34-Arc) 60467975	0		p34-Arc (pfam04045)	All eukaryotes except for *Diplomonadida *and *Apicomplexa*	none	
			
ARPC3 (p21-Arc) 50344884	1(*Entamoeba*)		p21-Arc (pfam04062)	All eukaryotes except for *Diplomonadida and Apicomplexa*	none	
			
ARPC4 (p20-Arc) 115495705	2 (mouse and *Entamoeba*)		ARPC4 (pfam05856)	All eukaryotes except for *Diplomonadida*	None	
			
ARPC5 (p16-Arc) 66806101	0		p16-Arc (pfam04699)	absent in *Diplomonadida, Apicomplexa, Chlorophyta, Choanoflagellida*, and *Ciliophora*	None	

WASp (Wiskott-Aldrich syndrome protein) 10880935	0 *Entamoeba *proteome contained RickA protein, acting as WASp (167383163)	Actin assembly factor, activates the Arp2/3 complex	WH1-irregular superhelix (proline-rich; cd01205) WH1 is a derived plekstrin homology (PH) domain Cdc42/Rac interactive binding (CRIB) motif (cd00132)	All animals and fungi, *Monosiga*, *Dictyostelium*, *Entamoeba*, ciliates, *Trichomonas*; missing in plants	None PH-fold is, largely, specific to eukaryotes. Some pathogenic bacteria have proteins that mimics WASp: RickA (168824102, *Rickettsia raoultii*), ActA (58500210, *Listeria seeligeri*).	[73,78,79,119]

WAVE1/SCAR1 66809177	1 (*Drosophila*)	Actin assembly factor, activates the Arp2/3 complex	WH1-unstructured (proline-rich) Paralog of WASp, diverged variant of WH1 domain	All animals and plants, *Dictyostelium*, *Monosiga*, *Trichomonas*	None	[83,87,89]

Profilin 730406	3 (except for *Tetrahymena *and *Dictyostelium*)	Regulator of actin polymerization, stoichiometric complex with actin, activator of WASP and SCAR (binds to their prolin-rich regions), lipid-binding	Profilin domain (cd00148)	All eukaryotes with the apparent exception of *Diplomonadida*	No orthologs. Profilin-like fold found in PAS and GAF signaling domains that are common in bacteria, and Roadblock/LC7 domain that is present in bacteria and some archaea, and interacts with small GTPases	[84-86,88]

Formin 158518557	3 (except for *Tetrahymena *and *Dictyostelium*)	nucleates the formation of linear actin filaments	Globular -unstructured proline-rich -FH2 (formin homology)	Multiple paralogs, apparently, in all eukaryotes except for *Diplomonadida*	None FH2 is a unique fold restricted to eukaryotes	[36,82]

Cofilin/ADF 3182971	4 (except for *Tetrahymena*)	reversibly controls actin polymerization and depolymerization	Cofilin domain cd00013, ADF, Actin depolymerisation factor/cofilin -like domains	Multiple paralogs, apparently, in all eukaryotes except for *Diplomonadida*	A homolog only in *Streptomyces *(112791749), probable HGT	[80]

Coronin 11023	2 (mouse and *Dictyostelium*)	associates with the Arp2p/Arp3p complex to regulate its activity	WD40 (cl02567)	Apparently, single orthologs in all eukaryotes except for *Diplomonadida*, green algae, plants	No orthologs but numerous WD40-domain proteins are present, primarily, in bacteria with complex signal transduction systems	[21,81,90]

alpha-actinin 60474969	3 (except for *Tetrahymena *and *Dictyostelium*). An actin cross-linker cortexillin (ctxA, 66804885) is found on *Dictyostelium *phagosome.	cross-links actin filaments; involved in membrane anchoring actin filaments.	CH-SPEC-EF-hands (actin-binding domain composed of two calponin homology (CH) domains (cd00014); spectrin repeats (SPEC, cd00176); EF-hand, calcium-binding domain (cd00051)	Animals, fungi, amoebozoa, *Monosiga, Trichomonas*, oomycetes; in plants, green alga, *Ciliata*, *Apicomplexa, Kinetoplastida, Diplomonada *have other EF-hand family proteins	No orthologs or homologs with high similarity but many bacteria encode EF-hand-containing proteins (e.g., 148256632)	[91,100,103]

Filamin/ABP120 121115	3 (except for *Tetrahymena *and *Dictyostelium*). An actin cross-linker cortexillin (ctxA, 66804885) is found on *Dictyostelium *phagosome.	Cross-links actin filaments	Actin-binding domain composed of two CH domains (cd00014); Filamin/ABP280 repeats (cl02665)	Animals and amoebozoa; others eukaryotes have non-orthologous EF-hand family proteins; plants have multiple CH domain proteins	None	[99,103]

^a^Only proteins whose role in phagocytosis was characterized in detail are included.

^b^The domain identifier from the Conserved Domain Database [96] is given in parentheses.

^c^The domains are listed from the N-terminus to the C-terminus of the respective protein.

**Table 3:** Regulatory Rho-family small GTPases involved in phagocytosis^a^

**Protein family; a representative (GI)**	**Detection in proteomic studies (number of species, out of 5 studied)**	**Role in phagocytosis**	**Domain architecture (with CDD IDs)^**b **^and structural features (N-C)**^**c**^	**Range of orthologs in eukaryotes**	**Prokaryotic homologs**	**References**
Cdc42 45384262	1 (mouse)	Regulates actin remodeling in Fc-mediated and AC-mediated phagocytosis, and micropinocytosis but not CR-mediated phagocytosis	Rho (Ras homology) family of Ras-like GTPases: Cdc42 subfamily (cd01874)	Animals, fungi, and *Choanoflagellida*	Orthologous relationships are hard to determine but numerous bacteria and some archaea encode diverse small GTPases	[94,121,92,25] [93] [101] CED-10 [95]
				
Rac/RhoG/CED-10 2702398	4 (except for *Tetrahymena*)		Rho family of Ras-like GTPases (cd00157)	All eukaryotes except for *Apicomplexa *and *Kinetoplastida*		
		
RhoA 2225894	2 (mouse and *Drosophila*)	Regulates actin remodeling in CR-mediated phagocytosis; not required for or inhibits other types of phagocytosis	Ras-like GTPases: RhoA-like subfamily (cd01870)	Animals, fungi, and *Choanoflagellida*		[97,98,102]

^a^Only proteins whose role in phagocytosis was characterized in detail are included.

^b^The domain identifier from the Conserved Domain Database [96] is given in parentheses.

^c^The domains are listed from the N-terminus to the C-terminus of the respective protein.

As phagocytosis depends on cytoskeleton remodeling, it was not unexpected to find actin, tubulin, non-muscle myosin, and several actin-binding proteins among the conserved phagosomal proteins. In addition, several signaling GTPases and their effectors are conserved as well. The small GTPases are mostly represented by the Rab family members that are involved in vesicular fusion and trafficking. Because these proteins are associated with endosomes, it is difficult to distinguish whether they regulate phagocytosis itself or are recruited to the phagosome membrane as the result of phagosomal-endosomal fusion. The presence of several lysosomal proteins in the conserved phagosomal set is likely to reflect the fusion of the phagosome with the lysosome which contributes both its membrane (hence the presence of V-ATPase) and its compartment (hence hydrolytic enzymes) to the maturing phagosome. Surprisingly, one soluble, cytosolic hydrolase (S-adenosylhomocysteine hydrolase) was also identified. Several proteins characteristic of the endoplasmic reticulum (ER) were detected; this observation reflects the apparent role of ER in phagosome formation and/or maturation. Components of the TCP1-ring chaperonin complex that mediates the folding of various cytoplasmic proteins including actin and tubulin, as well as the HSP70 and HSP90 chaperones (heat shock proteins), whose role in endocytosis has been demonstrated, also belonged to the conserved phagosomal protein set. In addition, and unexpectedly, this set included numerous ribosomal proteins, translation elongation factors, and several mitochondrial and metabolic proteins.

Obviously, the collections of phagosomal proteins identified by proteomic approaches include numerous false positives and false negatives. The false-positive group seems to consist, primarily, of some of the abundant cytosolic and organellar proteins that contaminate the isolated phagosomes. The false-negative group would include proteins that, although functionally important, only transiently associate with the phagosome, and therefore, escape detection by proteomics. Accordingly, we attempted to correct and extend the list of phagosomal proteins by including proteins that have not been consistently detected by proteomics but whose role in phagocytosis has been convincingly demonstrated in at least two organisms, and excluding proteins without concrete functional roles in phagocytosis.

### The major proteins involved in phagocytosis and their phyletic distribution: the conserved actin-centered core of phagocytosis

The phyletic distribution of proteins whose role in phagocytosis has been documented experimentally (and, in many cases, supported by proteomic analysis) was characterized by sequence similarity search (Tables 1, 2, 3, Additional File 3 and Additional File 4). These proteins fall into several distinct functional categories among which a gradient of evolutionary conservation is seen from the "periphery" to the "center" of the phagocytosis machinery (Tables 1, 2, 3, Additional File 3 and Additional File 4). Despite the often propounded view that phagocytosis is an evolutionarily conserved mechanism [104], molecular details of this process are remarkably variable between species and different types of phagocytosis. Receptors are a poorly conserved part of the phagocytic machinery: indeed, no universal phagocytic receptors have been identified (Table 1). The key regulators including actin-remodeling proteins (Table 2), and small GTPases of the Rho family (Table 3) are conserved to a greater extent although non-orthologous displacement is observed, e.g., RhoA and Cdc42 appear to be mutually exclusive as regulators of different types of phagocytosis (see also Additional File 3).

Actin, the actin nucleation complex Arp2/3 and its regulators WASP/N-WASP or WAVE/SCAR, and actin-binding proteins that are involved in remodeling of actin filaments, such as gelsolin, profilin, cofilin, formin, and coronin, comprise the structural core of phagocytosis that is conserved in (nearly) all eukaryotes (Table 2). The (near) universality of these components is manifest both in their presence in all phagocytic organisms and in their involvement in all types of phagocytosis. The actin-remodeling machinery involved in phagocytosis is the same as that functioning in other cytoskeleton-driven processes such as cell motility, adhesion, and clathrin-mediated endocytosis. Thus, the results of the phylogenomic analysis of phagocytosis suggest that the actin-centered core of the phagocytic machinery that is represented in (nearly) all eukaryotes was present in the Last Eukaryotic Common Ancestor (LECA) in, essentially, the modern form. By contrast, the rest of the phagosome components cannot be mapped to LECA.

Thus, understanding the origin of phagocytosis and the other processes that involve actin remodeling requires the elucidation of the evolutionary histories of this set of actin-associated proteins, as well as the key regulators of phagocytosis. In particular, the prokaryotic connections and likely prokaryotic roots of these proteins are of critical importance. The majority of the proteins involved in phagocytosis have no prokaryotic homologs or have only distant connections (such as a common structural fold) at the level of individual domains that are, clearly, not informative in phylogenetic analysis (Tables 1, 2, 3). In particular, despite an exhaustive search, no prokaryotic homologs were detected for 4 of the 5 accessory subunits of the ARP2/3 complex that are highly conserved in all eukaryotes. The major exceptions are the proteins of the actin family and the small regulatory GTPases of the Rho family as well as protein kinases. The first two groups of proteins are essential for all forms of phagocytosis and other cytoskeleton-related processes, so we investigated their likely origins in detail.

### The archaeal ancestry of actins

Bacteria and archaea encode well-characterized proteins homologous to actin such as MreB, FtsA, ParM, and MamK [105-107]. MreB is essential for rod-shape maintenance and chromosome partitioning of bacteria, FtsA interacts with the tubulin-like FtsZ ring and is involved in cell division, whereas ParM (StbA) is a plasmid-encoded protein required for segregation. The MreB and ParM proteins are structurally similar to actin and polymerize to form filaments [108,109]. The MamK protein is essential for the spatial organization of magnetosomes and also forms filamentous structures [110].

The sequence similarity between actins and their closest bacterial homologs, MreB and FtsA, is extremely low despite the conservation of the main structural elements of the RNAseH/HSP70 fold and the amino acid residues that are essential for the ATPase activity [105]. This limited sequence conservation parallels the case of tubulin that is weakly similar to the bacterial/archaeal homolog, FtsZ, and sharply contrasts the high level of conservation between eukaryotic and bacterial Hsp70 chaperones, proteins that possess the same core fold as actins. Considering the central importance of the cytoskeleton for the very existence of eukaryotic cells, deciphering the underlying causes of the dramatic sequence divergence between actin (and tubulin) is central to the understanding of eukaryotic origins. At least, two distinct although not necessarily incompatible hypotheses can be considered.

1. The low sequence conservation, largely, reflects the fact that MreB and actin (as well as FtsZ and tubulin) diverged from their common ancestor at a very early stage of evolution, prior to the divergence of eukaryotes and bacteria from their last common ancestor [106,111]. By contrast, under this scenario, Hsp70, like many eukaryotic proteins of bacterial origin, was acquired subsequent to the mitochondrial endosymbiosis.

2. The low sequence conservation is a consequence of a dramatic acceleration of evolution of actin that was triggered by the major functional switch associated with the origin of the eukaryotic cytoskeleton.

Unlike the well-characterized bacterial homologs of actin, archaeal actin-like proteins have not been studied in detail. The only archaeal actin homolog for which both the crystal structure [112] and a biochemical characterization [113] have been reported is the Ta0583 protein from the euryarchaeon *Thermoplasma acidophilum*. Although this protein shows the greatest sequence similarity to ParM and MreB of various bacteria, it has been noticed that the biochemical properties of this protein resemble those of eukaryotic actins. In particular, the *T. acidophilum *protein formed helical filaments like actins, in contrast to MreB that polymerizes into sheet-shaped structures [113].

Crenarchaeota of the order *Thermoproteales *and the only sequenced genome of *Korarchaeum *[114] encode functionally uncharacterized actin homologs that are significantly more similar to eukaryotic actins than to bacterial or euryarchaeal MreB-like proteins (for instance, the actin-like protein of *Candidatus Korarchaeum cryptophilum *showed a highly significant similarity, with e-values below 10^-8^, to a variety of eukaryotic actins, whereas the similarity to bacterial homologs was not statistically significant). Thus, these crenarchaeal and korarchaeal proteins could be classified as archaeal actin-like proteins rather than MreB-like proteins. The presence of actin-like proteins in a single branch of Crenarchaeota and in the distantly related *Korarchaeum *suggests that the functional change associated with accelerated evolution that led to the emergence of the actin family occurred in one of those of these groups, and the actin-like protein gene was horizontally transferred to the other group. An alternative evolutionary scenario that would involve multiple gene losses appears less parsimonious. Of course, one has to keep in mind that the current genomic collection is unlikely to be a full representation of the actual diversity of the archaea [115], so this scenario might need revision in the future.

**Figure 1:**
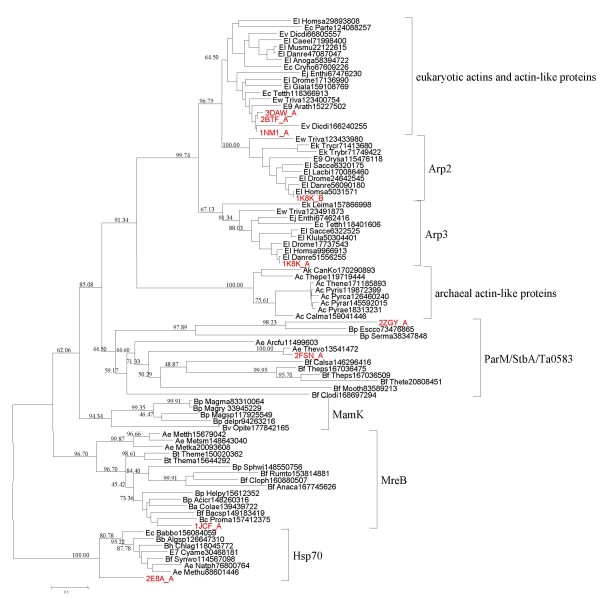
**A maximum likelihood tree of actin-related proteins**. The root position was forced between the HSP70 superfamily and the actin superfamily. The tree was constructed by analysis of 295 aligned amino acid residues (Additional File 8). Support values are indicated only for major internal branches (not within smaller monophyletic groups). The protein sequences whose structure alignment was used to correct the multiple protein alignment of actin-related proteins are denoted in red. For the complete legend, see Additional File 5.

In order to gain further insight into the relationships between eukaryotic actin protein family and their prokaryotic homologs, we reexamined the phylogeny of actins and actin-like proteins, with HSP70 family used as the outgroup. The HSP70 family is the group of proteins with the closest similarity to the actin family and thus an obvious choice for an outgroup to infer the root position in this tree although it remains uncertain whether HSP70 was present in the Last Universal Common Ancestor (LUCA) of modern cellular life or has a bacterial origin [116,117]. The resulting tree has bacterial proteins of the MreB family as the basal branch, followed by the MamK and ParM-StbA branches that also consist primarily of bacterial but also some archaeal (like the experimentally characterized Ta0583) proteins. The actin family proper that includes archaeal and eukaryotic actin-like proteins (and actins) is the sister group of the ParM-StbA group (Fig. 1). This topology seems to be best compatible with an ancient acquisition of the progenitor of the actin family by an archaeon via HGT from bacteria, perhaps, via a plasmid that encoded a ParM-StbA family protein. Regardless of exact evolutionary scenario and of which (if any) of these proteins were present in LUCA, the tree strongly supports the hypothesis that the crenarchaeal and korarchaeal actin-like proteins are the closest prokaryotic homologs of the eukaryotic actin family (Figure 1 and Additional file 5). By contrast, euryarchaeal actin-like proteins including the experimentally characterized Ta0583 clustered within the MreB and ParM branches, suggesting multiple horizontal gene transfers between bacteria and archaea. Within the eukaryotic branch of the tree, the Arp3 clade is the first to branch off the trunk, followed by the divergence of the actins and Arp2 (Figure 1).

**Figure 2:**
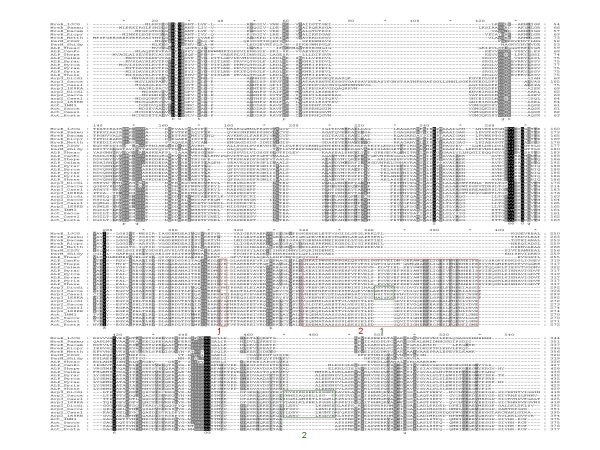
**Selected prokaryotic actin homologs aligned with eukaryotic actins and actin-related proteins 2 and 3**. Green boxes 1 and 2 highlight the major inserts in Arp 2, 3 distinguishing them from actins [118]: a loop in subdomain 4 of Arp3 (green box 1) and elongated loops in subdomain 3 in both Arp 2 and Arp 3 (green box 2). ALP, actin-like protein. Red boxes indicate homologous inserts shared between crenarchaeal and eukaryotic proteins. For the complete legend, see Additional File 5.

We then examined in detail the multiple alignment of selected representatives of the main groups of actin-superfamily proteins in search of possible derived shared characters (synapomorphies) Notably, despite the relatively low overall sequence conservation, we identified two homologous (as indicated by the amino acid residue conservation in several alignment positions) inserts that are shared between the archaeal and eukaryotic actin-like proteins; an additional conserved region that is missing in the MreB-like proteins is located near the C-termini of these proteins (Figure 2). These inserts appear to qualify as derived shared characters strongly supporting the monophyly of the archaeal and eukaryotic actins and actin-like proteins that is suggested by the topology of the phylogenetic tree (Figure 1). In addition, both archaeal actins and Arp3 possess unique inserts that are missing in eukaryotic actins (Figure 2).

The topology of the phylogenetic tree in Figure 1 and the presence of shared and similar structural features in archaeal actins could have structural and functional implications for the archaeal actin-like proteins and the ancestral forms. Structural comparison of actin, Arp2 and Arp3 shows that actin is a very compact molecule whereas Arp2 and, especially, Arp3 possess various extended loops between the core structural elements ([118] and Figure 2). The archaeal actin-like proteins have, approximately, the same overall lengths as Arp3, with some shared and some unique inserts (Figure 2). The topology of the tree in Figure 1 seems to determine the vector of evolution: the ancestral state was a molecule with extended loops, resembling Arp3, and the subsequent evolution involved loss of inserts resulting in tightening of the actin fold. Actin efficiently polymerizes from both ends to form bidirectionally growing filaments. In Arp2 and, especially, Arp3, the extended surface loops, in particular, the loop containing Insert 2, partially obstruct polymerization, producing filaments that grow unidirectionally from the so-called barbed end of the Arp3 molecule [118,119]. In extant eukaryotes, the major function of the Arp2/3 complex is the production of branched actin filament networks. This process occurs through lateral binding of the Arp2/3 complex to preexisting actin filaments, which is facilitated by WASp/Scar proteins, and the nucleation of the unidirectional elongation of a branch where Arp2 and Arp3 form the first two subunits [118-120]. Obviously, this complex system that, in part, is based on the structural and functional differentiation of actin from the Arps, is missing in extant archaea and could not have been present in the putative ancestral organism that encoded the common ancestor of actins and Arps (Figure 1). The apparent structural similarity between the archaeal actins and Arp2/3 (Figure 2) suggests that the polymerization capacity of the ancestral actin-like protein could have been similar to that of Arp3 [118-120]. Thus, it is conceivable that the relatively inefficient formation of filament networks is the ancestral functional modality of actin-like proteins.

### The bacterial roots of eukaryotic small GTPases

**Figure 3:**
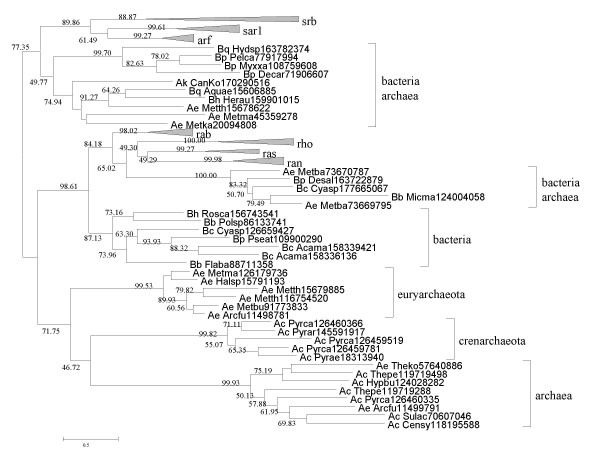
**A Maximum Likelihood tree of the Ras superfamily of GTPases**. The tree was constructed using 120 aligned positions (additional File 7). The tree is unrooted but shown in a pseudo-rooted form solely for convenience. Bacterial and archaeal clusters are shown in green and red, respectively. Support values are shown only for major internal branches. For the complete legend, see Additional File 5.

At least three members of the Rho-family of small GTPases (RhoA, Rac, Cdc42) are important regulators of actin polymerization that is required, among other cytoskeleton-related processes, for phagocytosis (Table 3). The Rho family is one of the 7 eukaryotic GTPase families that collectively comprise the Ras superfamily [121,122]. Bacterial small GTPases of the MglA family appeared to be the closest prokaryotic relatives of the Ras GTPases [121] and were used to root the Ras phylogenetic tree [122] prior to the recent discovery of a set of previously unknown, closer prokaryotic Ras homologs [123]. The inclusion of these new proteins in the phylogenetic analysis affected the topology of the Ras superfamily tree, raising the question of two independent prokaryotic origins of the eukaryotic Ras GTPases [123]. We performed a comprehensive search for prokaryotic Ras homologs by running position-specific scoring matrices (PSSMs) constructed from multiple alignments of each of the 7 families of eukaryotic Ras GTPases [123] against archaeal and bacterial sequences in the RefSeq database using PSI-BLAST [124]. By combining sequence similarity and phylogenetic analysis, we identified 67 (predicted) GTPases (61 bacterial and 6 archaeal; some of these are larger proteins containing GTPase domain and leucine-rich repeats) that appeared to be the closest relatives of the Rab, Ran, Ras, and Rho families (Additional files 6 and 7 and Figure 3). Considering the paucity of these GTPases in archaea, which sharply contrasts their wide spread among diverse bacteria, and the fact that the eukaryotic small GTPases are embedded within the bacterial part of the tree (Figure 3), it appears most likely that the common ancestor of the Rab, Ran, Ras, and Rho families was of bacterial origin and was acquired by some archaea and by the ancestors of eukaryotes via HGT, in the latter case, most likely, as a result of endosymbiosis.

## Discussion

Comparisons of the complements of proteins that are associated with phagosomes or otherwise implicated in phagocytosis in different eukaryotes show a high level of diversity, with very few components being conserved throughout the eukaryotic domain of life. This lack of evolutionary conservation sharply contrasts the results reported in similar comparative analyses of other signature cellular systems of eukaryotic cells, such as the nuclear pore [125,126] or the spliceosome [127], and suggests late, multiple origins for the full-fledged phagocytosis systems. Very few proteins involved in phagocytosis possess readily identifiable prokaryotic homologs with sequence conservation sufficient for phylogenetic analysis (Tables 1, 2, 3), and those that do are not specific to phagocytosis, but rather, are generic cytoskeleton components and regulators. Nevertheless, phylogenetic analysis of these conserved proteins leads to intriguing conclusions.

The central finding described here is that some of the Crenarchaeota and Korarchaeota possess actin-like proteins that are not only monophyletic with the eukaryotic actin family but also are structurally similar to Arp2/3 in that they contain multiple, extended inserts between core structural elements that are missing in eukaryotic actins. In Arp2 and, especially, Arp3, these inserts partially obstruct polymerization and prevent rapid, bidirectional elongation of filaments that is characteristic of eukaryotic actins. Considering these observations and the tree topology of the actin superfamily (Figure 1), we suggest that the ancestral actin-like proteins' capacity for filament formation was similar to that of Arp3, that is, these proteins formed networks of branched filaments, probably, with a relatively low efficiency.

As shown by comparative-genomic and phylogenetic analysis, the Rho family GTPases that are ubiquitous regulators of phagocytosis in eukaryotes appear to be of bacterial origin, an observation that is compatible with either of the two alternative scenarios: (i) there was no Rho-regulated phagocytosis before the engulfment of the future mitochondrial endosymbiont, and (ii) a small GTPase(s) was acquired by the future host of the protomitochondrion in a separate, earlier HGT event.

**Figure 4:**
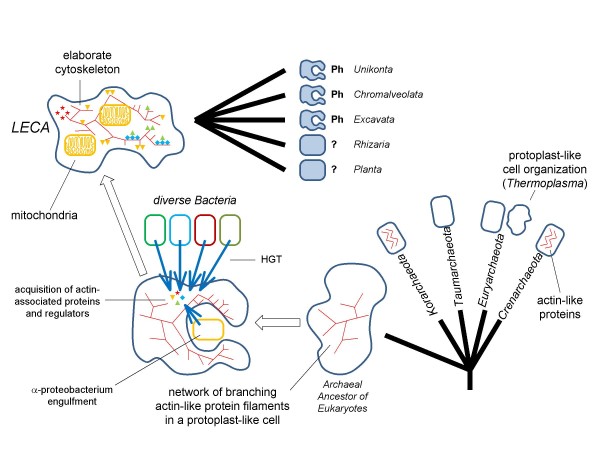
**The proposed endosymbiotic scenario of eukaryogenesis and subsequent origin of phagocytosis**. The evolutionary tree of archaea is shown as a multifurcation of 5 major branches: Crenarchaeota, Euryarchaeota, Korarchaeota, Thaumarchaeota, and the hypothetical Archaeal Ancestor of Eukaryotes which is depicted as an irregular shape to emphasize the likely absence of a rigid cell wall. LECA, Last Universal Eukaryotic Ancestor. HGT, Horizontal Gene Transfer. The primary radiation of eukaryotes is shown as a multifurcation of 5 supergroups: Unikonts, Chromalveolata, Excavates, Rhizaria, and Planta. At least, three of the supergroups evolved full-fledged phagocytosis (Ph).

Together, these findings lead to an admittedly speculative scenario for the origin and role of the phagocytic capacity in the general context of eukaryogenesis (Figure 4). Under this model, the organism that acquired the mitochondrial endosymbiont was an archaeon without a cell wall that morphologically might resemble the extant *Thermoplasma *and possessed an actin-like-protein based cytoskeleton in the form of filament networks. The branched-filament cytoskeleton allowed the hypothetical archaeal ancestor of eukaryotes to produce actin-supported membrane protrusions, resembling eukaryotic lamellipodia/filopodia, thus facilitating occasional engulfment of bacteria. One of such occasions would eventually lead to the mitochondrial endosymbiosis. Conceptually, this process can be regarded as the simplest, primordial form of phagocytosis. Indeed, it has been shown that some bacteria enter the host eukaryotic cell by inducing protrusions of the host plasma membrane [128]. This model, essentially, takes the middle ground between the "primitive phagocyte" and "fateful encounter" scenarios of endosymbiosis: it is proposed that the archaeal host had no full-fledged phagocytosis but did possess a primitive mechanism for the engulfment of other prokaryotic cells. Such an ability would substantially increase the frequency of engulfment and the likelihood that a stable enosymbiosis would be established. Thus, this model is generally compatible with the hypotheses which portray the protoeukaryote as a predator or scavenger that fed on other prokaryotes [18,104,129]; however, compared, say, to modern amoebas, this would be a rather ineffective predator. Clearly, this scenario is also compatible with the earlier "you are what you eat" idea of Doolittle according to which the protoeukaryote continuously acquired diverse genes from bacteria engulfed as food or transient endosymbionts [19].

A specific relationship between eukaryotes and *Thermoplasma *has been suggested on the basis of the results of supertree analysis [130] but a recent comprehensive phylogenetic study suggests that the archaeal "parent" of eukaryotes, most likely, belonged to a deep branch of archaea that lies outside the currently known archaeal diversity [60]. The observed distribution of actin homologs is compatible with this view in that the eukaryotic-type actin-like proteins are not seen in Euryarchaeota but are present in two other distinct, major branches of archaea, namely, a subset of Crenarchaeota and Korarchaeota. Thus, we hypothesize that the host cell that engulfed the future mitochondrion was a mesophilic archaeon that belonged to a still unknown, deep archaeal branch and possessed an actin-like protein capable of forming networks of filaments. The alternative assignment of the archaeal root of eukaryotes to Crenarchaeota (eocytes) that is suggested by some phylogenetic analyses [131-133] would imply that the actin-like proteins emerged within this archaeal phylum and that the archaeal parent of eukaryotes was a mesophilic crenarchaeon As seen with modern mesophilic archaea, such as *Methanosarcina *[134,135], an archaeon with such a life style (regardless of its exact phylogenetic position) would likely acquire a variety of bacterial genes via HGT, even prior to the endosymbiosis, a process that might account for the diversity of genes of apparent bacterial origin seen in eukaryotes [2,136].

With the input of the horizontally transferred bacterial genes and, particularly, the endosymbiont genome, Rho-GTPases and some of the actin-interacting proteins (such as ARPC1 and coronin containing the WD40 domain or profiling containing the Rossmann-type domain that could be a highly diverged derivative of PAS or GAF) were recruited as regulators of actin assembly. Conceivably, endosymbiosis would put a high premium on the evolution of the cytoskeleton that is intimately involved, among other processes, in mitochondrial dynamics [137,138]. The modern-type phagocytosis as well as cell adhesion could evolve only after the endocytic system because endomembrane delivery is required for the formation of the phagocytic cup [25]. Furthermore, the origin of phagocytosis must have succeeded the evolution of lysosomes given that the phagosome-lysosome fusion is a crucial step of phagocytosis in all modern phagocytic eukaryotes [27]. In the course of the subsequent evolution, full-fledged phagocytosis seems to have evolved independently in, at least, three of the five supergroups of eukaryotes [139,140], namely, Unikonts (metazoa and amoebozoa), Chromalveolata (ciliates), and Excavates (trypanosomes).

## Conclusion

The protein composition of phagosomes is highly variable among eukaryotes, suggesting that the modern-type, advanced phagocytosis evolved independently and relatively late in the course of evolution of several major eukaryotic lineages. However, actin, actin-related proteins, and the core set of proteins that are involved in actin filament remodeling as well as some of the key regulatory proteins are conserved across eukaryotes. All these proteins are required for a variety of cytoskeleton-dependent process not for phagocytosis specifically. Among the key proteins involved in phagocytosis, only the actin family and the regulatory GTPases of the Ras-family have well-conserved prokaryotic orthologs. Phylogenetic analysis and structural comparison of eukaryotic actin family proteins with archaeal actin-like proteins suggest that the ancestral actin-like proteins could have been capable of the formation of branched filament structures and networks. This capacity would allow the hypothetical archaeal host of the mitochondrion to form protrusions resembling modern eukaryotic lamellipodia or filopodia and facilitating engulfment of other prokaryotes. Such engulfment of bacteria would be decisive for the acquisition of the mitochondrial endosymbiont. The Ras-family GTPases appear to be of bacterial origin and might have been recruited for the regulation of actin filament remodeling from the endosymbiont or even earlier, via horizontal gene transfer from bacteria. Thus, under the proposed model, a primitive process of particle engulfment by actin-encoding archaea might antedate eukaryogenesis whereas the full-fledged phagocytosis was a late development that occurred independently in several major branches of eukaryotes.

## Methods

### Proteomic data

The *Entamoeba histolytica *phagoproteome analyzed in this study consisted of 615 non-redundant proteins. The first set contained 440 proteins from the phagosome of the wild-type and myosin IB-overproducing strains of *E. histolytica *[58]. Proteins found only in the mutant phagosomes were omitted. The second set included 249 proteins that were identified in latex-bead containing phagosomes of three *E. histolytica *strains, HM1, KU33, and HATAJI [57]; these proteins were merged with the first set, and sequence duplicates were removed.

The phagoproteome of *Dictyostelium discoideum *consisted of 179 proteins detected in latex-bead containing phagosomes [56].

The *Tetrahymena thermophila *phagoproteome contained 87 proteins isolated from polystyrene bead-containing phagosomes of *T. thermophila*, including 73 strongly supported and 14 weakly supported phagosomal protein candidates [59].

Mouse macrophage phagoproteome consisted of 524 non-redundant proteins including 116 proteins detected in latex-bead containing phagosomes from the J774 mouse macrophage-like cell line [53] and 505 proteins detected in latex-bead containing phagosomes from the RAW 264.7 mouse macrophage-like cell line [54].

The *Drosophila *phagoproteome consisted of 617 proteins detected in latex bead-containing phagosomes of *Drosophila melanogaster *S2 cells [55].

The collected proteins were used as queries for BLAST searches [124] against subsets of the KOD database [60] containing proteomes of the same species. The respective most similar protein sequences (best hits) were assumed to be the phagosome proteins in the KOD database. Proteins that were assigned to the same KOD were inferred to be orthologous. With this approach, over 900 sets of orthologous phagosomal proteins were identified. The 118 clusters that included proteins from three or more of the five species (Additional File 1 and Additional File 2) were employed in the further analysis.

### Protein sequence analysis

Multiple alignments of the protein sequences comprising the 118 sets of proteins collected from the proteomic data were constructed using MUSCLE [141] and used to construct position-specific scoring matrices (PSSMs) [124]. The annotations of three first hits of PSI-BLAST search [124] against the RefSeq protein database using the group-specific PSSMs (see Additional File 3) were used to annotate the groups. For the list of annotated clusters, see Additional File 1. Entrez Gene [142] was used as the main source of comments added in some cases.

### Phyletic patterns

Phyletic patterns of proteins involved in phagocytosis (Tables 1, 2, 3 and Additional File 3) were derived using BLAST searches of representative proteins against the RefSeq database [143]. Hit lists were manually checked; and ambiguous hits were verified by reverse BLAST against RefSeq. The presence of a protein in phagosome proteomes was validated by BLAST searches of representative proteins against collected phagosome proteins. Hit lists for the proteins listed in Tables 1, 2, 3 can be found in Additional File 2. For the Rho-family GTPases, a PhyML tree [144] was built to resolve the subfamily (Cdc42, RhoA, Rac1) relationships.

### Phylogenetic analysis

The sequences for phylogenetic analysis were retrieved from the RefSeq database [143] and aligned using MUSCLE [141]. The alignment of the actin/Hsp70 superfamily proteins was manually edited using the structural alignment of the following representative proteins: 3DAW_A (alpha-actin), 2BTF_A (beta-actin), 1NM1_A (*Dictyostelium *actin), 1K8K_B (Arp2), 1K8K_A (Arp3), 2ZGY_A (StbA), 2FSN_A (Ta0583), 1JCF_A (MreB), and 2E8A_A (Hsp70). Poorly conserved positions, positions including gaps in more than one-third of the sequences, and short (apparently, truncated owing to misannotation) sequences were removed prior to tree computation.

Maximum Likelihood trees were constructed using the TreeFinder program [145], with the estimated site rates heterogeneity and with the WAG (Whelan and Goldman) substitution model [146]. The Expected-Likelihood Weights of 1,000 local rearrangements were used as confidence values of TreeFinder tree branches [147].

## Competing interests

The authors declare that they have no competing interests.

## Authors' contributions

NY collected the data, performed data analysis, contributed to the design of the study, and wrote the first draft of the manuscript. MYW contributed to data analysis. YIW contributed to the design of the study and data analysis. EVK initiated the project, contributed to the design of the study and data analysis, and wrote the final version of the manuscript.

## Reviewers' comments

### Reviewer's report 1: Simonetta Gribaldo, Institut Pasteur

This paper attempts to understand the origin of phagocytosis as a key to understand eukaryotic origins. Two hypotheses for eukaryotic origins are tested:

a) present-day eukaryotes derive from a symbiosis between an archaeon and a bacterium.

b) present-day eukaryotes derive from an independent evolutionary line.

The first hypothesis is often criticized because it implies phagocytosis ability in the archaeon or bacterial host lineage (depending on hypotheses), which is not observed today.

The second hypothesis is often criticized because it implies origin of phagocytosis before mitochondrial acquisition and thus the extinction of pre-mitochondrial eukaryotic lineages.

Thus, the authors decided to investigate the origin of phagocytosis by analyzing the phyletic pattern and phylogeny of proteins involved in this process.

Unfortunately, they found that, of around 2000 proteins identified by proteomic analysis in two metazoans, two amoebozoa, one alveolate, very few are overlapping. In fact, only 156 proteins were identified in the phagosome proteomes of at least 3 species (authors could you please indicate precisely which ones), and many known components of phagocytosis were not detected and many proteins not directly involved in phagocytosis were detected, and the authors conclude that many false negatives and false positives affect these proteomic analyses.

Thus, the authors move over to analyze 30 proteins known to be part of phagocytic processes. Only for a few of these are nearly universal in eukaryotes, making what the authors define as "the functional core of phagocytosis": actin, actin nucleation complex Arp2/3 and its regulators WASP/N-WASP or WAVE/SCAR, and actin binding proteins. If this phagocytic core was present in the LECA why then the authors infer an imperfect phagocytosis in the LECA? Page 11: "These proteins fall into several distinct functional categories among which a gradient of evolutionary conservation is seen from the "periphery" to the "center" of the phagocytosis machinery (Tables 1, 2, 3, Additional File 3 and Additional File 4)." Would the authors please precise this point? this is not immediately clear from table or additional files. It would be interesting at this point that the authors show or discuss more clearly the results of their phylogenomic analysis, i.e. map the presence/absence of these proteins onto the tree of eukaryotes and infer ancestrality and losses. What was the set of phagocytic proteins present in the LECA? This is essential to the following discussion on the origin of phagocytosis and the claim for an imperfect phagocytosis in the LECA and independent origins of a fully-fledged phagocytosis in different eukaryotic lineages. For example, if a phagocytic protein is present in at least a Unikont and Bikont it can be inferred to have been present in the LECA and subsequently lost in the other eukaryotic lineages rather than appeared multiples times independently. From additional figure 3 it seems that all proteins were present in the LECA and then a few were lost independently in a few eukaryotic lineages.

I think this is essential for further discussion on the phagocytic capacities of the LECA.

*Authors' response: The presence-absence of the phagosome components in various eukaryotic lineages is noted in *Tables 1, 2, 3. *An explicit phylogenomic analysis with a tree etc seems superfluous because the results are trivial, in the sense that the components of the actin-based core are highly conserved and can be confidently assigned to LECA whereas (almost) all of the rest of the phagosome proteins cannot be. We added a simple statement to that effect*.

Only a few of these 30 proteins have prokaryotic homologues, i.e. actin family and small regulatory GTPases of the Rho family. Then, the authors move over to analyze the phylogeny of these two protein families. A phylogenetic analysis of the actin family is then performed. Could the authors indicate how many unambiguously aligned positions were kept for phylogenetic analysis?

What is the rationale of rooting by a distant outgroup such as HSP70? I fear that sequence conservation is too low and does not give any further signal but increase the branch length and possibly provoke a long-branch attraction artifact. With the tree as presently rooted this way, you have bacteria polyphyletic, how do the authors explain this? Would archaea have acquired their actin-like from bacteria? If the authors conclude that archaea obtained their actin-like proeins by HGT from bacteria, then the root is clearly within bacteria and the authors can show the archaeal origin of eukaryotic actin without needing to include HSP70. One precision at this point, previously published analyses on HSP70 (Gribaldo 1999, Philippe 1999) clearly show that eukaryotic cytosolic HSP70 is not related to mitochondrial HSP70 and thus likely does not derive from them. These analyses concluded that HSP70 was probably present in the LUCA and then lost in archaea (and reacquired subsequently from bacteria), could the authors acknowledge this and comment?

Finally, why only a few support values are shown? Could the authors please indicate full species names with accession numbers, it is hard to understand the tree without going back and forth to the legend and there is enough space to do so.

*Authors' response: We agree that the discussion of this tree in the original manuscript was overly brief. It was expanded in the revision, in particular, in the legend where all the issues with notation are addressed. Rooting with HSP70 is fully justified: indeed, why not given that this is the protein family that is closest to the actin family. Whether or not HSP70 was present in LUCA is an open question as we acknowledge in the revised manuscript. The issues with the number of aligned residues, the labeling of branches etc are addressed in the revised figure legends and the alignment used for the tree construction is in *Additional File 8.

Regarding small GTPases, they conclude that these arose in bacteria and their eukaryotic counterparts are of mitochondrial origin. I have a major problem here: in the text it is said that only 6 archaeal homologues are found ("This search identified 67 (predicted) GTPases (61 bacterial and 6 archaeal"). I see much more archaeal sequences in the tree in figure 3. Here again, could the authors please specify how many positions were kept for analysis? From the tree topology I do not see how conclusions on a mitochondrial origin of these proteins are inferred. Again, is the tree rooted and if yes how was the root chosen?

*Authors' response: The tree is unrooted and shown in a pseudo-rooted form for convenience as indicated in the amended legend along with the number of residues. Regarding the count of GTPases, the original language was indeed somewhat confusing. The total number of prokaryotic members of the Rab-Ran-Ras-Rho branch is 67 but only a subset was included in the tree in *Figure 3. *This is made clear in the revision, and the complete list is given in *Additional File 6. *As for the conclusions, the one on bacterial origin of these GTPases is clear enough (both eukaryotic and archaeal members are embedded within bacterial branches, and the cluster that includes Rab-Ran-Ras-Rho GTPases is overwhelmingly bacterial). As for the mitochondrial origin, this is of course more speculative but generally is the default for eukaryotic genes of apparent bacterial origin*.

General conclusions from these results are that the archaeal ancestor of eukaryotes had no cell wall but an actin based cytoskeleton capable of an imperfect phagocytosis, which allowed it to acquire the mitochondrion and become a fully-fledged eukaryote. I am afraid that these conclusions are pushed a bit too far, and are not justified by the data presented. In order to say that the LECA had an imperfect phagocytosis, the ancestral set of proteins and their evolution along the eukaryotic tree should be presented. Lack of components can also mean loss.

*Authors' response: The conclusions on LECA are presented verbally. As noted above, a tree would be superfluous (and, of course, the deep topology of the eukaryotic tree is unknown anyway)*.

The discussion on Arp2 and 3 and imperfect polarization (page 19) needs to be circumstantiated by references.

*Authors' response: we assume that what is intended here is "polymerization". Relevant references already cited but added at this specific point again*.

"At face value, at least, this conclusion and, generally, the topology of the actin tree are incompatible with the staple of the archezoan hypothesis that the protoeukaryote already possessed an advanced cytoskeleton similar to that of modern eukaryotes and providing for the possibility of phagocytosis. " Why this? From the actin tree it seems to me that the LECA possessed already Arp2, Arp3 and actins. As mentioned already, this would be clearer from a figure with a tree of eukaryotes on which presence/absence (also of other phagocytosis proteins) is mapped. Moreover, can the authors discuss the fact that these Arp3-like proteins were lost from other archaeal lineages? Finally, I seem to understand that the authors suggest that the ancestor of modern archaea was a mesophilic Thermoplasma-like. This is a big statement that needs to be discussed further in the light of archaeal phylogeny. Moreover, Crenarchaeaota and Korarchaeota that appear to possess Arp3-like homologues are all hyperthermophiles.

*Authors response: The sentence in quotes was indeed confusing and was removed (see also response to Jekely below). We hypothesize that the archaeal ancestor of eukaryotes was indeed mesophilic and Thermoplasma-like in the sense of the absence of a rigid cell wall, but not related to Thermoplasma. Perhaps, a "big" statement but perfectly intuitive biologically. All this is clearly stated in the manuscript, no need to expand the discussion*.

Page 20: "This model, essentially, takes the middle ground between the "primitive phagocyte" and "fateful encounter" scenarios of endosymbiosis: it is proposed that the archaeal host had no full-fledged phagocytosis but did possess a primitive mechanism for the engulfment of other prokaryotic cells." Again, why would this feature have been lost in archaea? Can the authors speculate on this point?

*Authors' response: We do not really claim that this feature was lost in archaea. What we hypothesize is that the archaeal ancestor of eukaryotes that, in all likelihood, represented a deep branch possessed this capacity. This branch could be extinct or else could be still lurking in some obscure habitat, that is, of course, a very interesting issue*.

"With the input of the horizontally transferred bacterial genes and, particularly, the endosymbiont genome, Rho-GTPases and some of the actin-interacting proteins (such as ARPC1 and coronin containing the WD40 domain or profiling containing the Rossmann-type domain that could be a highly diverged derivative of PAS or GAF) were recruited as regulators of actin assembly."

How would actin assembly have been regulated before the acquisition of the mitochondrion?

*Authors' response: We do not know, of course. Possibly, not regulated. Experimental study of archaela acitn-like proteins might provide clues*.

Finally, I think that the data support equally either an extinct archaeal lineage at the origin of modern eukaryotes or an extinct phagocytic eukaryotic lineage. Importantly, in both cases all pre-mitochondrial lineages -be these archaeal or eukaryotic- would have gone extinct, which is the main argument usually raised by the proponents of symbiotic scenarios against the archezoa one. Could the authors explain why they prefer the first hypothesis?

*Authors' response: obviously, a big point, and the proposed solution necessarily remains speculative. In the context of the more general debate, the symbiotic scenarios at least offer a plausible path to the eukaryotic cell, and the analysis in this paper adds another important bit in the form of the possibility of existence of archaeal branched cytoskeleton*.

### Reviewer's report 2: Gaspar Jekely, Max Planck Institute for Developmental Biology, Tübingen

This interesting paper by Yutin et al. addresses one of the most important and hotly debated issues about the origin of eukaryotes, namely the origin of phagocytosis.

It is now clear that the last common ancestor of eukaryotes was able to perform phagocytosis, and possessed essentially all the characteristic eukaryotic organelles, including mitochondria. The debate is about whether phagotrophy or mitochondria came first. Since both evolved in the stem lineage leading to modern eukaryotes, the order of origins is problematic. Cell biological considerations rather favor a phagotrophic host for the mitochondrium, but there are also strong advocates for the alternative.

One way to test the two models would by a thorough phylogenetic analysis of the proteins involved in phagocytosis. If most of these proteins turned out to be of alpha-proteobacterial origin, this would favor the mitochondrium-first scenario. Conversely, if most proteins were similar to ones found in archaebacteria, the sister lineage to the eukaryotic host, the phagotrophy-first scenario would gain support.

Yutin et al. try to resolve the issue exactly this way. In my opinion, however, they fail to find a conclusive answer, or more precisely, the data fail to yield a clear support for either scenario. The solution they propose is somewhat arbitrary, at least if we want to conclude something strictly based on the data presented. I will explain why I think this is the case.

The presence of the closest relatives of actin and actin-like proteins in some archaebacteria (the most important finding of the paper!) is consistent with many scenarios. It is clear that these proteins, even if they formed branched filaments, don't make the archaebacteria that possess them phagotrophic, not even in the inefficient way as presented in the authors' scenario. So their scenario is based on, again, cell biological speculations, namely that the host cell had to lose its cell wall and had to evolve a primitive form of phagotrophy before the mitochondrial symbiosis. Even though I would agree with such a conclusion, based on cell biological speculation, I don't think it follows from the data. I of course agree with the authors that the alternative, the lack of close actin-homologs in archaebacteria, and their presence in alpha-proteobacteria would be a very strong argument against the phagotrophy-first scenario, but they didn't find this. The history of actin therefore does not resolve the issue.

Regarding the small GTPases, it seems to support the mitochondria-early scenario, but I am afraid that the correct interpretation of this tree is difficult, and depends on when and in which direction the putative HGT events took place. The proteins are present in both eu- and archaebacteria, and could have come both ways. It is also a formal possibility that the two major eukaryotic small GTPase groups were transferred early on into different prokaryotes. So, again, it is hard to make a strong conclusion.

However, all of the above does not mean that the authors' scenario is uninteresting and not worth pursuing!

*Authors' response: It seems that the differences between our interpretation of the results and Jekely's view is, mostly, in emphasis*. *We do not deny that that the comparative-genomic and phylogenetic analyses do not unequivocally determine the evolutionary scenarios, so "cell-biological" speculation has its own important role*. *In the case of small GTPases, the evidence of bacterial origin of the eukaryotic proteins involved in particular in the regulation of phagocytosis seems strong *(Fig. 3).

I have another problem regarding the independent origin of advanced phagocytosis in different eukaryotic groups. I think it is unjustified. First of all, there are many near-universal factors (29 proteins conserved in 4 out of 5 species), as identified by the comparative proteomic study. These proteins include the core actin nucleation, branching and polymerization machinery (Arp2/3, actin, myosin, Rabs etc.). I think this can equally be interpreted the opposite way, namely that the core machinery is remarkably conserved. The authors compare the phagosome proteome to the spliceosome and the NPC, but I don't think these are the best choice, since both are multiprotein complexes, and not membrane compartments/processes. It would be more informative to compare phagosome conservation for example to the conservation of endosome or peroxisomes proteome. By the way, the low conservation of the *Entamoeba *phagosome proteome among different strains also is contrary to the authors' argument. According to the same logic, these different *Entamoeba *strains evolved advanced phagocytosis three times independently. Adding to this, the fact that there is no conserved phagocytosis receptor only indicates that these organisms phagocytose very different things, for which different receptors are needed. All in all, the divergence of the proteomes is most likely due to evolutionary divergence and the differences in methodology. I think it is still a very safe conclusion that the eukaryotic common ancestor was an advanced phagotroph with actin-based protrusion, prey uptake and acidification by V-ATPase. If all this basic cell biology is not, then what would be considered as conserved?

*Authors' response: As emphasized in the paper, the conserved components of the phagocytic machinery comprise the generic cytoskeleton, whereas the componentry specific to phagocytosis is poorly conserved. The argument that the differences in the composition of the phagosomes in different organisms reflects the fact they "organisms phagocytose very different things" seems dubious because they all phagocytose bacteria*. *The argument from the differences between Entamoeba strains is curious; we would tend to believe that these differences reflect the malleable composition of the phagosomes not their different origins*.

### Minor comments

The authors state, that "plants cells are not phagocytic". This is mostly true (one exception is the prasinophyte green alga with surface scales cited in [104], however plants originated when a biciliate host phagocytosed a cyanobacterium. So the plant lineage is also ancestrally phagocytic.

*Authors' response: This is an interesting point but, strictly speaking, we cannot be sure that engulfment of cyanobacterial symbiont comprised bona fide phagocytosis*. In Fig. 4, *we put a question mark on the Plantae supergroup vis-à-vis its phagocytic ability, and it seems like we have to leave it at that*.

"To date no phagocytosis has been reported in fungi". One exception is the basal fungus *Rozella allomycis*, which can phagocytose organelles of its host [31].

*Authors' response: This is an interesting exception that we now mention in the revised Background section*.

Rhizaria can also be phagotrophic (e.g. some Foraminifera).

*Authors' response: We indicate in the Background section that phagocytosis of bacteria occurs in various unicellular eukaryotes which covers Rhizaria*. *However, it seems to make sense to keep the question mark in *Fig. 4*because the data are fragmentary*.

### Reviewer's report 3: Pierre Pontarotti

The authors carried out a comparative analysis between the phagosome of eukaryote from highly divergent phyla by using data from proteomic analyses and literature searches.

Authors found that several proteins have been conserved inferring that the corresponding genes were present in the common ancestor of the present days living eukaryotes and were involved in the ancestral phagocytosis.

To extend their analyses, they subsequently checked for the presence of these conserved eukaryote genes in archaea and bacteria.

They found that the orthologues of one of such a gene are present in subset of archaea. This gene is orthologous to eukaryotic actins gene and share unusual structural features with actins related proteins (Arp) 2 and 3. The authors deduced that the presence of common structural features in Arp/23 and the archea actins, implies that the common ancestors between the archaea and eukaryotic actins were able to develop branch filaments.

They further found that the orthologue of the Rho family appeared to be of bacterial origin.

These two findings lead the authors to hypothesize than the ancestor of the eukaryotes was an archeon that had an actin-based-cytoskeleton allowing engulfment of one bacteria (that will become the mitochondrion), horizontal transfer then occurred leading to eukaryogenesis.

I think that this hypothesis on the origin of the eukaryotic phyla is of great interest. However the authors' conclusion is only based on two genes (one from archea origin and the other from bacterial origin).

My first recommendation is to change the title as follows: "the possible origins of phagocytosis and eukaryogenesis" that should better describe the works of authors.

*Authors' response: Point made and noticed but we stick to the original title*. *Any analysis of the origins of a biological system deals with the possible rather than certain; we believe there is no need to state this explicitly*.

Other comments:

Concerning the" lack of phylogenetic coherence", the authors argue logically, that this could be due to imperfect detection of phagosomal proteins with the applied proteomic methods or variability of the phagocytosis machinery. One way to go further is to check the presence of the apparently absent proteins at genome level.

If the corresponding genes are found, then two explanations are possible

1) Gene co-option

2) The corresponding protein have been missed by the proteomic analysis

The authors could then label the protein as "probably involved in phagocytosis"

This approach will increase the number of conserved eukaryotic phagocytosis proteins. This enlarged set could be used to identify orthologues in bacteria and archea and could help to strengthen the author's hypothesis as suggested above.

*Authors' response: Effectively, we employ a very similar approach by including in the analysis proteins that were detected in subsets of the phagosomal proteomes*. *We chose not to relax the standard further*.

The authors wrote : "Crenarchaeota of the order of Thermoproteales and the only sequenced genome of Korarchaeum encode an actin homolog the eukaryotic actin"

Here the authors have to explain why some of the Crenarchaeta do not have actin genes?

If they hypothesize gene loss, did this loss happened several times, or did it happen once (the answer will be given by the tree topology)?

The next question is why the *Thermoproteales *retain these genes and not the other phyla of Crenarchaeta. Some discussion is required here.

*Authors' response: A brief discussion was added. 'Why' questions are, of course, hardly answerable*.

## Supplementary Material

Additional file 1 **The 118 conserved phagosomal proteins identified by comparison of 7 proteomics studies.** Click here for file

Additional file 2 **Amino acid sequences of phagosomal proteins classified by cluster**. Click here for file

Additional file 3 **Phyletic distribution of major proteins involved in phagocytosis.** Click here for file

Additional file 4 **BLAST results of 118 phagosomal protein clusters against RefSeq protein database**. Click here for file

Additional file 5 **Complete figure legends.** Click here for file

Additional file 6 **List of prokaryotic GTPases that cluster with the eukaryotic Rab, Ras, Ran, and Rho families.** Click here for file

Additional file 7 **Alignment of small GTPases used for the construction of the tree in Figure **3. Click here for file

Additional file 8 **Alignment of actin-like superfamily proteins used for the construction of the tree in Figure **1. Click here for file
