# Supplementary material for: The origins of phagocytosis and eukaryogenesis
Source: Biol Direct. 2009 Feb 26;4:9. doi: 10.1186/1745-6150-4-9 (PMC2651865; doi:10.1186/1745-6150-4-9)
Supplement: Additional file 1 — The 118 conserved phagosomal proteins identified by comparison of 7 proteomics studies. [file 1745-6150-4-9-S1.doc]

**Conserved phagosome proteins from proteomic data^a^**

| **Cluster ID** | **Present in species (out of 5)** | **Protein** | | **Comments** |
| --- | --- | --- | --- | --- |
| cytoskeleton-assosiated proteins | | | | |
| **3** | 3 | alpha actinin 4 | | actin filament crosslinker |
| **78** | 3 | filamin | | filamin proteins crosslink actin filaments into orthogonal networks in cortical cytoplasm and participate in the anchoring of membrane proteins for the actin cytoskeleton. |
| **84** | 3 | CAP, adenylate cyclase-associated protein 1 | | plays a key role in speeding up the turnover of actin filaments by effectively recycling cofilin and actin and through its effect on both ends of actin filament |
| **85** | 3 | F-actin capping protein alpha subunit | | the protein regulates growth of the actin filament by capping the barbed end of growing actin filaments |
| **95** | 3 | gelsolin-like capping protein | | the protein reversibly blocks the barbed ends of F-actin filaments in a Ca2+ and phosphoinositide-regulated manner |
| **102** | 3 | profilin 1 | | actin monomer-binding protein |
| **110** | 3 | spectrin beta chain | | Spectrin is an actin crosslinking and molecular scaffold protein that links the plasma membrane to the actin cytoskeleton |
| **96** | 4 | cofilin | | binds and depolymerizes filamentous F-actin |
| **27** | 3 | actin-related protein 3 (Arp3) | | actin nucleation |
| **59** | 4 | beta actin | |  |
| **20** | 4 | myosin, heavy polypeptide 9, non-muscle isoform 1 | | Class II myosin |
| **26** | 4 | tubulin, beta, 2 | |  |
| **62** | 5 | tubulin, alpha 1 | |  |
| signaling | | | | |
| **97** | 3 | annexin IV | | calcium-dependent phospholipid binding; cytoplasm |
| **56** | 3 | Rho GDP dissociation inhibitor (GDI) alpha | | Rho interactor |
| **15** | 3 | guanine nucleotide-binding protein, beta-2 subunit | | signal transduction |
| **60** | 3 | guanine nucleotide binding protein, alpha inhibiting 3 | | signal transduction |
| **93** | 4 | guanine nucleotide binding protein, beta polypeptide 2-like | | signal transduction |
| **92** | 3 | GDP dissociation inhibitor 2 (regulates the GDP-GTP exchange reaction of members of the rab family) | | Rab-related protein |
| **2** | 3 | Rab2 | | small GTPase |
| **11** | 3 | Rab22a | | small GTPase |
| **14** | 3 | Rab32 | | small GTPase |
| **18** | 3 | Rab14 | | small GTPase |
| **5** | 4 | Rap1b | | small GTPase |
| **29** | 4 | Rab10 | | small GTPase |
| **70** | 4 | Ras1 | | small GTPase |
| **90** | 4 | Rab11 | | small GTPase |
| **22** | 5 | Rab1a | | small GTPase |
| **25** | 5 | Rab7 | | small GTPase |
| **114** | 4 | Sar1 | | small GTPase |
| vesicular transport | | | | |
| **38** | 3 | N-ethylmaleimide sensitive fusion protein (NSF) | | NSF interacts with alpha-SNAP |
| **13** | 3 | alpha SNAP (Soluble NSF Attachment Protein) | | NSF and SNAPs are general elements of the cellular membrane transport apparatus. |
| **77** | 3 | gamma SNAP (Soluble NSF Attachment Protein) | |  |
| **40** | 3 | Synaptobrevin | | SNAP receptor activity |
| **42** | 3 | syntaxin 7 | | SNAP receptor activity |
| **83** | 5 | ADP ribosylation factor 79F CG8385-PB, isoform B. Arf1-Arf5-like subfamily. | | cell adhesion, endocytosis |
| **72** | 3 | Receptor mediated endocytosis 8 CG8014-PA | | clathrin-dependent endocytosis |
| **116** | 3 | GRAM domain-containing protein (v-SNARE family protein) | | endocytosis |
| **58** | 3 | vesicle-associated membrane protein/Synaptobrevin/VAMP-like protein | | vesicle fusion; SNARE interactor |
| **35** | 4 | Vacuolar protein sorting-associated protein | | Intracellular trafficking and secretion |
| **80** | 3 | clathrin heavy chain | |  |
| vacuolar acidification | | | | |
| **6** | 5 | ATPase, H+ transporting, lysosomal V0 subunit A | |  |
| **53** | 3 | ATPase, H+ transporting, lysosomal V0 subunit H | |  |
| **69** | 3 | ATPase, H+ transporting, lysosomal V1 subunit E | |  |
| **52** | 4 | ATPase, H+ transporting, lysosomal V0 subunit D | |  |
| **9** | 3 | ATPase, H+ transporting, lysosomal V1 subunit D | |  |
| **19** | 4 | ATPase, H transporting, lysosomal V1 subunit B2 | |  |
| **48** | 4 | ATPase, H transporting, lysosomal V1 subunit A | |  |
| hydrolytic enzymes | | | | |
| **91** | 3 | alpha glucosidase | | lysosomal hydrolase |
| **109** | 3 | S-adenosylhomocysteine hydrolase | | cytoplasmic hydrolase |
| **17** | 3 | lysosomal thiol reductase, gamma-interferon indusible (GILT) | | lysosomal protein |
| **1** | 4 | cathepsin | | peptidase |
| **103** | 3 | ErbB3-binding protein 1 | | proteolysis |
| ER proteins | | | | |
| **54** | 3 | EH-domain containing 4 | | involved in regulation of endocytosis |
| **117** | 3 | EF-hand calcium-binding domain containing protein | |  |
| **94** | 4 | Calreticulin | | Calreticulin is a multifunctional protein that acts as a major Ca(2+)-binding (storage) protein in the lumen of the endoplasmic reticulum. |
| **37** | 3 | ATPase, Ca++ transporting, cardiac muscle, slow twitch 2 | | Sarcoplasmic/endoplasmic reticulum protein |
| **39** | 3 | Ribophorin I, dolychil-diphosphooligosaccharide- protein glycosyltransferase subunit | | This gene encodes a type I integral membrane protein found only in the rough endoplasmic reticulum. |
| **34** | 4 | Pdia6 protein disulfide isomerase associated 6 | |  |
| **67** | 5 | protein disulfide isomerase | | an ER-resident protein |
| protein folding | | | | |
| **86** | 3 | chaperonin containing TCP1, subunit 2 (beta) | |  |
| **98** | 3 | chaperonin containing TCP1, subunit 3 (gamma) | |  |
| **99** | 3 | chaperonin containing TCP1, subunit 4 (delta) | |  |
| **100** | 3 | chaperonin containing TCP1, subunit 6a (zeta1) | |  |
| **101** | 3 | heat shock 70kDa protein 9 | |  |
| **115** | 3 | heat shock protein Hsp70 family protein | |  |
| **55** | 5 | heat shock 70kDa protein 5 | |  |
| **105** | 3 | heat shock protein 90kDa beta, member 1 | |  |
| **66** | 4 | heat shock protein Hsp90 | |  |
| **47** | 4 | heat shock 70kD protein 1-like | |  |
| **16** | 3 | stress-induced-phosphoprotein 1 (Hsp70/Hsp90-organizing protein) | |  |
| **111** | 3 | DnaJ (Hsp40) homolog | | works as a cochaperone of Hsp70s |
| translation | | | | |
| **79** | 3 | similar to ribosomal protein S15a | | ribosomal proteins |
| **7** | 3 | ribosomal protein L23 | | ribosomal proteins |
| **12** | 3 | ribosomal protein L9 | | ribosomal proteins |
| **21** | 3 | ribosomal protein S25 | | ribosomal proteins |
| **21** | 3 | ribosomal protein S8 | | ribosomal proteins |
| **50** | 3 | ribosomal protein S3a | | ribosomal proteins |
| **51** | 3 | ribosomal protein S14 | | ribosomal proteins |
| **68** | 3 | 60s Acidic ribosomal protein | | ribosomal proteins |
| **76** | 3 | ribosomal protein S12 | | ribosomal proteins |
| **87** | 3 | ribosomal protein S5 | | ribosomal proteins |
| **104** | 3 | ribosomal protein S3 | | ribosomal proteins |
| **107** | 3 | ribosomal protein S16 | | ribosomal proteins |
| **44** | 4 | ribosomal protein SA | | ribosomal proteins |
| **82** | 4 | ribosomal protein P0 | | ribosomal proteins |
| **81** | 4 | ubiquitin A-52 residue ribosomal protein fusion product 1 | | ribosome/translation elongation |
| **24** | 4 | eukaryotic translation initiation factor 4A | | translation initiation |
| **30** | 3 | eEF1gamma | | translational elongation |
| **8** | 4 | eEF1delta | | translational elongation |
| **61** | 4 | eEF-2 | | translational elongation |
| **65** | 5 | eEF-alpha1 | | translational elongation |
| other proteins | | | | |
| **106** | 3 | voltage-dependent anion channel 2 | | mitochondrial outer membrane |
| **10** | 3 | cytochrome b-5 | | mitochondrial membrane |
| **4** | 3 | ATP synthase, H+ transporting, mitochondrial F1 complex, gamma subunit | | mitochondrial membrane |
| **49** | 3 | ATP synthase, H+ transporting, mitochondrial F1 complex, beta subunit | | mitochondrial membrane |
| **118** | 3 | Rae1 (WD40 repeat-containing protein) | | mRNA export from nucleus |
| **45** | 4 | poly A binding protein, cytoplasmic 1 | | mRNA processing |
| **57** | 3 | fibrillarin | | rRNA processing |
| **46** | 3 | alpha-amylase family protein/solute carrier family 3 | | catalytic activity |
| **71** | 3 | acyl-CoA synthetase long-chain family member 4 (ER ) | | fatty acid metabolic process |
| **74** | 3 | phosphoglycerate kinase/calmodulin-binding protein | | glycolysis |
| **75** | 5 | glyceraldehyde-3-phosphate dehydrogenase | | glycolysis |
| **88** | 3 | transketolase | | metabolic |
| **112** | 3 | NAD-dependent epimerase/dehydratase family protein | | metabolic |
| **63** | 3 | nucleoside-diphosphate kinase | | nucleotide metabolism |
| **36** | 3 | phosphoglycerate dehydrogenase | | serine biosynthesis |
| **28** | 3 | isocitrate dehydrogenase 2 (NADP+), mitochondrial | | tricarboxylic acid cycle |
| **89** | 4 | ATP-binding cassette, sub-family C (CFTR/MRP), member 1 | | outer and inner membranes |
| **43** | 3 | peroxiredoxin 4: thioredoxin peroxidase | | peroxidase; cytoplasm |
| **113** | 3 | 26S protease regulatory subunit 4 | | proteasome |
| **23** | 3 | Na,K-ATPase alpha-3-subunit | | plasma membrane |
| **73** | 3 | Plasma membrane calcium-transporting ATPase | | plasma membrane |
| **31** | 3 | PREDICTED: similar to germinal histone H4 gene | |  |
| **32** | 3 | PREDICTED: similar to Histone H2B | |  |
| **41** | 4 | PREDICTED: similar to tyrosine 3/tryptophan 5 -monooxygenase activation protein , zeta polypeptide | |  |
| **64** | 4 | PREDICTED: similar to peptidylprolyl isomerase A-like |  | |
|  |  |  | |  |

^a^The cluster ID correspond to the clusters in Additional File 3
