# Supplementary material for: The origins of phagocytosis and eukaryogenesis
Source: Biol Direct. 2009 Feb 26;4:9. doi: 10.1186/1745-6150-4-9 (PMC2651865; doi:10.1186/1745-6150-4-9)
Supplement: Additional file 3 — Phyletic distribution of major proteins involved in phagocytosis. [file 1745-6150-4-9-S3.doc]

|  | **query (gi)** |  |  |  |  |  |  |  |  |  |  |  |  |
| --- | --- | --- | --- | --- | --- | --- | --- | --- | --- | --- | --- | --- | --- |
| **Receptors** | 204121 | - | - | - | - | - | - | - | - | - | yes - mammals IG domains - all animals | - | - |
| Fcγ |
| CR3, subunit α_M_ | 88501734 | - | - | - | - | - | - | - | - | - | yes | - | - |
| CR3, subunit β_2_ | 124056465 | - | - | - | - | - | - | - | - | - | yes | - | - |
| EGF receptor | 6478868 | - | - | - | - | - | - | - | - | - | yes | - | - |
| Mannose receptor | 109895388 | - | CLECT domains | CLECT domains | - | - | - | - | - | CLECT domains | yes-chordata; others: CLECT domains | CLECT domains | - |
| CED-1/MEGF10 | 12597465 | - | - | - | - | - | - | - | - | - | yes | - | - |
| SibA-SibD | 60465670 | - | - | - | - | yes | - | - | - | - | - | - | - |
| TM9 protein (Phg1) | 74859302 | yes | yes | yes | yes | yes | - | - | yes | yes | yes | yes | yes |
| SadA | 60465074 | - | - | - | - | yes | - | - | - | - | - | - | - |
| **Actin-remodeling** | |  |  |  |  |  |  |  |  |  |  |  |  |
| Actin | 66826069 | yes | yes | yes | yes | yes | yes | yes | yes | yes | yes | yes | yes |
| ARP2 | 4093161 | yes | yes | yes | yes | yes | - | yes | yes | yes | yes | yes | yes |
| ARP3 | 60467470 | yes | yes | yes | yes | yes | - | yes | yes | yes | yes | yes | yes |
| ARPC1 | 66816255 | yes | - | yes | yes | yes | - | yes | yes | yes | yes | yes | yes |
| ARPC2 | 60467975 | - | yes | yes | yes | yes | - | yes | yes | yes | yes | yes | yes |
| ARPC3 | 50344884 | - | yes | yes | yes | yes | - | yes | yes | yes | yes | yes | yes |
| ARPC4 | 115495705 | yes | yes | yes | yes | yes | - | yes | yes | yes | yes | yes | yes |
| ARPC5 | 66806101 | - | - | - | - | yes | - | yes | yes | yes | yes | yes | yes |
| WASp | 10880935 | - | - | yes | yes | yes | - | yes | yes | - | yes | - | yes |
| WAVE1/ SCAR1 | 66809177 | - | - | yes | - | yes | - | - | - | - | yes | yes | yes |
| Profilin | 730406 | yes | yes | yes | yes | yes | - | yes | yes | yes | yes | yes | yes |
| Formin | 158518557 | yes | yes | yes | yes | yes | - | yes | yes | yes | yes | yes | yes |
| Cofilin/ADF | 3182971 | yes | yes | yes | yes | yes | - | yes | yes | yes | yes | yes | yes |
| Coronin | 11023 | yes | - | yes | yes | yes | - | yes | yes | yes | yes | - | yes |
| alpha-actinin | 60474969 | EFh domains | EFh domains | yes | EFh domains | yes | EFh domains | yes | yes | EFh domains | yes | EFh domains | yes |
| Filamin |  | CH domains | CH domains | CH domains | CH domains | yes | - | - | CH domains | - | yes | CH domains | CH domains |
| **Rho GTPases** |  |  |  |  |  |  |  |  |  |  |  |  |  |
| Cdc42 | 45384262 | - | - | yes | - | - | - | - | yes | - | yes | - | - |
| Rac/RhoG/CED-10 | 2702398 | - | yes | yes | yes | yes | yes | yes | yes | - | yes | yes | yes |
| RhoA | 2225894 | - | - | yes | - | - | - | - | yes | - | yes | - | - |
