# Supplementary material for: The origins of phagocytosis and eukaryogenesis
Source: Biol Direct. 2009 Feb 26;4:9. doi: 10.1186/1745-6150-4-9 (PMC2651865; doi:10.1186/1745-6150-4-9)
Supplement: Additional file 4 — BLAST results of 118 phagosomal protein clusters against RefSeq protein database. [file 1745-6150-4-9-S4.doc]

|  |  |  |  |  |  |  |  |  |  |  |  |  |  |
| --- | --- | --- | --- | --- | --- | --- | --- | --- | --- | --- | --- | --- | --- |

**Homologs of phagosomal proteins (Table 1 and Additional File 2)**

| Homologs of receptor proteins | | | |
| --- | --- | --- | --- |
| (only the best hit from each species is shown) | | |  |
| Fcγ | query:204121 | hit gi | bit score |
| Metazoa | Bos_taurus | 157954059 | 237 |
| Metazoa | Canis_lupus_familiaris | 74006258 | 186 |
| Metazoa | Equus_caballus | 194210504 | 268 |
| Metazoa | Felis_catus | 57163737 | 191 |
| Metazoa | Homo_sapiens | 210031822 | 219 |
| Metazoa | Macaca_mulatta | 109017834 | 226 |
| Metazoa | Monodelphis_domestica | 126306222 | 201 |
| Metazoa | Mus_musculus | 190570188 | 392 |
| Metazoa | Ornithorhynchus_anatinus | 149617830 | 158 |
| Metazoa | Ovis_aries | 213021144 | 199 |
| Metazoa | Pan_troglodytes | 57114025 | 213 |
| Metazoa | Papio_anubis | 162951922 | 199 |
| Metazoa | Rattus_norvegicus | 31542804 | 560 |
| Metazoa | Sus_scrofa | 74136759 | 206 |
|  |  |  |  |
| CR3, subunit α_M_ | 88501734 | hit gi | bit score |
| Metazoa | Acyrthosiphon_pisum | 193617910 | 148 |
| Metazoa | Aedes_aegypti | 157120744 | 146 |
| Metazoa | Anopheles_gambiae_str-_PEST | 158289018 | 152 |
| Metazoa | Apis_mellifera | 110761776 | 162 |
| Metazoa | Bos_taurus | 90403616 | 1837 |
| Metazoa | Caenorhabditis_briggsae_AF16 | 157775997 | 152 |
| Metazoa | Caenorhabditis_elegans | 17553598 | 153 |
| Metazoa | Canis_lupus_familiaris | 73958268 | 1825 |
| Metazoa | Ciona_intestinalis | 198421589 | 243 |
| Metazoa | Culex_quinquefasciatus | 170033776 | 152 |
| Metazoa | Danio_rerio | 189515501 | 478 |
| Metazoa | Drosophila_erecta | 194882867 | 110 |
| Metazoa | Drosophila_grimshawi | 195046881 | 138 |
| Metazoa | Drosophila_melanogaster | 19922826 | 102 |
| Metazoa | Drosophila_mojavensis | 195132536 | 137 |
| Metazoa | Drosophila_sechellia | 195347134 | 103 |
| Metazoa | Drosophila_simulans | 195583600 | 107 |
| Metazoa | Drosophila_virilis | 195398867 | 144 |
| Metazoa | Drosophila_willistoni | 195426531 | 108 |
| Metazoa | Equus_caballus | 149725809 | 1847 |
| Metazoa | Felis_catus | 57163755 | 367 |
| Metazoa | Gallus_gallus | 45384200 | 364 |
| Metazoa | Homo_sapiens | 88501734 | 2385 |
| Metazoa | Macaca_mulatta | 109128363 | 709 |
| Metazoa | Monodelphis_domestica | 126334040 | 1446 |
| Metazoa | Mus_musculus | 132626295 | 1818 |
| Metazoa | Nasonia_vitripennis | 156552325 | 148 |
| Metazoa | Nematostella_vectensis | 156407206 | 128 |
| Metazoa | Ornithorhynchus_anatinus | 149482204 | 923 |
| Metazoa | Oryctolagus_cuniculus | 126722770 | 125 |
| Metazoa | Ovis_aries | 128485808 | 1830 |
| Metazoa | Pan_troglodytes | 114662186 | 1675 |
| Metazoa | Rattus_norvegicus | 29789036 | 1787 |
| Metazoa | Strongylocentrotus_purpuratus | 47550937 | 181 |
| Metazoa | Sus_scrofa | 113205876 | 558 |
| Metazoa | Tribolium_castaneum | 189240779 | 173 |
| Metazoa | Trichoplax_adhaerens | 196002659 | 147 |
| Metazoa | Xenopus_-Silurana-_tropicalis | 183986665 | 147 |
| Metazoa | Xenopus_laevis | 148233336 | 172 |
|  |  |  |  |
| CR3, subunit β_2_ | 124056465 | hit gi | bit score |
| Metazoa | Acyrthosiphon_pisum | 193702351 | 513 |
| Metazoa | Aedes_aegypti | 157132647 | 482 |
| Metazoa | Anopheles_gambiae_str-_PEST | 58381607 | 503 |
| Metazoa | Apis_mellifera | 110749786 | 478 |
| Metazoa | Bos_taurus | 41386721 | 1278 |
| Metazoa | Brugia_malayi | 170581270 | 514 |
| Metazoa | Caenorhabditis_elegans | 17554380 | 505 |
| Metazoa | Canis_lupus_familiaris | 73948624 | 675 |
| Metazoa | Ciona_intestinalis | 198420437 | 465 |
| Metazoa | Culex_quinquefasciatus | 170040959 | 484 |
| Metazoa | Danio_rerio | 189523718 | 744 |
| Metazoa | Drosophila_ananassae | 194763659 | 486 |
| Metazoa | Drosophila_erecta | 194897175 | 478 |
| Metazoa | Drosophila_grimshawi | 195048377 | 487 |
| Metazoa | Drosophila_melanogaster | 24640486 | 494 |
| Metazoa | Drosophila_mojavensis | 195129790 | 483 |
| Metazoa | Drosophila_persimilis | 195155395 | 338 |
| Metazoa | Drosophila_pseudoobscura_pseudoobscura | 198471235 | 481 |
| Metazoa | Drosophila_sechellia | 195355785 | 489 |
| Metazoa | Drosophila_simulans | 195565679 | 486 |
| Metazoa | Drosophila_virilis | 195397321 | 467 |
| Metazoa | Drosophila_willistoni | 195447350 | 482 |
| Metazoa | Drosophila_yakuba | 195480254 | 481 |
| Metazoa | Equus_caballus | 194226333 | 1291 |
| Metazoa | Felis_catus | 114326428 | 688 |
| Metazoa | Gallus_gallus | 46048728 | 1011 |
| Metazoa | Homo_sapiens | 89191865 | 1582 |
| Metazoa | Macaca_mulatta | 109116527 | 557 |
| Metazoa | Monodelphis_domestica | 126314716 | 1174 |
| Metazoa | Mus_musculus | 111607447 | 1257 |
| Metazoa | Nasonia_vitripennis | 156538655 | 486 |
| Metazoa | Nematostella_vectensis | 156407272 | 491 |
| Metazoa | Oncorhynchus_mykiss | 185133705 | 743 |
| Metazoa | Ornithorhynchus_anatinus | 149634686 | 687 |
| Metazoa | Oryctolagus_cuniculus | 126723613 | 553 |
| Metazoa | Ovis_aries | 57164157 | 1276 |
| Metazoa | Pan_troglodytes | 77628023 | 1576 |
| Metazoa | Pongo_abelii | 197098822 | 679 |
| Metazoa | Rattus_norvegicus | 163937849 | 1267 |
| Metazoa | Strongylocentrotus_purpuratus | 47551111 | 485 |
| Metazoa | Sus_scrofa | 47522672 | 1295 |
| Metazoa | Tribolium_castaneum | 189237209 | 481 |
| Metazoa | Trichoplax_adhaerens | 196001565 | 379 |
| Metazoa | Xenopus_-Silurana-_tropicalis | 45361049 | 694 |
| Metazoa | Xenopus_laevis | 148231811 | 926 |
|  |  |  |  |
| EGF receptor | 6478868 | hit gi | bit score |
| Metazoa | Acyrthosiphon_pisum | 193599120 | 431 |
| Metazoa | Bos_taurus | 119920730 | 2115 |
| Metazoa | Caenorhabditis_briggsae_AF16 | 157749015 | 256 |
| Metazoa | Caenorhabditis_elegans | 212645651 | 275 |
| Metazoa | Canis_lupus_familiaris | 73981874 | 2175 |
| Metazoa | Ciona_intestinalis | 118344322 | 655 |
| Metazoa | Danio_rerio | 35903183 | 1488 |
| Metazoa | Equus_caballus | 194209568 | 2221 |
| Metazoa | Felis_catus | 114326433 | 1173 |
| Metazoa | Gallus_gallus | 158261996 | 1919 |
| Metazoa | Homo_sapiens | 29725609 | 2219 |
| Metazoa | Macaca_mulatta | 109066534 | 2011 |
| Metazoa | Monodelphis_domestica | 126336558 | 2025 |
| Metazoa | Mus_musculus | 46560582 | 2402 |
| Metazoa | Ornithorhynchus_anatinus | 149588160 | 2035 |
| Metazoa | Pan_troglodytes | 114613399 | 2211 |
| Metazoa | Pongo_abelii | 197100534 | 986 |
| Metazoa | Rattus_norvegicus | 25742617 | 2519 |
| Metazoa | Strongylocentrotus_purpuratus | 115717889 | 350 |
| Metazoa | Sus_scrofa | 47522840 | 2143 |
| Metazoa | Tribolium_castaneum | 189235981 | 438 |
| Metazoa | Xenopus_laevis | 147906005 | 1234 |
|  |  |  |  |
| Mannose receptor | 109895388 | hit gi | bit score |
| Metazoa | Homo_sapiens | 57546917 | 3035 |
| Metazoa | Pan_troglodytes | 114629636 | 3028 |
| Metazoa | Macaca_mulatta | 109088318 | 2996 |
| Metazoa | Equus_caballus | 149743690 | 2710 |
| Metazoa | Canis_lupus_familiaris | 73948954 | 2679 |
| Metazoa | Rattus_norvegicus | 157822935 | 2536 |
| Metazoa | Mus_musculus | 6678932 | 2514 |
| Metazoa | Monodelphis_domestica | 126341509 | 2156 |
| Metazoa | Gallus_gallus | 118085740 | 1902 |
| Metazoa | Bos_taurus | 119905293 | 966 |
| Metazoa | Danio_rerio | 189515935 | 903 |
| Metazoa | Xenopus_-Silurana-_tropicalis | 148231754 | 797 |
| Metazoa | Oryctolagus_cuniculus | 126723690 | 583 |
| Metazoa | Pongo_abelii | 197098170 | 579 |
| Metazoa | Ornithorhynchus_anatinus | 149639599 | 566 |
| Metazoa | Strongylocentrotus_purpuratus | 115916072 | 496 |
| Metazoa | Ciona_intestinalis | 198416474 | 457 |
|  |  |  |  |
| CED-1/MEGF-10 | 12597465 | hit gi | bit score |
| Metazoa | Acyrthosiphon_pisum | 193641183 | 100 |
| Metazoa | Aedes_aegypti | 157118156 | 391 |
| Metazoa | Anopheles_gambiae_str-_PEST | 158285971 | 388 |
| Metazoa | Apis_mellifera | 110755423 | 97.8 |
| Metazoa | Bos_taurus | 194668350 | 493 |
| Metazoa | Caenorhabditis_briggsae_AF16 | 157748387 | 1676 |
| Metazoa | Caenorhabditis_elegans | 25143262 | 2224 |
| Metazoa | Canis_lupus_familiaris | 74000897 | 516 |
| Metazoa | Ciona_intestinalis | 198428172 | 326 |
| Metazoa | Culex_quinquefasciatus | 170031028 | 416 |
| Metazoa | Danio_rerio | 189524072 | 493 |
| Metazoa | Drosophila_ananassae | 194747068 | 381 |
| Metazoa | Drosophila_erecta | 194864950 | 389 |
| Metazoa | Drosophila_grimshawi | 195012278 | 415 |
| Metazoa | Drosophila_melanogaster | 161080573 | 408 |
| Metazoa | Drosophila_mojavensis | 195135366 | 433 |
| Metazoa | Drosophila_persimilis | 195165737 | 88.2 |
| Metazoa | Drosophila_pseudoobscura_pseudoobscura | 198466772 | 355 |
| Metazoa | Drosophila_sechellia | 195336692 | 391 |
| Metazoa | Drosophila_virilis | 195375203 | 428 |
| Metazoa | Drosophila_willistoni | 195427197 | 353 |
| Metazoa | Drosophila_yakuba | 195490535 | 395 |
| Metazoa | Equus_caballus | 194210914 | 89.7 |
| Metazoa | Gallus_gallus | 118095990 | 497 |
| Metazoa | Homo_sapiens | 148833508 | 90.5 |
| Metazoa | Macaca_mulatta | 109014712 | 84.3 |
| Metazoa | Monodelphis_domestica | 126277452 | 507 |
| Metazoa | Mus_musculus | 50233828 | 493 |
| Metazoa | Nasonia_vitripennis | 156543784 | 395 |
| Metazoa | Nematostella_vectensis | 156378596 | 88.2 |
| Metazoa | Oncorhynchus_mykiss | 208973220 | 90.1 |
| Metazoa | Ornithorhynchus_anatinus | 149412159 | 493 |
| Metazoa | Pan_troglodytes | 114550734 | 436 |
| Metazoa | Rattus_norvegicus | 201066348 | 339 |
| Metazoa | Strongylocentrotus_purpuratus | 115751484 | 414 |
| Metazoa | Sus_scrofa | 178056526 | 89 |
| Metazoa | Tribolium_castaneum | 189234402 | 401 |
| Metazoa | Trichoplax_adhaerens | 196006862 | 92 |
| Metazoa | Xenopus_-Silurana-_tropicalis | 118404388 | 491 |
| Metazoa | Xenopus_laevis | 148227866 | 99.4 |
|  |  |  |  |
| SibA-SibD | 60465670 | hit gi | bit score |
| Mycetozoa | Dictyostelium_discoideum_AX4 | 66807087 | 3843 |
|  |  |  |  |
| TM9 protein (Phg1) | 74859302 | hit gi | bit score |
| Apicomplexa | Cryptosporidium_hominis_TU502 | 67624161 | 159 |
| Apicomplexa | Cryptosporidium_muris_RN66 | 209881289 | 186 |
| Apicomplexa | Cryptosporidium_parvum_Iowa_II | 66362518 | 160 |
| Apicomplexa | Plasmodium_berghei_str-_ANKA | 68070329 | 266 |
| Apicomplexa | Plasmodium_chabaudi_chabaudi | 70951084 | 132 |
| Apicomplexa | Plasmodium_falciparum_3D7 | 124802513 | 296 |
| Apicomplexa | Plasmodium_vivax_SaI-1 | 156093415 | 318 |
| Apicomplexa | Plasmodium_yoelii_yoelii_str-_17XNL | 83286350 | 172 |
| Chlorophyta | Chlamydomonas_reinhardtii | 159464629 | 632 |
| Chlorophyta | Ostreococcus_lucimarinus_CCE9901 | 145341564 | 570 |
| Choanoflagellida | Monosiga_brevicollis_MX1 | 167526331 | 506 |
| Ciliophora | Paramecium_tetraurelia_strain_d4-2 | 145537824 | 290 |
| Ciliophora | Tetrahymena_thermophila_SB210 | 146185435 | 264 |
| Fungi | Ajellomyces_capsulatus_NAm1 | 154270913 | 443 |
| Fungi | Ashbya_gossypii_ATCC_10895 | 45201192 | 360 |
| Fungi | Aspergillus_clavatus_NRRL_1 | 121700384 | 433 |
| Fungi | Aspergillus_fumigatus_Af293 | 70992901 | 462 |
| Fungi | Aspergillus_nidulans_FGSC_A4 | 67538560 | 388 |
| Fungi | Aspergillus_niger_CBS_513-88 | 145236597 | 459 |
| Fungi | Aspergillus_oryzae_RIB40 | 169785026 | 452 |
| Fungi | Aspergillus_terreus_NIH2624 | 115492785 | 447 |
| Fungi | Botryotinia_fuckeliana_B05-10 | 154293850 | 440 |
| Fungi | Candida_albicans_SC5314 | 68470978 | 404 |
| Fungi | Candida_glabrata_CBS_138 | 50285227 | 365 |
| Fungi | Chaetomium_globosum_CBS_148-51 | 116194832 | 457 |
| Fungi | Coccidioides_immitis_RS | 119196979 | 437 |
| Fungi | Coprinopsis_cinerea_okayama7-130 | 169847760 | 434 |
| Fungi | Cryptococcus_neoformans_var-_neoformans_JEC21 | 58267330 | 437 |
| Fungi | Debaryomyces_hansenii_CBS767 | 50422861 | 394 |
| Fungi | Gibberella_zeae_PH-1 | 46136299 | 450 |
| Fungi | Kluyveromyces_lactis_NRRL_Y-1140 | 50311797 | 381 |
| Fungi | Laccaria_bicolor_S238N-H82 | 170092311 | 456 |
| Fungi | Lodderomyces_elongisporus_NRRL_YB-4239 | 149236654 | 424 |
| Fungi | Magnaporthe_grisea_70-15 | 39972265 | 449 |
| Fungi | Malassezia_globosa_CBS_7966 | 164660684 | 420 |
| Fungi | Neosartorya_fischeri_NRRL_181 | 119473291 | 449 |
| Fungi | Neurospora_crassa_OR74A | 85107993 | 470 |
| Fungi | Penicillium_marneffei_ATCC_18224 | 212545933 | 456 |
| Fungi | Phaeosphaeria_nodorum_SN15 | 169599513 | 452 |
| Fungi | Pichia_guilliermondii_ATCC_6260 | 146419026 | 412 |
| Fungi | Pichia_stipitis_CBS_6054 | 150951603 | 392 |
| Fungi | Podospora_anserina_DSM_980 | 171683241 | 458 |
| Fungi | Pyrenophora_tritici-repentis_Pt-1C-BFP | 189211361 | 467 |
| Fungi | Saccharomyces_cerevisiae | 6323112 | 361 |
| Fungi | Schizosaccharomyces_pombe | 19113256 | 330 |
| Fungi | Sclerotinia_sclerotiorum_1980 | 156030871 | 423 |
| Fungi | Ustilago_maydis_521 | 71022187 | 418 |
| Fungi | Vanderwaltozyma_polyspora_DSM_70294 | 156846351 | 351 |
| Fungi | Yarrowia_lipolytica_CLIB122 | 50552888 | 421 |
| Kinetoplastida | Leishmania_braziliensis_MHOM-BR-75-M2904 | 154341443 | 320 |
| Kinetoplastida | Leishmania_infantum_JPCM5 | 146093161 | 305 |
| Kinetoplastida | Leishmania_major_strain_Friedlin | 73536814 | 295 |
| Kinetoplastida | Trypanosoma_brucei_TREU927 | 72392453 | 388 |
| Kinetoplastida | Trypanosoma_cruzi_strain_CL_Brener | 71655013 | 388 |
| Metazoa | Acyrthosiphon_pisum | 193610460 | 489 |
| Metazoa | Aedes_aegypti | 157116654 | 572 |
| Metazoa | Anopheles_gambiae_str-_PEST | 58392199 | 553 |
| Metazoa | Apis_mellifera | 66519946 | 543 |
| Metazoa | Bos_taurus | 166063945 | 553 |
| Metazoa | Brugia_malayi | 170582238 | 379 |
| Metazoa | Caenorhabditis_briggsae_AF16 | 157746705 | 490 |
| Metazoa | Caenorhabditis_elegans | 71997610 | 491 |
| Metazoa | Canis_lupus_familiaris | 73992152 | 550 |
| Metazoa | Ciona_intestinalis | 198430655 | 563 |
| Metazoa | Culex_quinquefasciatus | 170039896 | 570 |
| Metazoa | Danio_rerio | 68374322 | 504 |
| Metazoa | Drosophila_ananassae | 194765741 | 539 |
| Metazoa | Drosophila_erecta | 194860459 | 544 |
| Metazoa | Drosophila_grimshawi | 195031355 | 546 |
| Metazoa | Drosophila_melanogaster | 19921282 | 545 |
| Metazoa | Drosophila_mojavensis | 195119037 | 544 |
| Metazoa | Drosophila_persimilis | 195164698 | 542 |
| Metazoa | Drosophila_pseudoobscura_pseudoobscura | 198473323 | 542 |
| Metazoa | Drosophila_sechellia | 195338261 | 546 |
| Metazoa | Drosophila_simulans | 195579042 | 526 |
| Metazoa | Drosophila_virilis | 195397678 | 542 |
| Metazoa | Drosophila_willistoni | 195436632 | 541 |
| Metazoa | Drosophila_yakuba | 195472687 | 545 |
| Metazoa | Equus_caballus | 194224293 | 529 |
| Metazoa | Gallus_gallus | 118100781 | 552 |
| Metazoa | Homo_sapiens | 164519076 | 551 |
| Metazoa | Macaca_mulatta | 109092414 | 551 |
| Metazoa | Monodelphis_domestica | 126293858 | 537 |
| Metazoa | Mus_musculus | 31542095 | 547 |
| Metazoa | Nasonia_vitripennis | 156543340 | 560 |
| Metazoa | Nematostella_vectensis | 156394075 | 531 |
| Metazoa | Ornithorhynchus_anatinus | 149411810 | 506 |
| Metazoa | Pan_troglodytes | 114681451 | 551 |
| Metazoa | Pongo_abelii | 197102576 | 551 |
| Metazoa | Rattus_norvegicus | 71043702 | 552 |
| Metazoa | Strongylocentrotus_purpuratus | 115901656 | 545 |
| Metazoa | Sus_scrofa | 194041781 | 255 |
| Metazoa | Tribolium_castaneum | 91083669 | 555 |
| Metazoa | Trichoplax_adhaerens | 196008523 | 538 |
| Metazoa | Xenopus_-Silurana-_tropicalis | 194332542 | 504 |
| Metazoa | Xenopus_laevis | 147898653 | 545 |
| Mycetozoa | Dictyostelium_discoideum_AX4 | 66828277 | 1320 |
| Streptophyta | Arabidopsis_thaliana | 15231359 | 644 |
| Streptophyta | Oryza_sativa_Japonica_Group | 115480625 | 604 |
| Streptophyta | Physcomitrella_patens_subsp-_patens | 168008150 | 634 |
| Streptophyta | Zea_mays | 212275586 | 616 |
| Trichomonada | Trichomonas_vaginalis_G3 | 123479424 | 177 |

Homologs of actin and actin-related proteins 2, 3:

| actins | |  | ARP2 | |  | ARP3 | |
| --- | --- | --- | --- | --- | --- | --- | --- |
| E9_Arath | 15227502 |  | E9_Arath | 15232057 |  | E9_Arath | 15222206 |
| E9_Arath | 18411737 |  | E9_Chlre | 159467853 |  | E9_Chlre | 159475477 |
| E9_Arath | 30688915 |  | E9_Orysa | 115476118 |  | E9_Orysa | 115447087 |
| E9_Chlre | 159490610 |  | E9_Ostlu | 145357127 |  | E9_Ostlu | 145340736 |
| E9_Phypa | 167999061 |  | E9_Phypa | 168005503 |  | E9_Phypa | 168028167 |
| Ec_Babbo | 156086988 |  | Ec_Parte | 145491405 |  | E9_Phypa | 168033210 |
| Ec_Babbo | 156089607 |  | Ec_Tetth | 118375955 |  | Ec_Parte | 145509144 |
| Ec_Crypa | 66361948 |  | Ej_Entdi | 167378396 |  | Ec_Parte | 145535816 |
| Ec_Parte | 124088257 |  | Ej_Enthi | 67484752 |  | Ec_Tetth | 118401606 |
| Ec_Parte | 124088259 |  | Ek_Leiin | 146084705 |  | Ej_Entdi | 167393331 |
| Ec_Parte | 145476747 |  | Ek_Trybr | 71749422 |  | Ej_Enthi | 67462416 |
| Ec_Parte | 145477751 |  | Ek_Trycr | 71413680 |  | Ek_Leiin | 146082170 |
| Ec_Parte | 145480553 |  | Ek_Trycr | 71654042 |  | Ek_Leima | 157866998 |
| Ec_Parte | 145522193 |  | El_Aedae | 157130317 |  | Ek_Trybr | 71744600 |
| Ec_Parte | 145529329 |  | El_Ajeca | 154271590 |  | Ek_Trycr | 71404601 |
| Ec_Parte | 145541908 |  | El_Anoga | 158287121 |  | El_Aedae | 157132019 |
| Ec_Parte | 145544979 |  | El_Apime | 66501030 |  | El_Ajeca | 154286014 |
| Ec_Parte | 145544983 |  | El_Ashgo | 45188189 |  | El_Anoga | 31210041 |
| Ec_Plabe | 68074079 |  | El_Aspcl | 121701315 |  | El_Apime | 66499731 |
| Ec_Plabe | 68076489 |  | El_Aspfu | 70995896 |  | El_Ashgo | 45198937 |
| Ec_Plafa | 124803677 |  | El_Aspni | 145240217 |  | El_Aspcl | 121713682 |
| Ec_Plavi | 156098392 |  | El_Aspni | 67516783 |  | El_Aspfu | 70997495 |
| Ec_Tetth | 118366913 |  | El_Aspor | 169763296 |  | El_Aspni | 145254855 |
| Ec_Tetth | 118377803 |  | El_Aspte | 115491989 |  | El_Aspni | 67515717 |
| Ec_Tetth | 118386141 |  | El_Bosta | 119910986 |  | El_Aspor | 169775245 |
| Ec_Tetth | 146162481 |  | El_Bosta | 119910988 |  | El_Aspte | 115387575 |
| Ec_Tetth | 146165573 |  | El_Botfu | 154294286 |  | El_Bosta | 119891740 |
| Ec_Thepa | 71029966 |  | El_Bruma | 170583892 |  | El_Botfu | 154310843 |
| Ec_Thepa | 71033337 |  | El_Caebr | 157763344 |  | El_Bruma | 170579954 |
| Ei_Giala | 159108769 |  | El_Caeel | 17562290 |  | El_Caebr | 157746427 |
| Ej_Entdi | 167387355 |  | El_Canal | 68478986 |  | El_Caeel | 17510483 |
| Ej_Enthi | 67469701 |  | El_Cangl | 50291723 |  | El_Canal | 68482492 |
| Ej_Enthi | 67476230 |  | El_Canlu | 73969816 |  | El_Cangl | 50287133 |
| Ek_Leiin | 146103944 |  | El_Canlu | 73969820 |  | El_Canlu | 73979029 |
| Ek_Trybr | 71755105 |  | El_Chagl | 116197377 |  | El_Canlu | 73984196 |
| Ek_Trybr | 74025018 |  | El_Cocim | 119181589 |  | El_Chagl | 116204605 |
| Ek_Trycr | 71407107 |  | El_Copci | 169848997 |  | El_Cocim | 119174352 |
| Ek_Trycr | 71409880 |  | El_Cryne | 58271426 |  | El_Copci | 169859396 |
| Ek_Trycr | 71412228 |  | El_Cryne | 58271428 |  | El_Cryne | 58266690 |
| Ek_Trycr | 71415379 |  | El_Cryne | 58271430 |  | El_Culpi | 170060331 |
| Ek_Trycr | 71423070 |  | El_Culpi | 170028731 |  | El_Culpi | 170062922 |
| El_Aedae | 157106891 |  | El_Danre | 125832544 |  | El_Danre | 125853124 |
| El_Apime | 66509769 |  | El_Danre | 47087281 |  | El_Danre | 51556255 |
| El_Bosta | 119919421 |  | El_Danre | 56090180 |  | El_Debha | 50425535 |
| El_Bosta | 84579847 |  | El_Danre | 62955061 |  | El_Drome | 17737543 |
| El_Canlu | 57094217 |  | El_Drome | 24642545 |  | El_Drops | 125980470 |
| El_Canlu | 57112735 |  | El_Drops | 125981339 |  | El_Equca | 149707046 |
| El_Canlu | 73983553 |  | El_Equca | 149727877 |  | El_Equca | 149730579 |
| El_Canlu | 74003725 |  | El_Galga | 45382569 |  | El_Galga | 45383528 |
| El_Copci | 169867006 |  | El_Gibze | 46117286 |  | El_Galga | 50732241 |
| El_Danre | 71834558 |  | El_Homsa | 5031571 |  | El_Gibze | 46138683 |
| El_Drome | 17136990 |  | El_Homsa | 53692187 |  | El_Homsa | 5031573 |
| El_Drops | 125811884 |  | El_Klula | 50304825 |  | El_Homsa | 92373393 |
| El_Enccu | 85690961 |  | El_Lacbi | 170086460 |  | El_Homsa | 9966913 |
| El_Equca | 149758917 |  | El_Lacbi | 170086472 |  | El_Klula | 50304401 |
| El_Galga | 118091455 |  | El_Lodel | 149235241 |  | El_Lacbi | 170099361 |
| El_Galga | 118103141 |  | El_Macmu | 109103170 |  | El_Lodel | 149240065 |
| El_Galga | 50759347 |  | El_Macmu | 109103172 |  | El_Maggr | 145606953 |
| El_Galga | 50759349 |  | El_Maggr | 39971339 |  | El_Malgl | 164655465 |
| El_Homsa | 19923076 |  | El_Malgl | 164661235 |  | El_Monbr | 167523683 |
| El_Homsa | 29893808 |  | El_Monbr | 167533678 |  | El_Mondo | 126326219 |
| El_Homsa | 30425470 |  | El_Nasvi | 156544998 |  | El_Mondo | 126341250 |
| El_Homsa | 5729720 |  | El_Nemve | 156381398 |  | El_Musmu | 23956222 |
| El_Lacbi | 170106195 |  | El_Neucr | 85084895 |  | El_Musmu | 52345394 |
| El_Macmu | 109105103 |  | El_Ornan | 149640907 |  | El_Nasvi | 156537918 |
| El_Monbr | 167518279 |  | El_Pantr | 114577814 |  | El_Nemve | 156386369 |
| El_Monbr | 167526601 |  | El_Phano | 169614526 |  | El_Neofi | 119479019 |
| El_Mondo | 126323861 |  | El_Picgu | 146422936 |  | El_Neucr | 164428163 |
| El_Mondo | 126329466 |  | El_Picst | 150866137 |  | El_Ornan | 149634813 |
| El_Mondo | 126329468 |  | El_Podan | 171676539 |  | El_Pantr | 114580544 |
| El_Mondo | 126329470 |  | El_Ratno | 57164143 |  | El_Pantr | 114616879 |
| El_Mondo | 126333732 |  | El_Sacce | 6320175 |  | El_Pantr | 114616881 |
| El_Mondo | 126335099 |  | El_Schpo | 162312556 |  | El_Pantr | 114616885 |
| El_Mondo | 126335103 |  | El_Sclsc | 156066125 |  | El_Phano | 169623500 |
| El_Mondo | 126336048 |  | El_Strpu | 115921022 |  | El_Picgu | 146422234 |
| El_Mondo | 126338403 |  | El_Ustma | 71022645 |  | El_Picst | 150866153 |
| El_Musmu | 13385842 |  | El_Vanpo | 156839407 |  | El_Podan | 171689222 |
| El_Musmu | 13386316 |  | El_Xenla | 147902902 |  | El_Ratno | 62646620 |
| El_Musmu | 13386318 |  | El_Xenla | 148224409 |  | El_Sacce | 6322525 |
| El_Musmu | 82915635 |  | El_Xentr | 71896215 |  | El_Schpo | 19113810 |
| El_Musmu | 85662421 |  | El_Yarli | 50556094 |  | El_Sclsc | 156034240 |
| El_Nemve | 156395352 |  | Ev_Dicdi | 66823841 |  | El_Strpu | 72005268 |
| El_Ornan | 149410955 |  | Ew_Triva | 123433980 |  | El_Trica | 91078924 |
| El_Ornan | 149428572 |  |  |  |  | El_Ustma | 71005030 |
| El_Ornan | 149633051 |  |  |  |  | El_Vanpo | 156843209 |
| El_Ornan | 149633053 |  |  |  |  | El_Xenla | 147901950 |
| El_Ornan | 149641101 |  |  |  |  | El_Xentr | 45361583 |
| El_Podan | 171679245 |  |  |  |  | El_Yarli | 50554707 |
| El_Ratno | 109463286 |  |  |  |  | Ev_Dicdi | 66810313 |
| El_Ratno | 157822087 |  |  |  |  | Ew_Triva | 123491873 |
| El_Ratno | 62078523 |  |  |  |  |  |  |
| El_Ratno | 70794750 |  |  |  |  |  |  |
| El_Strpu | 115709892 |  |  |  |  |  |  |
| El_Strpu | 115915225 |  |  |  |  |  |  |
| El_Strpu | 72054363 |  |  |  |  |  |  |
| El_Strpu | 72144896 |  |  |  |  |  |  |
| El_Strpu | 72166108 |  |  |  |  |  |  |
| El_Xentr | 54606875 |  |  |  |  |  |  |
| Ev_Dicdi | 166240255 |  |  |  |  |  |  |
| Ev_Dicdi | 66804909 |  |  |  |  |  |  |
| Ev_Dicdi | 66804911 |  |  |  |  |  |  |
| Ev_Dicdi | 66807573 |  |  |  |  |  |  |
| Ev_Dicdi | 66809757 |  |  |  |  |  |  |
| Ev_Dicdi | 66821579 |  |  |  |  |  |  |
| Ev_Dicdi | 66825283 |  |  |  |  |  |  |
| Ev_Dicdi | 66825401 |  |  |  |  |  |  |
| Ev_Dicdi | 66826069 |  |  |  |  |  |  |
| Ew_Triva | 123400754 |  |  |  |  |  |  |
| Ew_Triva | 123418982 |  |  |  |  |  |  |
| Ew_Triva | 123456199 |  |  |  |  |  |  |
| Ew_Triva | 123477217 |  |  |  |  |  |  |
| Ew_Triva | 123485208 |  |  |  |  |  |  |
| Ew_Triva | 154411693 |  |  |  |  |  |  |
| Ew_Triva | 154413991 |  |  |  |  |  |  |
| Ew_Triva | 154418652 |  |  |  |  |  |  |
| Ew_Triva | 154420420 |  |  |  |  |  |  |

| Homologs of actin-remodeling proteins in (Table 1 and Additional File 2) | | | |
| --- | --- | --- | --- |
| (only the best hit from each species is shown) | | |  |
| ARP2/3 complex proteins: | |  |  |
| ARPC2 | query:60467975 | hit gi | bit score |
| Archamoebae | Entamoeba_dispar_SAW760 | 167388838 | 165 |
| Archamoebae | Entamoeba_histolytica_HM-1-IMSS | 67469155 | 165 |
| Chlorophyta | Ostreococcus_lucimarinus_CCE9901 | 145344425 | 115 |
| Choanoflagellida | Monosiga_brevicollis_MX1 | 167522521 | 198 |
| Ciliophora | Paramecium_tetraurelia_strain_d4-2 | 145499154 | 72 |
| Ciliophora | Tetrahymena_thermophila_SB210 | 118348072 | 82.4 |
| Fungi | Ajellomyces_capsulatus_NAm1 | 154280106 | 217 |
| Fungi | Ashbya_gossypii_ATCC_10895 | 45200876 | 164 |
| Fungi | Aspergillus_clavatus_NRRL_1 | 121703201 | 209 |
| Fungi | Aspergillus_fumigatus_Af293 | 70990336 | 208 |
| Fungi | Aspergillus_nidulans_FGSC_A4 | 67516049 | 209 |
| Fungi | Aspergillus_niger_CBS_513-88 | 145229333 | 205 |
| Fungi | Aspergillus_oryzae_RIB40 | 169766740 | 208 |
| Fungi | Aspergillus_terreus_NIH2624 | 115396726 | 209 |
| Fungi | Botryotinia_fuckeliana_B05-10 | 154311742 | 209 |
| Fungi | Candida_albicans_SC5314 | 68474671 | 166 |
| Fungi | Candida_glabrata_CBS_138 | 50288553 | 170 |
| Fungi | Chaetomium_globosum_CBS_148-51 | 116192473 | 162 |
| Fungi | Coccidioides_immitis_RS | 119189387 | 216 |
| Fungi | Coprinopsis_cinerea_okayama7-130 | 169849391 | 208 |
| Fungi | Cryptococcus_neoformans_var-_neoformans_JEC21 | 58267902 | 204 |
| Fungi | Debaryomyces_hansenii_CBS767 | 50419051 | 180 |
| Fungi | Gibberella_zeae_PH-1 | 46125353 | 194 |
| Fungi | Kluyveromyces_lactis_NRRL_Y-1140 | 50310755 | 162 |
| Fungi | Laccaria_bicolor_S238N-H82 | 170087976 | 208 |
| Fungi | Lodderomyces_elongisporus_NRRL_YB-4239 | 149245246 | 184 |
| Fungi | Magnaporthe_grisea_70-15 | 39945526 | 214 |
| Fungi | Malassezia_globosa_CBS_7966 | 164660933 | 166 |
| Fungi | Neosartorya_fischeri_NRRL_181 | 119497239 | 211 |
| Fungi | Neurospora_crassa_OR74A | 164427509 | 201 |
| Fungi | Penicillium_marneffei_ATCC_18224 | 212539646 | 213 |
| Fungi | Phaeosphaeria_nodorum_SN15 | 169603249 | 179 |
| Fungi | Pichia_guilliermondii_ATCC_6260 | 146414926 | 179 |
| Fungi | Pichia_stipitis_CBS_6054 | 150951492 | 186 |
| Fungi | Podospora_anserina_DSM_980 | 171684539 | 208 |
| Fungi | Pyrenophora_tritici-repentis_Pt-1C-BFP | 189203543 | 206 |
| Fungi | Saccharomyces_cerevisiae | 6324363 | 156 |
| Fungi | Schizosaccharomyces_japonicus_yFS275 | 213403724 | 195 |
| Fungi | Schizosaccharomyces_pombe | 19114815 | 207 |
| Fungi | Sclerotinia_sclerotiorum_1980 | 156061897 | 207 |
| Fungi | Ustilago_maydis_521 | 71017507 | 221 |
| Fungi | Vanderwaltozyma_polyspora_DSM_70294 | 156836977 | 175 |
| Fungi | Yarrowia_lipolytica_CLIB122 | 50543096 | 218 |
| Kinetoplastida | Trypanosoma_brucei_TREU927 | 72392943 | 80.9 |
| Kinetoplastida | Trypanosoma_cruzi_strain_CL_Brener | 71415260 | 88.2 |
| Metazoa | Acyrthosiphon_pisum | 193610703 | 200 |
| Metazoa | Aedes_aegypti | 157124993 | 224 |
| Metazoa | Anopheles_gambiae_str-_PEST | 58393230 | 221 |
| Metazoa | Apis_mellifera | 66542158 | 198 |
| Metazoa | Bos_taurus | 77736371 | 217 |
| Metazoa | Brugia_malayi | 170585422 | 199 |
| Metazoa | Caenorhabditis_briggsae_AF16 | 157772561 | 234 |
| Metazoa | Caenorhabditis_elegans | 25144430 | 226 |
| Metazoa | Canis_lupus_familiaris | 74005720 | 208 |
| Metazoa | Ciona_intestinalis | 198417921 | 224 |
| Metazoa | Culex_quinquefasciatus | 170051460 | 217 |
| Metazoa | Danio_rerio | 47086645 | 217 |
| Metazoa | Drosophila_ananassae | 194758998 | 220 |
| Metazoa | Drosophila_erecta | 194878938 | 219 |
| Metazoa | Drosophila_grimshawi | 195035677 | 216 |
| Metazoa | Drosophila_melanogaster | 19921584 | 219 |
| Metazoa | Drosophila_mojavensis | 195117956 | 213 |
| Metazoa | Drosophila_pseudoobscura_pseudoobscura | 125984544 | 219 |
| Metazoa | Drosophila_virilis | 195388134 | 85.1 |
| Metazoa | Drosophila_willistoni | 195436894 | 219 |
| Metazoa | Drosophila_yakuba | 195484721 | 220 |
| Metazoa | Equus_caballus | 194211306 | 209 |
| Metazoa | Gallus_gallus | 118093746 | 219 |
| Metazoa | Homo_sapiens | 5031599 | 218 |
| Metazoa | Macaca_mulatta | 109101026 | 211 |
| Metazoa | Monodelphis_domestica | 126337842 | 216 |
| Metazoa | Mus_musculus | 112363072 | 216 |
| Metazoa | Nasonia_vitripennis | 156540243 | 187 |
| Metazoa | Nematostella_vectensis | 156391179 | 236 |
| Metazoa | Ornithorhynchus_anatinus | 149490695 | 112 |
| Metazoa | Pan_troglodytes | 114583277 | 213 |
| Metazoa | Rattus_norvegicus | 157817229 | 218 |
| Metazoa | Sus_scrofa | 194043819 | 159 |
| Metazoa | Tribolium_castaneum | 189240330 | 197 |
| Metazoa | Trichoplax_adhaerens | 196001743 | 229 |
| Metazoa | Xenopus_-Silurana-_tropicalis | 54020777 | 216 |
| Metazoa | Xenopus_laevis | 148231189 | 219 |
| Mycetozoa | Dictyostelium_discoideum_AX4 | 66811280 | 600 |
| Streptophyta | Arabidopsis_thaliana | 18397679 | 165 |
| Streptophyta | Oryza_sativa_Japonica_Group | 115459394 | 78.2 |
| Streptophyta | Physcomitrella_patens_subsp-_patens | 168012382 | 148 |
| Trichomonada | Trichomonas_vaginalis_G3 | 123469900 | 133 |
| ARPC3 | 50344884 | hit gi | bit score |
| Archamoebae | Entamoeba_dispar_SAW760 | 167393138 | 128 |
| Archamoebae | Entamoeba_histolytica_HM-1-IMSS | 67462483 | 126 |
| Chlorophyta | Chlamydomonas_reinhardtii | 159472763 | 109 |
| Choanoflagellida | Monosiga_brevicollis_MX1 | 167524226 | 152 |
| Ciliophora | Paramecium_tetraurelia_strain_d4-2 | 145527380 | 44.7 |
| Ciliophora | Tetrahymena_thermophila_SB210 | 118361818 | 99.4 |
| Fungi | Ajellomyces_capsulatus_NAm1 | 154277186 | 156 |
| Fungi | Ashbya_gossypii_ATCC_10895 | 45199102 | 167 |
| Fungi | Aspergillus_clavatus_NRRL_1 | 121719322 | 157 |
| Fungi | Aspergillus_fumigatus_Af293 | 70985300 | 158 |
| Fungi | Aspergillus_nidulans_FGSC_A4 | 67902156 | 150 |
| Fungi | Aspergillus_niger_CBS_513-88 | 145251914 | 160 |
| Fungi | Aspergillus_oryzae_RIB40 | 169771861 | 159 |
| Fungi | Aspergillus_terreus_NIH2624 | 115443242 | 163 |
| Fungi | Botryotinia_fuckeliana_B05-10 | 154319181 | 163 |
| Fungi | Candida_albicans_SC5314 | 68483533 | 169 |
| Fungi | Candida_glabrata_CBS_138 | 50288289 | 173 |
| Fungi | Chaetomium_globosum_CBS_148-51 | 116205748 | 158 |
| Fungi | Coccidioides_immitis_RS | 119182765 | 153 |
| Fungi | Coprinopsis_cinerea_okayama7-130 | 169861620 | 157 |
| Fungi | Cryptococcus_neoformans_var-_neoformans_JEC21 | 58259914 | 172 |
| Fungi | Debaryomyces_hansenii_CBS767 | 50426369 | 180 |
| Fungi | Gibberella_zeae_PH-1 | 46109438 | 163 |
| Fungi | Kluyveromyces_lactis_NRRL_Y-1140 | 50303863 | 177 |
| Fungi | Laccaria_bicolor_S238N-H82 | 170090706 | 180 |
| Fungi | Lodderomyces_elongisporus_NRRL_YB-4239 | 149235863 | 179 |
| Fungi | Magnaporthe_grisea_70-15 | 145611544 | 164 |
| Fungi | Malassezia_globosa_CBS_7966 | 164659982 | 155 |
| Fungi | Neosartorya_fischeri_NRRL_181 | 119499177 | 142 |
| Fungi | Neurospora_crassa_OR74A | 85093400 | 161 |
| Fungi | Penicillium_marneffei_ATCC_18224 | 212541871 | 157 |
| Fungi | Phaeosphaeria_nodorum_SN15 | 169626365 | 163 |
| Fungi | Pichia_guilliermondii_ATCC_6260 | 146418461 | 175 |
| Fungi | Pichia_stipitis_CBS_6054 | 126137055 | 170 |
| Fungi | Podospora_anserina_DSM_980 | 171678177 | 157 |
| Fungi | Pyrenophora_tritici-repentis_Pt-1C-BFP | 189189276 | 169 |
| Fungi | Saccharomyces_cerevisiae | 6323402 | 171 |
| Fungi | Schizosaccharomyces_japonicus_yFS275 | 213406962 | 187 |
| Fungi | Schizosaccharomyces_pombe | 19113083 | 191 |
| Fungi | Sclerotinia_sclerotiorum_1980 | 156063436 | 166 |
| Fungi | Ustilago_maydis_521 | 71005654 | 157 |
| Fungi | Vanderwaltozyma_polyspora_DSM_70294 | 156839281 | 166 |
| Fungi | Yarrowia_lipolytica_CLIB122 | 50547315 | 183 |
| Kinetoplastida | Trypanosoma_brucei_TREU927 | 71747292 | 84 |
| Kinetoplastida | Trypanosoma_cruzi_strain_CL_Brener | 71421503 | 101 |
| Metazoa | Acyrthosiphon_pisum | 193687028 | 237 |
| Metazoa | Aedes_aegypti | 157106202 | 250 |
| Metazoa | Anopheles_gambiae_str-_PEST | 118794239 | 230 |
| Metazoa | Apis_mellifera | 110756153 | 245 |
| Metazoa | Bombyx_mori | 115292423 | 252 |
| Metazoa | Brugia_malayi | 170582393 | 200 |
| Metazoa | Caenorhabditis_briggsae_AF16 | 157772767 | 202 |
| Metazoa | Caenorhabditis_elegans | 17555640 | 196 |
| Metazoa | Canis_lupus_familiaris | 73994597 | 328 |
| Metazoa | Ciona_intestinalis | 198419698 | 232 |
| Metazoa | Culex_quinquefasciatus | 170056407 | 248 |
| Metazoa | Danio_rerio | 50344884 | 374 |
| Metazoa | Drosophila_ananassae | 194745001 | 240 |
| Metazoa | Drosophila_erecta | 194901134 | 233 |
| Metazoa | Drosophila_grimshawi | 195037805 | 235 |
| Metazoa | Drosophila_melanogaster | 45550754 | 225 |
| Metazoa | Drosophila_mojavensis | 195108161 | 239 |
| Metazoa | Drosophila_persimilis | 195152207 | 240 |
| Metazoa | Drosophila_sechellia | 195328617 | 234 |
| Metazoa | Drosophila_simulans | 195567278 | 223 |
| Metazoa | Drosophila_virilis | 195395500 | 240 |
| Metazoa | Drosophila_willistoni | 195454172 | 231 |
| Metazoa | Drosophila_yakuba | 195480948 | 223 |
| Metazoa | Gallus_gallus | 50756357 | 347 |
| Metazoa | Homo_sapiens | 5031597 | 340 |
| Metazoa | Macaca_mulatta | 109098700 | 323 |
| Metazoa | Monodelphis_domestica | 126305837 | 340 |
| Metazoa | Mus_musculus | 9790141 | 343 |
| Metazoa | Nasonia_vitripennis | 156536955 | 234 |
| Metazoa | Nematostella_vectensis | 156379446 | 260 |
| Metazoa | Ornithorhynchus_anatinus | 149408839 | 342 |
| Metazoa | Pan_troglodytes | 114576472 | 330 |
| Metazoa | Rattus_norvegicus | 157786926 | 345 |
| Metazoa | Strongylocentrotus_purpuratus | 72010493 | 258 |
| Metazoa | Tribolium_castaneum | 91084583 | 246 |
| Metazoa | Trichoplax_adhaerens | 196010311 | 208 |
| Metazoa | Xenopus_-Silurana-_tropicalis | 165973372 | 346 |
| Metazoa | Xenopus_laevis | 148227376 | 346 |
| Mycetozoa | Dictyostelium_discoideum_AX4 | 66800891 | 165 |
| Streptophyta | Arabidopsis_thaliana | 18406746 | 159 |
| Streptophyta | Oryza_sativa_Japonica_Group | 115445209 | 154 |
| Streptophyta | Physcomitrella_patens_subsp-_patens | 168038353 | 167 |
| Trichomonada | Trichomonas_vaginalis_G3 | 154422807 | 110 |
| ARPC4 | 115495705 | hit gi | bit score |
| Apicomplexa | Cryptosporidium_hominis_TU502 | 67610384 | 67 |
| Apicomplexa | Cryptosporidium_muris_RN66 | 209880103 | 67.8 |
| Apicomplexa | Cryptosporidium_parvum_Iowa_II | 66359390 | 65.9 |
| Archamoebae | Entamoeba_dispar_SAW760 | 167382750 | 216 |
| Archamoebae | Entamoeba_histolytica_HM-1-IMSS | 67464751 | 215 |
| Chlorophyta | Chlamydomonas_reinhardtii | 159466148 | 176 |
| Chlorophyta | Ostreococcus_lucimarinus_CCE9901 | 145340615 | 150 |
| Choanoflagellida | Monosiga_brevicollis_MX1 | 167538936 | 268 |
| Ciliophora | Paramecium_tetraurelia_strain_d4-2 | 145487262 | 163 |
| Ciliophora | Tetrahymena_thermophila_SB210 | 118377490 | 155 |
| Fungi | Ajellomyces_capsulatus_NAm1 | 154280529 | 247 |
| Fungi | Ashbya_gossypii_ATCC_10895 | 45185596 | 228 |
| Fungi | Aspergillus_clavatus_NRRL_1 | 121704060 | 252 |
| Fungi | Aspergillus_fumigatus_Af293 | 70984667 | 254 |
| Fungi | Aspergillus_nidulans_FGSC_A4 | 67903422 | 246 |
| Fungi | Aspergillus_niger_CBS_513-88 | 145247280 | 255 |
| Fungi | Aspergillus_oryzae_RIB40 | 169778833 | 245 |
| Fungi | Botryotinia_fuckeliana_B05-10 | 154294746 | 246 |
| Fungi | Candida_albicans_SC5314 | 68476003 | 157 |
| Fungi | Candida_glabrata_CBS_138 | 50293195 | 232 |
| Fungi | Chaetomium_globosum_CBS_148-51 | 116193819 | 257 |
| Fungi | Coccidioides_immitis_RS | 119181521 | 248 |
| Fungi | Coprinopsis_cinerea_okayama7-130 | 169847133 | 259 |
| Fungi | Cryptococcus_neoformans_var-_neoformans_B-3501A | 134110984 | 272 |
| Fungi | Cryptococcus_neoformans_var-_neoformans_JEC21 | 58266626 | 173 |
| Fungi | Debaryomyces_hansenii_CBS767 | 50422927 | 249 |
| Fungi | Gibberella_zeae_PH-1 | 46130630 | 238 |
| Fungi | Kluyveromyces_lactis_NRRL_Y-1140 | 50309525 | 232 |
| Fungi | Laccaria_bicolor_S238N-H82 | 170098300 | 272 |
| Fungi | Lodderomyces_elongisporus_NRRL_YB-4239 | 149246293 | 177 |
| Fungi | Magnaporthe_grisea_70-15 | 149210549 | 191 |
| Fungi | Neosartorya_fischeri_NRRL_181 | 119467234 | 253 |
| Fungi | Neurospora_crassa_OR74A | 85118953 | 259 |
| Fungi | Penicillium_marneffei_ATCC_18224 | 212526596 | 248 |
| Fungi | Phaeosphaeria_nodorum_SN15 | 169609004 | 247 |
| Fungi | Pichia_guilliermondii_ATCC_6260 | 146419090 | 221 |
| Fungi | Pichia_stipitis_CBS_6054 | 126138034 | 249 |
| Fungi | Podospora_anserina_DSM_980 | 171676195 | 258 |
| Fungi | Pyrenophora_tritici-repentis_Pt-1C-BFP | 189198636 | 248 |
| Fungi | Saccharomyces_cerevisiae | 6322839 | 231 |
| Fungi | Schizosaccharomyces_japonicus_yFS275 | 213405371 | 239 |
| Fungi | Schizosaccharomyces_pombe | 19115028 | 243 |
| Fungi | Sclerotinia_sclerotiorum_1980 | 156055652 | 253 |
| Fungi | Ustilago_maydis_521 | 71022045 | 256 |
| Fungi | Vanderwaltozyma_polyspora_DSM_70294 | 156839776 | 234 |
| Fungi | Yarrowia_lipolytica_CLIB122 | 210075627 | 243 |
| Kinetoplastida | Leishmania_braziliensis_MHOM-BR-75-M2904 | 154331506 | 76.6 |
| Kinetoplastida | Leishmania_infantum_JPCM5 | 146075625 | 63.2 |
| Kinetoplastida | Leishmania_major_strain_Friedlin | 71746106 | 57 |
| Kinetoplastida | Trypanosoma_brucei_TREU927 | 84043554 | 145 |
| Kinetoplastida | Trypanosoma_cruzi_strain_CL_Brener | 71661326 | 164 |
| Metazoa | Acyrthosiphon_pisum | 193669292 | 259 |
| Metazoa | Aedes_aegypti | 157120564 | 306 |
| Metazoa | Anopheles_gambiae_str-_PEST | 58396352 | 306 |
| Metazoa | Apis_mellifera | 110761078 | 283 |
| Metazoa | Bombyx_mori | 114052038 | 288 |
| Metazoa | Brugia_malayi | 170583855 | 267 |
| Metazoa | Caenorhabditis_briggsae_AF16 | 157775075 | 276 |
| Metazoa | Caenorhabditis_elegans | 17552512 | 278 |
| Metazoa | Ciona_intestinalis | 74096479 | 300 |
| Metazoa | Danio_rerio | 45387521 | 336 |
| Metazoa | Drosophila_ananassae | 194760157 | 287 |
| Metazoa | Drosophila_grimshawi | 195028799 | 286 |
| Metazoa | Drosophila_melanogaster | 20129261 | 287 |
| Metazoa | Drosophila_pseudoobscura_pseudoobscura | 125986827 | 285 |
| Metazoa | Drosophila_virilis | 195382237 | 284 |
| Metazoa | Drosophila_willistoni | 195434655 | 289 |
| Metazoa | Equus_caballus | 194221092 | 337 |
| Metazoa | Gallus_gallus | 50754451 | 252 |
| Metazoa | Homo_sapiens | 5031595 | 341 |
| Metazoa | Nasonia_vitripennis | 156552388 | 281 |
| Metazoa | Nematostella_vectensis | 156398184 | 312 |
| Metazoa | Ornithorhynchus_anatinus | 149634124 | 337 |
| Metazoa | Strongylocentrotus_purpuratus | 115760314 | 311 |
| Metazoa | Tribolium_castaneum | 91085067 | 309 |
| Metazoa | Trichoplax_adhaerens | 196011964 | 263 |
| Metazoa | Xenopus_-Silurana-_tropicalis | 52345592 | 337 |
| Mycetozoa | Dictyostelium_discoideum_AX4 | 66826119 | 252 |
| Streptophyta | Arabidopsis_thaliana | 79325095 | 231 |
| Streptophyta | Oryza_sativa_Japonica_Group | 115456583 | 246 |
| Streptophyta | Physcomitrella_patens_subsp-_patens | 168047732 | 249 |
| Trichomonada | Trichomonas_vaginalis_G3 | 123437983 | 203 |
| ARPC5 | 66806101 | hit gi | bit score |
| Archamoebae | Entamoeba_dispar_SAW760 | 167376188 | 60.8 |
| Archamoebae | Entamoeba_histolytica_HM-1-IMSS | 67473307 | 59.7 |
| Fungi | Ashbya_gossypii_ATCC_10895 | 45187812 | 65.5 |
| Fungi | Botryotinia_fuckeliana_B05-10 | 154303365 | 49.3 |
| Fungi | Candida_albicans_SC5314 | 68487544 | 53.1 |
| Fungi | Candida_glabrata_CBS_138 | 50294958 | 62.4 |
| Fungi | Chaetomium_globosum_CBS_148-51 | 116200023 | 49.3 |
| Fungi | Coprinopsis_cinerea_okayama7-130 | 169854893 | 59.7 |
| Fungi | Cryptococcus_neoformans_var-_neoformans_JEC21 | 58258623 | 58.5 |
| Fungi | Debaryomyces_hansenii_CBS767 | 50424689 | 57.4 |
| Fungi | Gibberella_zeae_PH-1 | 46107878 | 50.4 |
| Fungi | Kluyveromyces_lactis_NRRL_Y-1140 | 50305193 | 48.9 |
| Fungi | Laccaria_bicolor_S238N-H82 | 170094034 | 51.6 |
| Fungi | Lodderomyces_elongisporus_NRRL_YB-4239 | 149238826 | 69.7 |
| Fungi | Magnaporthe_grisea_70-15 | 39961037 | 51.6 |
| Fungi | Malassezia_globosa_CBS_7966 | 164657474 | 78.2 |
| Fungi | Neurospora_crassa_OR74A | 85081453 | 44.7 |
| Fungi | Phaeosphaeria_nodorum_SN15 | 169622942 | 58.2 |
| Fungi | Pichia_guilliermondii_ATCC_6260 | 146423232 | 65.9 |
| Fungi | Pichia_stipitis_CBS_6054 | 126138770 | 55.5 |
| Fungi | Podospora_anserina_DSM_980 | 171689914 | 47 |
| Fungi | Saccharomyces_cerevisiae | 6322127 | 55.5 |
| Fungi | Schizosaccharomyces_japonicus_yFS275 | 213408903 | 60.5 |
| Fungi | Schizosaccharomyces_pombe | 19114639 | 54.3 |
| Fungi | Sclerotinia_sclerotiorum_1980 | 156054246 | 42.4 |
| Fungi | Ustilago_maydis_521 | 71012871 | 66.2 |
| Fungi | Vanderwaltozyma_polyspora_DSM_70294 | 156838445 | 58.2 |
| Fungi | Yarrowia_lipolytica_CLIB122 | 50552776 | 60.1 |
| Kinetoplastida | Trypanosoma_cruzi_strain_CL_Brener | 71405556 | 55.5 |
| Metazoa | Acyrthosiphon_pisum | 193664443 | 82.4 |
| Metazoa | Aedes_aegypti | 157120584 | 89 |
| Metazoa | Anopheles_gambiae_str-_PEST | 158299536 | 77 |
| Metazoa | Apis_mellifera | 110755080 | 70.9 |
| Metazoa | Bos_taurus | 78369476 | 102 |
| Metazoa | Brugia_malayi | 170574818 | 86.3 |
| Metazoa | Caenorhabditis_briggsae_AF16 | 157746395 | 82.4 |
| Metazoa | Caenorhabditis_elegans | 17508293 | 82.8 |
| Metazoa | Canis_lupus_familiaris | 73961151 | 97.1 |
| Metazoa | Ciona_intestinalis | 198435246 | 81.6 |
| Metazoa | Culex_quinquefasciatus | 170054104 | 75.1 |
| Metazoa | Danio_rerio | 192453542 | 101 |
| Metazoa | Drosophila_ananassae | 194770998 | 82.8 |
| Metazoa | Drosophila_erecta | 194854840 | 82.8 |
| Metazoa | Drosophila_grimshawi | 195032009 | 80.1 |
| Metazoa | Drosophila_melanogaster | 19920586 | 85.1 |
| Metazoa | Drosophila_mojavensis | 195117618 | 80.1 |
| Metazoa | Drosophila_persimilis | 195161372 | 79 |
| Metazoa | Drosophila_pseudoobscura_pseudoobscura | 125984442 | 80.9 |
| Metazoa | Drosophila_sechellia | 195342023 | 83.6 |
| Metazoa | Drosophila_virilis | 195386798 | 79.7 |
| Metazoa | Drosophila_willistoni | 195437284 | 80.5 |
| Metazoa | Equus_caballus | 194210400 | 102 |
| Metazoa | Gallus_gallus | 71896007 | 101 |
| Metazoa | Homo_sapiens | 5031593 | 101 |
| Metazoa | Macaca_mulatta | 109110241 | 86.7 |
| Metazoa | Monodelphis_domestica | 126306321 | 102 |
| Metazoa | Mus_musculus | 13385866 | 99.8 |
| Metazoa | Nematostella_vectensis | 156393364 | 90.5 |
| Metazoa | Ornithorhynchus_anatinus | 149636177 | 102 |
| Metazoa | Pan_troglodytes | 114568374 | 97.4 |
| Metazoa | Rattus_norvegicus | 109486016 | 90.1 |
| Metazoa | Strongylocentrotus_purpuratus | 115628791 | 44.3 |
| Metazoa | Sus_scrofa | 194033587 | 89 |
| Metazoa | Tribolium_castaneum | 91082167 | 79.7 |
| Metazoa | Trichoplax_adhaerens | 195999158 | 77 |
| Metazoa | Xenopus_-Silurana-_tropicalis | 62857795 | 95.9 |
| Metazoa | Xenopus_laevis | 147904174 | 94.4 |
| Mycetozoa | Dictyostelium_discoideum_AX4 | 66806101 | 282 |
| Streptophyta | Arabidopsis_thaliana | 18411708 | 57.8 |
| Streptophyta | Oryza_sativa_Japonica_Group | 115485903 | 62.8 |
| Streptophyta | Physcomitrella_patens_subsp-_patens | 168027395 | 65.9 |
| Trichomonada | Trichomonas_vaginalis_G3 | 123449146 | 45.8 |

| Actin-remodeling proteins: | | -e=0.1 |  |
| --- | --- | --- | --- |
| **WASp** | **query:10880935** | **hit gi** | **bit score** |
| Archamoebae | Entamoeba_dispar_SAW760 | 167377214 | 52.4 |
| Archamoebae | Entamoeba_histolytica_HM-1-IMSS | 67474254 | 53.1 |
| Choanoflagellida | Monosiga_brevicollis_MX1 | 167533287 | 39.3 |
| Ciliophora | Paramecium_tetraurelia_strain_d4-2 | 145510146 | 43.1 |
| Ciliophora | Tetrahymena_thermophila_SB210 | 118347675 | 74.3 |
| Fungi | Ajellomyces_capsulatus_NAm1 | 154280625 | 72.4 |
| Fungi | Ashbya_gossypii_ATCC_10895 | 45201381 | 81.6 |
| Fungi | Aspergillus_clavatus_NRRL_1 | 121704024 | 68.6 |
| Fungi | Aspergillus_fumigatus_Af293 | 70984635 | 53.9 |
| Fungi | Aspergillus_nidulans_FGSC_A4 | 67903456 | 55.1 |
| Fungi | Aspergillus_niger_CBS_513-88 | 145247190 | 70.9 |
| Fungi | Aspergillus_oryzae_RIB40 | 169778861 | 75.5 |
| Fungi | Aspergillus_terreus_NIH2624 | 115386684 | 73.9 |
| Fungi | Botryotinia_fuckeliana_B05-10 | 154291589 | 83.2 |
| Fungi | Candida_albicans_SC5314 | 68489733 | 85.5 |
| Fungi | Candida_glabrata_CBS_138 | 50288707 | 81.6 |
| Fungi | Chaetomium_globosum_CBS_148-51 | 116193573 | 80.1 |
| Fungi | Coccidioides_immitis_RS | 119181324 | 75.1 |
| Fungi | Coprinopsis_cinerea_okayama7-130 | 169856833 | 68.6 |
| Fungi | Cryptococcus_neoformans_var-_neoformans_B-3501A | 134112513 | 79 |
| Fungi | Cryptococcus_neoformans_var-_neoformans_JEC21 | 58267756 | 79 |
| Fungi | Debaryomyces_hansenii_CBS767 | 50421927 | 85.1 |
| Fungi | Gibberella_zeae_PH-1 | 46128557 | 85.5 |
| Fungi | Kluyveromyces_lactis_NRRL_Y-1140 | 50304913 | 72 |
| Fungi | Laccaria_bicolor_S238N-H82 | 170096694 | 81.3 |
| Fungi | Lodderomyces_elongisporus_NRRL_YB-4239 | 149235247 | 89 |
| Fungi | Magnaporthe_grisea_70-15 | 39970671 | 64.3 |
| Fungi | Malassezia_globosa_CBS_7966 | 164662383 | 62.8 |
| Fungi | Neosartorya_fischeri_NRRL_181 | 119467274 | 72.8 |
| Fungi | Neurospora_crassa_OR74A | 85114043 | 80.5 |
| Fungi | Penicillium_marneffei_ATCC_18224 | 212526706 | 66.2 |
| Fungi | Phaeosphaeria_nodorum_SN15 | 169609510 | 78.6 |
| Fungi | Pichia_guilliermondii_ATCC_6260 | 146417896 | 79.7 |
| Fungi | Pichia_stipitis_CBS_6054 | 150864617 | 87 |
| Fungi | Podospora_anserina_DSM_980 | 171684919 | 67.8 |
| Fungi | Pyrenophora_tritici-repentis_Pt-1C-BFP | 189198770 | 82 |
| Fungi | Saccharomyces_cerevisiae | 6324755 | 79.3 |
| Fungi | Schizosaccharomyces_japonicus_yFS275 | 213404408 | 82 |
| Fungi | Schizosaccharomyces_pombe | 19115670 | 76.6 |
| Fungi | Sclerotinia_sclerotiorum_1980 | 156056492 | 84.7 |
| Fungi | Vanderwaltozyma_polyspora_DSM_70294 | 156846323 | 71.2 |
| Metazoa | Acyrthosiphon_pisum | 193606145 | 92.8 |
| Metazoa | Aedes_aegypti | 157137962 | 60.5 |
| Metazoa | Anopheles_gambiae_str-_PEST | 158297785 | 55.1 |
| Metazoa | Apis_mellifera | 110768900 | 73.9 |
| Metazoa | Bos_taurus | 27806347 | 111 |
| Metazoa | Brugia_malayi | 170585133 | 56.2 |
| Metazoa | Caenorhabditis_briggsae_AF16 | 157757989 | 50.1 |
| Metazoa | Caenorhabditis_elegans | 71980678 | 45.4 |
| Metazoa | Canis_lupus_familiaris | 73975687 | 112 |
| Metazoa | Ciona_intestinalis | 198417714 | 120 |
| **WAVE/SCAR** | **query:66809177** | **hit gi** | **bit score** |
| Choanoflagellida | Monosiga_brevicollis_MX1 | 167534543 | 64.7 |
| Metazoa | Acyrthosiphon_pisum | 193579988 | 69.3 |
| Metazoa | Aedes_aegypti | 157132758 | 84.3 |
| Metazoa | Anopheles_gambiae_str-_PEST | 118788701 | 87.8 |
| Metazoa | Apis_mellifera | 48095266 | 79.3 |
| Metazoa | Bos_taurus | 76631519 | 96.3 |
| Metazoa | Brugia_malayi | 170595475 | 78.2 |
| Metazoa | Caenorhabditis_briggsae_AF16 | 157748317 | 74.7 |
| Metazoa | Caenorhabditis_elegans | 71989455 | 82 |
| Metazoa | Canis_lupus_familiaris | 73993386 | 97.1 |
| Metazoa | Ciona_intestinalis | 198413587 | 75.1 |
| Metazoa | Culex_quinquefasciatus | 170028297 | 85.5 |
| Metazoa | Danio_rerio | 123704810 | 118 |
| Metazoa | Drosophila_ananassae | 194762165 | 82 |
| Metazoa | Drosophila_erecta | 194861945 | 85.5 |
| Metazoa | Drosophila_grimshawi | 195030442 | 81.6 |
| Metazoa | Drosophila_melanogaster | 19921124 | 84 |
| Metazoa | Drosophila_mojavensis | 195118732 | 86.3 |
| Metazoa | Drosophila_persimilis | 195146676 | 82.4 |
| Metazoa | Drosophila_pseudoobscura_pseudoobscura | 198462015 | 82.4 |
| Metazoa | Drosophila_sechellia | 195340011 | 85.1 |
| Metazoa | Drosophila_simulans | 195578395 | 81.6 |
| Metazoa | Drosophila_virilis | 195384856 | 81.3 |
| Metazoa | Drosophila_willistoni | 195434088 | 81.3 |
| Metazoa | Drosophila_yakuba | 195472042 | 85.5 |
| Metazoa | Equus_caballus | 149730044 | 96.7 |
| Metazoa | Gallus_gallus | 118089593 | 105 |
| Metazoa | Homo_sapiens | 13699803 | 96.7 |
| Metazoa | Macaca_mulatta | 109120237 | 96.3 |
| Metazoa | Monodelphis_domestica | 126343832 | 101 |
| Metazoa | Mus_musculus | 21553113 | 95.5 |
| Metazoa | Nasonia_vitripennis | 156544367 | 79.7 |
| Metazoa | Nematostella_vectensis | 156362110 | 68.9 |
| Metazoa | Ornithorhynchus_anatinus | 149411038 | 102 |
| Metazoa | Pan_troglodytes | 114649082 | 96.3 |
| Metazoa | Pongo_abelii | 197098934 | 85.1 |
| Metazoa | Rattus_norvegicus | 68341973 | 85.1 |
| Metazoa | Strongylocentrotus_purpuratus | 115636725 | 84 |
| Metazoa | Trichoplax_adhaerens | 196002579 | 80.9 |
| Metazoa | Xenopus_-Silurana-_tropicalis | 118403872 | 98.2 |
| Metazoa | Xenopus_laevis | 148232782 | 84.3 |
| Mycetozoa | Dictyostelium_discoideum_AX4 | 66809177 | 901 |
| Streptophyta | Arabidopsis_thaliana | 79476972 | 71.6 |
| Streptophyta | Oryza_sativa_Japonica_Group | 115452489 | 60.5 |
| Streptophyta | Physcomitrella_patens_subsp-_patens | 168057329 | 82.4 |
| Trichomonada | Trichomonas_vaginalis_G3 | 123482536 | 58.5 |
| **Profilin** | **query:730406** | **hit gi** | **bit score** |
| Archamoebae | Entamoeba_dispar_SAW760 | 167384881 | 62.4 |
| Archamoebae | Entamoeba_histolytica_HM-1-IMSS | 67471313 | 65.5 |
| Chlorophyta | Chlamydomonas_reinhardtii | 159489468 | 73.9 |
| Chlorophyta | Ostreococcus_lucimarinus_CCE9901 | 145349887 | 37.7 |
| Choanoflagellida | Monosiga_brevicollis_MX1 | 167517653 | 94.4 |
| Fungi | Ajellomyces_capsulatus_NAm1 | 154272690 | 57.8 |
| Fungi | Ashbya_gossypii_ATCC_10895 | 45185520 | 138 |
| Fungi | Aspergillus_clavatus_NRRL_1 | 121714649 | 96.3 |
| Fungi | Aspergillus_fumigatus_Af293 | 70981925 | 110 |
| Fungi | Aspergillus_nidulans_FGSC_A4 | 67524053 | 87 |
| Fungi | Aspergillus_niger_CBS_513-88 | 145249208 | 130 |
| Fungi | Aspergillus_oryzae_RIB40 | 169763834 | 59.7 |
| Fungi | Aspergillus_terreus_NIH2624 | 115396702 | 105 |
| Fungi | Candida_albicans_SC5314 | 68476643 | 140 |
| Fungi | Candida_glabrata_CBS_138 | 50289739 | 142 |
| Fungi | Chaetomium_globosum_CBS_148-51 | 116196306 | 96.3 |
| Fungi | Coccidioides_immitis_RS | 119180545 | 123 |
| Fungi | Coprinopsis_cinerea_okayama7-130 | 169856917 | 102 |
| Fungi | Cryptococcus_neoformans_var-_neoformans_JEC21 | 58258955 | 99.8 |
| Fungi | Debaryomyces_hansenii_CBS767 | 50427143 | 149 |
| Fungi | Gibberella_zeae_PH-1 | 46124029 | 121 |
| Fungi | Kluyveromyces_lactis_NRRL_Y-1140 | 50308101 | 139 |
| Fungi | Laccaria_bicolor_S238N-H82 | 170096106 | 120 |
| Fungi | Lodderomyces_elongisporus_NRRL_YB-4239 | 149247148 | 146 |
| Fungi | Magnaporthe_grisea_70-15 | 145603318 | 79 |
| Fungi | Malassezia_globosa_CBS_7966 | 164661862 | 120 |
| Fungi | Neosartorya_fischeri_NRRL_181 | 119487423 | 101 |
| Fungi | Neurospora_crassa_OR74A | 85106701 | 136 |
| Fungi | Penicillium_marneffei_ATCC_18224 | 212545280 | 81.3 |
| Fungi | Phaeosphaeria_nodorum_SN15 | 169598552 | 100 |
| Fungi | Pichia_guilliermondii_ATCC_6260 | 146415464 | 146 |
| Fungi | Pichia_stipitis_CBS_6054 | 126133420 | 141 |
| Fungi | Podospora_anserina_DSM_980 | 171688662 | 71.2 |
| Fungi | Pyrenophora_tritici-repentis_Pt-1C-BFP | 189204396 | 107 |
| Fungi | Saccharomyces_cerevisiae | 6324696 | 147 |
| Fungi | Schizosaccharomyces_japonicus_yFS275 | 213410563 | 226 |
| Fungi | Schizosaccharomyces_pombe | 19114739 | 258 |
| Fungi | Ustilago_maydis_521 | 71018433 | 103 |
| Fungi | Vanderwaltozyma_polyspora_DSM_70294 | 156844227 | 150 |
| Fungi | Yarrowia_lipolytica_CLIB122 | 210075240 | 149 |
| Kinetoplastida | Leishmania_braziliensis_MHOM-BR-75-M2904 | 154342939 | 60.1 |
| Kinetoplastida | Leishmania_infantum_JPCM5 | 146096265 | 60.1 |
| Kinetoplastida | Leishmania_major_strain_Friedlin | 157873725 | 60.1 |
| Kinetoplastida | Trypanosoma_brucei_TREU927 | 74025736 | 50.8 |
| Kinetoplastida | Trypanosoma_cruzi_strain_CL_Brener | 71400168 | 46.2 |
| Metazoa | Acyrthosiphon_pisum | 193603639 | 96.7 |
| Metazoa | Aedes_aegypti | 157135572 | 99.8 |
| Metazoa | Anopheles_gambiae_str-_PEST | 158298819 | 101 |
| Metazoa | Apis_mellifera | 147902613 | 100 |
| Metazoa | Bombyx_mori | 112982865 | 94.4 |
| Metazoa | Bos_taurus | 115496077 | 71.6 |
| Metazoa | Brugia_malayi | 170581657 | 61.6 |
| Metazoa | Caenorhabditis_briggsae_AF16 | 157766821 | 80.1 |
| Metazoa | Caenorhabditis_elegans | 17568673 | 80.9 |
| Metazoa | Canis_lupus_familiaris | 73980614 | 65.1 |
| Metazoa | Ciona_intestinalis | 198427930 | 58.9 |
| Metazoa | Culex_quinquefasciatus | 170030306 | 100 |
| Metazoa | Danio_rerio | 56118638 | 39.7 |
| Metazoa | Drosophila_ananassae | 194761002 | 87.4 |
| Metazoa | Drosophila_erecta | 194856987 | 100 |
| Metazoa | Drosophila_grimshawi | 195030618 | 99 |
| Metazoa | Drosophila_melanogaster | 17136958 | 99.4 |
| Metazoa | Drosophila_persimilis | 195146544 | 76.3 |
| Metazoa | Drosophila_pseudoobscura_pseudoobscura | 125986885 | 99.8 |
| Metazoa | Drosophila_sechellia | 195342872 | 99 |
| Metazoa | Drosophila_simulans | 195576878 | 70.9 |
| Metazoa | Drosophila_virilis | 195385376 | 99 |
| Metazoa | Drosophila_willistoni | 195434731 | 97.8 |
| Metazoa | Equus_caballus | 149727732 | 65.5 |
| Metazoa | Gallus_gallus | 118089053 | 55.8 |
| Metazoa | Homo_sapiens | 40786418 | 76.3 |
| Metazoa | Macaca_mulatta | 109102180 | 73.2 |
| Metazoa | Monodelphis_domestica | 126343771 | 75.5 |
| Metazoa | Mus_musculus | 47058966 | 60.8 |
| Metazoa | Nasonia_vitripennis | 156542799 | 102 |
| Metazoa | Nematostella_vectensis | 156383727 | 89.4 |
| Metazoa | Ornithorhynchus_anatinus | 149408925 | 57 |
| Metazoa | Rattus_norvegicus | 57222328 | 59.3 |
| Metazoa | Strongylocentrotus_purpuratus | 47551153 | 57.8 |
| Metazoa | Tribolium_castaneum | 91082431 | 99.4 |
| Metazoa | Trichoplax_adhaerens | 195995597 | 86.7 |
| Metazoa | Xenopus_-Silurana-_tropicalis | 194332639 | 48.1 |
| Mycetozoa | Dictyostelium_discoideum_AX4 | 66808203 | 115 |
| Streptophyta | Arabidopsis_thaliana | 15224839 | 78.2 |
| Streptophyta | Oryza_sativa_Japonica_Group | 115466468 | 82 |
| Streptophyta | Physcomitrella_patens_subsp-_patens | 167999149 | 67 |
| Streptophyta | Zea_mays | 162461296 | 82 |
| Trichomonada | Trichomonas_vaginalis_G3 | 123446196 | 47 |
| Ciliophora | Paramecium_tetraurelia_strain_d4-2 | 145496342 |  |
| Ciliophora | Tetrahymena_thermophila_SB210 | 118358090 |  |
| Apicomplexa | Cryptosporidium_parvum_Iowa_II | 126644761 |  |
| Apicomplexa | Theileria_annulata_strain_Ankara | 84994870 |  |
| Apicomplexa | Babesia_bovis_T2Bo | 156085272 |  |
| Apicomplexa | Plasmodium_chabaudi_chabaudi | 70949341 |  |
| Apicomplexa | Plasmodium_vivax_SaI-1 | 156081740 |  |
| Apicomplexa | Theileria_parva_strain_Muguga | 71030962 |  |
| Apicomplexa | Plasmodium_yoelii_yoelii_str-_17XNL | 83317521 |  |
| Apicomplexa | Cryptosporidium_muris_RN66 | 209876311 |  |
| Apicomplexa | Plasmodium_berghei_str-_ANKA | 68069293 |  |
| Apicomplexa | Cryptosporidium_hominis_TU502 | 67593937 |  |
| Apicomplexa | Plasmodium_falciparum_3D7 | 124507183 |  |
| **Formin** | **query:158518557** | **hit gi** | **bit score** |
| Apicomplexa | Babesia_bovis_T2Bo | 156084964 | 77.4 |
| Apicomplexa | Cryptosporidium_hominis_TU502 | 67614162 | 77.8 |
| Apicomplexa | Cryptosporidium_muris_RN66 | 209882461 | 112 |
| Apicomplexa | Cryptosporidium_parvum_Iowa_II | 126649649 | 87 |
| Apicomplexa | Plasmodium_berghei_str-_ANKA | 68068739 | 70.9 |
| Apicomplexa | Plasmodium_chabaudi_chabaudi | 70951492 | 67.8 |
| Apicomplexa | Plasmodium_falciparum_3D7 | 124506531 | 72 |
| Apicomplexa | Plasmodium_vivax_SaI-1 | 156095234 | 73.2 |
| Apicomplexa | Plasmodium_yoelii_yoelii_str-_17XNL | 82915246 | 70.1 |
| Apicomplexa | Theileria_annulata_strain_Ankara | 84996973 | 88.6 |
| Apicomplexa | Theileria_parva_strain_Muguga | 71029098 | 76.3 |
| Archamoebae | Entamoeba_dispar_SAW760 | 167387772 | 95.1 |
| Archamoebae | Entamoeba_histolytica_HM-1-IMSS | 67471489 | 104 |
| Chlorophyta | Chlamydomonas_reinhardtii | 159466274 | 71.6 |
| Chlorophyta | Ostreococcus_lucimarinus_CCE9901 | 145347931 | 81.6 |
| Choanoflagellida | Monosiga_brevicollis_MX1 | 167517671 | 242 |
| Ciliophora | Paramecium_tetraurelia_strain_d4-2 | 145482449 | 77.4 |
| Ciliophora | Tetrahymena_thermophila_SB210 | 118349059 | 133 |
| Fungi | Ajellomyces_capsulatus_NAm1 | 154276796 | 43.9 |
| Fungi | Aspergillus_fumigatus_Af293 | 70984130 | 43.1 |
| Fungi | Aspergillus_nidulans_FGSC_A4 | 67540706 | 49.3 |
| Fungi | Aspergillus_niger_CBS_513-88 | 145250129 | 47 |
| Fungi | Aspergillus_terreus_NIH2624 | 115398544 | 46.2 |
| Fungi | Botryotinia_fuckeliana_B05-10 | 154300825 | 47.8 |
| Fungi | Candida_albicans_SC5314 | 68474640 | 58.5 |
| Fungi | Candida_glabrata_CBS_138 | 50291265 | 44.3 |
| Fungi | Chaetomium_globosum_CBS_148-51 | 116199253 | 43.1 |
| Fungi | Coprinopsis_cinerea_okayama7-130 | 169853769 | 57.4 |
| Fungi | Debaryomyces_hansenii_CBS767 | 50423049 | 48.9 |
| Fungi | Encephalitozoon_cuniculi_GB-M1 | 19074545 | 48.5 |
| Fungi | Kluyveromyces_lactis_NRRL_Y-1140 | 50304689 | 56.6 |
| Fungi | Lodderomyces_elongisporus_NRRL_YB-4239 | 149246125 | 48.1 |
| Fungi | Magnaporthe_grisea_70-15 | 145607193 | 50.4 |
| Fungi | Malassezia_globosa_CBS_7966 | 164655353 | 61.2 |
| Fungi | Neosartorya_fischeri_NRRL_181 | 119467790 | 42.4 |
| Fungi | Penicillium_marneffei_ATCC_18224 | 212546247 | 47 |
| Fungi | Phaeosphaeria_nodorum_SN15 | 169596791 | 48.5 |
| Fungi | Pyrenophora_tritici-repentis_Pt-1C-BFP | 189194826 | 46.6 |
| Fungi | Schizosaccharomyces_japonicus_yFS275 | 213401591 | 48.1 |
| Fungi | Ustilago_maydis_521 | 71005244 | 84 |
| Kinetoplastida | Leishmania_braziliensis_MHOM-BR-75-M2904 | 154335244 | 56.6 |
| Kinetoplastida | Leishmania_infantum_JPCM5 | 146083514 | 55.8 |
| Kinetoplastida | Leishmania_major_strain_Friedlin | 157867594 | 60.8 |
| Kinetoplastida | Trypanosoma_brucei_TREU927 | 72392841 | 69.3 |
| Kinetoplastida | Trypanosoma_cruzi_strain_CL_Brener | 71413468 | 66.6 |
| Metazoa | Acyrthosiphon_pisum | 193587140 | 205 |
| Metazoa | Aedes_aegypti | 157134894 | 275 |
| Metazoa | Anopheles_gambiae_str-_PEST | 158299240 | 259 |
| Metazoa | Apis_mellifera | 110756216 | 273 |
| Metazoa | Bos_taurus | 194670630 | 840 |
| Metazoa | Brugia_malayi | 170583375 | 78.2 |
| Metazoa | Caenorhabditis_briggsae_AF16 | 157776970 | 67.4 |
| Metazoa | Caenorhabditis_elegans | 32567078 | 97.1 |
| Metazoa | Canis_lupus_familiaris | 73999779 | 814 |
| Metazoa | Ciona_intestinalis | 198412143 | 210 |
| Metazoa | Culex_quinquefasciatus | 170033308 | 263 |
| Metazoa | Danio_rerio | 68395057 | 526 |
| Metazoa | Drosophila_ananassae | 194766453 | 226 |
| Metazoa | Drosophila_erecta | 194855853 | 267 |
| Metazoa | Drosophila_grimshawi | 195035239 | 253 |
| Metazoa | Drosophila_melanogaster | 24581562 | 258 |
| Metazoa | Drosophila_mojavensis | 195114396 | 258 |
| Metazoa | Drosophila_persimilis | 195147392 | 235 |
| Metazoa | Drosophila_pseudoobscura_pseudoobscura | 198473917 | 240 |
| Metazoa | Drosophila_sechellia | 195342372 | 259 |
| Metazoa | Drosophila_simulans | 195576454 | 255 |
| Metazoa | Drosophila_virilis | 195386454 | 255 |
| Metazoa | Drosophila_willistoni | 195433062 | 262 |
| Metazoa | Drosophila_yakuba | 195471185 | 259 |
| Metazoa | Equus_caballus | 194206836 | 856 |
| Metazoa | Gallus_gallus | 45383319 | 721 |
| Metazoa | Homo_sapiens | 157168329 | 843 |
| Metazoa | Macaca_mulatta | 109080487 | 773 |
| Metazoa | Monodelphis_domestica | 126277622 | 800 |
| Metazoa | Mus_musculus | 112807205 | 2989 |
| Metazoa | Nasonia_vitripennis | 156552774 | 246 |
| Metazoa | Nematostella_vectensis | 156397903 | 224 |
| Metazoa | Ornithorhynchus_anatinus | 149641685 | 449 |
| Metazoa | Pan_troglodytes | 114573465 | 425 |
| Metazoa | Pongo_abelii | 197100520 | 49.3 |
| Metazoa | Rattus_norvegicus | 109470558 | 872 |
| Metazoa | Strongylocentrotus_purpuratus | 115871982 | 345 |
| Metazoa | Sus_scrofa | 194034952 | 754 |
| Metazoa | Tribolium_castaneum | 91089831 | 297 |
| Metazoa | Trichoplax_adhaerens | 195995745 | 131 |
| Metazoa | Xenopus_-Silurana-_tropicalis | 194018630 | 644 |
| Metazoa | Xenopus_laevis | 147899302 | 83.6 |
| Mycetozoa | Dictyostelium_discoideum_AX4 | 111226792 | 137 |
| Streptophyta | Arabidopsis_thaliana | 30682092 | 98.6 |
| Streptophyta | Oryza_sativa_Japonica_Group | 115475690 | 107 |
| Streptophyta | Physcomitrella_patens_subsp-_patens | 168003259 | 86.7 |
| Streptophyta | Zea_mays | 212724028 | 68.9 |
| Trichomonada | Trichomonas_vaginalis_G3 | 154417633 | 81.6 |
| **Cofilin/ADF** | **query:3182971** | **hit gi** | **bit score** |
| Apicomplexa | Babesia_bovis_T2Bo | 156086730 | 57.8 |
| Apicomplexa | Cryptosporidium_hominis_TU502 | 67602060 | 84.3 |
| Apicomplexa | Cryptosporidium_muris_RN66 | 209875481 | 89.7 |
| Apicomplexa | Cryptosporidium_parvum_Iowa_II | 66358076 | 84 |
| Apicomplexa | Plasmodium_berghei_str-_ANKA | 68076629 | 74.7 |
| Apicomplexa | Plasmodium_chabaudi_chabaudi | 70945154 | 74.7 |
| Apicomplexa | Plasmodium_falciparum_3D7 | 124513938 | 82 |
| Apicomplexa | Plasmodium_vivax_SaI-1 | 156101407 | 86.3 |
| Apicomplexa | Plasmodium_yoelii_yoelii_str-_17XNL | 82913451 | 75.1 |
| Apicomplexa | Theileria_annulata_strain_Ankara | 84998256 | 55.1 |
| Apicomplexa | Theileria_parva_strain_Muguga | 71033611 | 57.4 |
| Archamoebae | Entamoeba_dispar_SAW760 | 167387758 | 129 |
| Archamoebae | Entamoeba_histolytica_HM-1-IMSS | 67471475 | 129 |
| Chlorophyta | Chlamydomonas_reinhardtii | 159468440 | 111 |
| Chlorophyta | Ostreococcus_lucimarinus_CCE9901 | 145345846 | 100 |
| Choanoflagellida | Monosiga_brevicollis_MX1 | 167524515 | 141 |
| Ciliophora | Paramecium_tetraurelia_strain_d4-2 | 145498978 | 94.4 |
| Ciliophora | Tetrahymena_thermophila_SB210 | 118375500 | 87.8 |
| Fungi | Ajellomyces_capsulatus_NAm1 | 154277020 | 95.1 |
| Fungi | Ashbya_gossypii_ATCC_10895 | 45188108 | 176 |
| Fungi | Aspergillus_clavatus_NRRL_1 | 121713472 | 96.3 |
| Fungi | Aspergillus_fumigatus_Af293 | 70997699 | 95.5 |
| Fungi | Aspergillus_nidulans_FGSC_A4 | 67523723 | 100 |
| Fungi | Aspergillus_niger_CBS_513-88 | 145249402 | 99.4 |
| Fungi | Aspergillus_oryzae_RIB40 | 169786007 | 112 |
| Fungi | Aspergillus_terreus_NIH2624 | 115401288 | 96.7 |
| Fungi | Botryotinia_fuckeliana_B05-10 | 154303542 | 58.5 |
| Fungi | Candida_albicans_SC5314 | 68482374 | 42.7 |
| Fungi | Candida_glabrata_CBS_138 | 50286867 | 169 |
| Fungi | Chaetomium_globosum_CBS_148-51 | 116204699 | 121 |
| Fungi | Coccidioides_immitis_RS | 119182505 | 104 |
| Fungi | Coprinopsis_cinerea_okayama7-130 | 169857933 | 154 |
| Fungi | Cryptococcus_neoformans_var-_neoformans_JEC21 | 58265648 | 171 |
| Fungi | Debaryomyces_hansenii_CBS767 | 50413644 | 183 |
| Fungi | Gibberella_zeae_PH-1 | 46123735 | 126 |
| Fungi | Kluyveromyces_lactis_NRRL_Y-1140 | 50307937 | 186 |
| Fungi | Laccaria_bicolor_S238N-H82 | 170088789 | 169 |
| Fungi | Lodderomyces_elongisporus_NRRL_YB-4239 | 149245114 | 41.2 |
| Fungi | Magnaporthe_grisea_70-15 | 39942168 | 143 |
| Fungi | Malassezia_globosa_CBS_7966 | 164656316 | 171 |
| Fungi | Neosartorya_fischeri_NRRL_181 | 119479205 | 97.1 |
| Fungi | Neurospora_crassa_OR74A | 164428037 | 126 |
| Fungi | Penicillium_marneffei_ATCC_18224 | 212538517 | 92.8 |
| Fungi | Phaeosphaeria_nodorum_SN15 | 169626375 | 51.2 |
| Fungi | Pichia_guilliermondii_ATCC_6260 | 146414590 | 40.4 |
| Fungi | Pichia_stipitis_CBS_6054 | 126139667 | 184 |
| Fungi | Podospora_anserina_DSM_980 | 171690290 | 132 |
| Fungi | Pyrenophora_tritici-repentis_Pt-1C-BFP | 189190434 | 128 |
| Fungi | Saccharomyces_cerevisiae | 6322978 | 180 |
| Fungi | Schizosaccharomyces_japonicus_yFS275 | 213406846 | 258 |
| Fungi | Schizosaccharomyces_pombe | 19115653 | 279 |
| Fungi | Sclerotinia_sclerotiorum_1980 | 156063944 | 109 |
| Fungi | Ustilago_maydis_521 | 71020461 | 177 |
| Fungi | Vanderwaltozyma_polyspora_DSM_70294 | 156839871 | 168 |
| Fungi | Yarrowia_lipolytica_CLIB122 | 50556548 | 192 |
| Kinetoplastida | Leishmania_braziliensis_MHOM-BR-75-M2904 | 154340880 | 69.3 |
| Kinetoplastida | Leishmania_infantum_JPCM5 | 146092921 | 62.4 |
| Kinetoplastida | Leishmania_major_strain_Friedlin | 73536570 | 60.8 |
| Kinetoplastida | Trypanosoma_brucei_TREU927 | 72387367 | 89.4 |
| Kinetoplastida | Trypanosoma_cruzi_strain_CL_Brener | 71417693 | 92.8 |
| Metazoa | Acyrthosiphon_pisum | 187179329 | 102 |
| Metazoa | Aedes_aegypti | 157103739 | 95.5 |
| Metazoa | Anopheles_gambiae_str-_PEST | 158300588 | 96.3 |
| Metazoa | Apis_mellifera | 110751158 | 86.7 |
| Metazoa | Bombyx_mori | 153792659 | 97.8 |
| Metazoa | Bos_taurus | 70778950 | 51.6 |
| Metazoa | Brugia_malayi | 170582273 | 84.3 |
| Metazoa | Caenorhabditis_elegans | 32566126 | 79.3 |
| Metazoa | Canis_lupus_familiaris | 73991481 | 93.2 |
| Metazoa | Ciona_intestinalis | 198423127 | 55.8 |
| Metazoa | Culex_quinquefasciatus | 170062807 | 56.6 |
| Metazoa | Danio_rerio | 45387807 | 90.5 |
| Metazoa | Drosophila_ananassae | 194769448 | 79 |
| Metazoa | Drosophila_erecta | 194892661 | 81.3 |
| Metazoa | Drosophila_grimshawi | 195028448 | 95.1 |
| Metazoa | Drosophila_melanogaster | 17136986 | 97.1 |
| Metazoa | Drosophila_mojavensis | 195122650 | 96.7 |
| Metazoa | Drosophila_persimilis | 195151275 | 97.1 |
| Metazoa | Drosophila_pseudoobscura_pseudoobscura | 198458041 | 97.4 |
| Metazoa | Drosophila_sechellia | 195345523 | 84.7 |
| Metazoa | Drosophila_simulans | 195570995 | 55.5 |
| Metazoa | Drosophila_virilis | 195382581 | 96.7 |
| Metazoa | Drosophila_willistoni | 195425594 | 98.2 |
| Metazoa | Drosophila_yakuba | 195481464 | 80.9 |
| Metazoa | Equus_caballus | 194224131 | 92.8 |
| Metazoa | Gallus_gallus | 52138701 | 89.7 |
| Metazoa | Homo_sapiens | 5031635 | 95.9 |
| Metazoa | Macaca_mulatta | 109105452 | 101 |
| Metazoa | Monodelphis_domestica | 126303672 | 92.8 |
| Metazoa | Mus_musculus | 6680924 | 95.9 |
| Metazoa | Nasonia_vitripennis | 156542763 | 86.3 |
| Metazoa | Nematostella_vectensis | 156375764 | 75.5 |
| Metazoa | Ornithorhynchus_anatinus | 149640975 | 92.8 |
| Metazoa | Pan_troglodytes | 114681108 | 92.8 |
| Metazoa | Pongo_abelii | 197102636 | 54.7 |
| Metazoa | Rattus_norvegicus | 109483879 | 96.7 |
| Metazoa | Strongylocentrotus_purpuratus | 115955758 | 53.1 |
| Metazoa | Sus_scrofa | 51592135 | 96.7 |
| Metazoa | Tribolium_castaneum | 91094039 | 100 |
| Metazoa | Trichoplax_adhaerens | 196007376 | 116 |
| Metazoa | Xenopus_-Silurana-_tropicalis | 58332014 | 90.5 |
| Metazoa | Xenopus_laevis | 147907080 | 90.9 |
| Mycetozoa | Dictyostelium_discoideum_AX4 | 66801703 | 113 |
| Streptophyta | Arabidopsis_thaliana | 18402587 | 119 |
| Streptophyta | Oryza_sativa_Japonica_Group | 115489680 | 129 |
| Streptophyta | Physcomitrella_patens_subsp-_patens | 168049547 | 120 |
| Streptophyta | Zea_mays | 164414398 | 114 |
| Trichomonada | Trichomonas_vaginalis_G3 | 154416500 | 110 |
| **Coronin** | **query:11023** | **hit gi** | **bit score** |
| Apicomplexa | Babesia_bovis_T2Bo | 156089181 | 152 |
| Apicomplexa | Cryptosporidium_hominis_TU502 | 67623271 | 249 |
| Apicomplexa | Cryptosporidium_muris_RN66 | 209879375 | 253 |
| Apicomplexa | Cryptosporidium_parvum_Iowa_II | 66363250 | 247 |
| Apicomplexa | Plasmodium_berghei_str-_ANKA | 68067036 | 192 |
| Apicomplexa | Plasmodium_chabaudi_chabaudi | 70951452 | 218 |
| Apicomplexa | Plasmodium_falciparum_3D7 | 124807051 | 228 |
| Apicomplexa | Plasmodium_vivax_SaI-1 | 156096005 | 204 |
| Apicomplexa | Plasmodium_yoelii_yoelii_str-_17XNL | 82915447 | 193 |
| Apicomplexa | Theileria_annulata_strain_Ankara | 84997369 | 148 |
| Archamoebae | Entamoeba_dispar_SAW760 | 167540270 | 490 |
| Archamoebae | Entamoeba_histolytica_HM-1-IMSS | 67477975 | 451 |
| Choanoflagellida | Monosiga_brevicollis_MX1 | 167518411 | 398 |
| Ciliophora | Paramecium_tetraurelia_strain_d4-2 | 145533751 | 254 |
| Ciliophora | Tetrahymena_thermophila_SB210 | 146164477 | 226 |
| Fungi | Ashbya_gossypii_ATCC_10895 | 45190720 | 347 |
| Fungi | Aspergillus_clavatus_NRRL_1 | 121716228 | 368 |
| Fungi | Aspergillus_fumigatus_Af293 | 71002158 | 362 |
| Fungi | Aspergillus_nidulans_FGSC_A4 | 67540342 | 349 |
| Fungi | Aspergillus_niger_CBS_513-88 | 145231566 | 347 |
| Fungi | Aspergillus_oryzae_RIB40 | 169772441 | 345 |
| Fungi | Aspergillus_terreus_NIH2624 | 115383760 | 362 |
| Fungi | Botryotinia_fuckeliana_B05-10 | 154301425 | 340 |
| Fungi | Candida_albicans_SC5314 | 77022724 | 361 |
| Fungi | Candida_glabrata_CBS_138 | 50290965 | 335 |
| Fungi | Chaetomium_globosum_CBS_148-51 | 116191745 | 351 |
| Fungi | Coccidioides_immitis_RS | 119190903 | 354 |
| Fungi | Coprinopsis_cinerea_okayama7-130 | 169848889 | 336 |
| Fungi | Cryptococcus_neoformans_var-_neoformans_JEC21 | 58270688 | 337 |
| Fungi | Debaryomyces_hansenii_CBS767 | 50420725 | 359 |
| Fungi | Encephalitozoon_cuniculi_GB-M1 | 19173438 | 40 |
| Fungi | Gibberella_zeae_PH-1 | 46125867 | 352 |
| Fungi | Kluyveromyces_lactis_NRRL_Y-1140 | 50306415 | 349 |
| Fungi | Laccaria_bicolor_S238N-H82 | 170101386 | 326 |
| Fungi | Lodderomyces_elongisporus_NRRL_YB-4239 | 149248244 | 47.8 |
| Fungi | Magnaporthe_grisea_70-15 | 145608772 | 355 |
| Fungi | Malassezia_globosa_CBS_7966 | 164658898 | 306 |
| Fungi | Neosartorya_fischeri_NRRL_181 | 119481709 | 368 |
| Fungi | Neurospora_crassa_OR74A | 85080705 | 341 |
| Fungi | Penicillium_marneffei_ATCC_18224 | 212527452 | 358 |
| Fungi | Phaeosphaeria_nodorum_SN15 | 169619507 | 360 |
| Fungi | Podospora_anserina_DSM_980 | 171687090 | 329 |
| Fungi | Pyrenophora_tritici-repentis_Pt-1C-BFP | 189206946 | 366 |
| Fungi | Saccharomyces_cerevisiae | 6323460 | 363 |
| Fungi | Schizosaccharomyces_japonicus_yFS275 | 213402351 | 360 |
| Fungi | Schizosaccharomyces_pombe | 19114087 | 349 |
| Fungi | Sclerotinia_sclerotiorum_1980 | 156033039 | 352 |
| Fungi | Ustilago_maydis_521 | 71020667 | 338 |
| Fungi | Vanderwaltozyma_polyspora_DSM_70294 | 156841188 | 340 |
| Fungi | Yarrowia_lipolytica_CLIB122 | 50545411 | 353 |
| Kinetoplastida | Leishmania_braziliensis_MHOM-BR-75-M2904 | 154337944 | 258 |
| Kinetoplastida | Leishmania_infantum_JPCM5 | 146087418 | 262 |
| Kinetoplastida | Leishmania_major_strain_Friedlin | 157869818 | 266 |
| Kinetoplastida | Trypanosoma_brucei_TREU927 | 72392681 | 271 |
| Kinetoplastida | Trypanosoma_cruzi_strain_CL_Brener | 71424836 | 296 |
| Metazoa | Acyrthosiphon_pisum | 193594266 | 371 |
| Metazoa | Aedes_aegypti | 157118304 | 380 |
| Metazoa | Anopheles_gambiae_str-_PEST | 158294944 | 382 |
| Metazoa | Apis_mellifera | 66507360 | 384 |
| Metazoa | Bos_taurus | 125991864 | 387 |
| Metazoa | Brugia_malayi | 170591296 | 342 |
| Metazoa | Caenorhabditis_briggsae_AF16 | 157777316 | 142 |
| Metazoa | Caenorhabditis_elegans | 25150738 | 334 |
| Metazoa | Canis_lupus_familiaris | 73995354 | 386 |
| Metazoa | Ciona_intestinalis | 198417588 | 247 |
| Metazoa | Culex_quinquefasciatus | 170058296 | 339 |
| Metazoa | Danio_rerio | 41055464 | 396 |
| Metazoa | Drosophila_ananassae | 194755415 | 375 |
| Metazoa | Drosophila_erecta | 194863990 | 379 |
| Metazoa | Drosophila_grimshawi | 195029501 | 380 |
| Metazoa | Drosophila_melanogaster | 24586098 | 379 |
| Metazoa | Drosophila_mojavensis | 195119438 | 372 |
| Metazoa | Drosophila_persimilis | 195149175 | 378 |
| Metazoa | Drosophila_pseudoobscura_pseudoobscura | 198471316 | 251 |
| Metazoa | Drosophila_sechellia | 195356947 | 380 |
| Metazoa | Drosophila_virilis | 195401595 | 378 |
| Metazoa | Drosophila_willistoni | 195435441 | 378 |
| Metazoa | Drosophila_yakuba | 195474215 | 377 |
| Metazoa | Equus_caballus | 149724132 | 383 |
| Metazoa | Gallus_gallus | 86129440 | 383 |
| Metazoa | Homo_sapiens | 7656991 | 387 |
| Metazoa | Macaca_mulatta | 109113840 | 382 |
| Metazoa | Monodelphis_domestica | 126324331 | 389 |
| Metazoa | Mus_musculus | 31542413 | 384 |
| Metazoa | Nasonia_vitripennis | 156550141 | 393 |
| Metazoa | Nematostella_vectensis | 156385222 | 336 |
| Metazoa | Ornithorhynchus_anatinus | 149634274 | 379 |
| Metazoa | Oryctolagus_cuniculus | 130487941 | 374 |
| Metazoa | Pan_troglodytes | 114646788 | 386 |
| Metazoa | Pongo_abelii | 197100107 | 376 |
| Metazoa | Rattus_norvegicus | 157818697 | 385 |
| Metazoa | Strongylocentrotus_purpuratus | 72008049 | 331 |
| Metazoa | Sus_scrofa | 194043123 | 387 |
| Metazoa | Tribolium_castaneum | 189241935 | 388 |
| Metazoa | Trichoplax_adhaerens | 196011976 | 382 |
| Metazoa | Xenopus_-Silurana-_tropicalis | 54262238 | 390 |
| Metazoa | Xenopus_laevis | 148225981 | 386 |
| Mycetozoa | Dictyostelium_discoideum_AX4 | 66827977 | 918 |
| Trichomonada | Trichomonas_vaginalis_G3 | 123398376 | 256 |
| **alpha-actinin** | **query:11023** | **hit gi** | **bit score** |
| Apicomplexa | Babesia_bovis_T2Bo | 156083146 | 71.2 |
| Apicomplexa | Cryptosporidium_hominis_TU502 | 67621647 | 82 |
| Apicomplexa | Cryptosporidium_muris_RN66 | 209878748 | 81.6 |
| Apicomplexa | Cryptosporidium_parvum_Iowa_II | 66359714 | 55.5 |
| Apicomplexa | Plasmodium_berghei_str-_ANKA | 68066708 | 83.2 |
| Apicomplexa | Plasmodium_chabaudi_chabaudi | 70931007 | 67.8 |
| Apicomplexa | Plasmodium_falciparum_3D7 | 124809127 | 84.3 |
| Apicomplexa | Plasmodium_vivax_SaI-1 | 156101878 | 84.7 |
| Apicomplexa | Plasmodium_yoelii_yoelii_str-_17XNL | 83317739 | 46.6 |
| Apicomplexa | Theileria_annulata_strain_Ankara | 84994554 | 70.1 |
| Apicomplexa | Theileria_parva_strain_Muguga | 71031284 | 70.5 |
| Archamoebae | Entamoeba_dispar_SAW760 | 167384828 | 312 |
| Archamoebae | Entamoeba_histolytica_HM-1-IMSS | 67475126 | 313 |
| Chlorophyta | Chlamydomonas_reinhardtii | 159490918 | 82.8 |
| Chlorophyta | Ostreococcus_lucimarinus_CCE9901 | 145341622 | 82.4 |
| Choanoflagellida | Monosiga_brevicollis_MX1 | 167524491 | 520 |
| Chroococcales | Crocosphaera_watsonii_WH_8501 | 67923756 | 44.7 |
| Chroococcales | Cyanothece_sp-_ATCC_51142 | 172036759 | 47.4 |
| Chroococcales | Cyanothece_sp-_CCY_0110 | 126657161 | 48.1 |
| Chroococcales | Cyanothece_sp-_PCC_7424 | 186899153 | 42.7 |
| Ciliophora | Paramecium_tetraurelia_strain_d4-2 | 145525082 | 79.7 |
| Ciliophora | Tetrahymena_thermophila_SB210 | 146181449 | 82 |
| Diplomonadida | Giardia_lamblia_ATCC_50803 | 159111176 | 79.7 |
| Fungi | Ajellomyces_capsulatus_NAm1 | 154285800 | 278 |
| Fungi | Ashbya_gossypii_ATCC_10895 | 45187535 | 66.2 |
| Fungi | Aspergillus_clavatus_NRRL_1 | 121713218 | 278 |
| Fungi | Aspergillus_fumigatus_Af293 | 70998154 | 276 |
| Fungi | Aspergillus_nidulans_FGSC_A4 | 67901440 | 257 |
| Fungi | Aspergillus_niger_CBS_513-88 | 145235379 | 267 |
| Fungi | Aspergillus_oryzae_RIB40 | 169778169 | 267 |
| Fungi | Aspergillus_terreus_NIH2624 | 115433512 | 271 |
| Fungi | Botryotinia_fuckeliana_B05-10 | 154323103 | 293 |
| Fungi | Candida_albicans_SC5314 | 68488571 | 69.3 |
| Fungi | Candida_glabrata_CBS_138 | 50286099 | 67.4 |
| Fungi | Chaetomium_globosum_CBS_148-51 | 116191933 | 298 |
| Fungi | Coccidioides_immitis_RS | 119183879 | 300 |
| Fungi | Coprinopsis_cinerea_okayama7-130 | 169857933 | 271 |
| Fungi | Cryptococcus_neoformans_var-_neoformans_B-3501A | 134109363 | 41.6 |
| Fungi | Cryptococcus_neoformans_var-_neoformans_JEC21 | 58265646 | 284 |
| Fungi | Debaryomyces_hansenii_CBS767 | 50418911 | 71.6 |
| Fungi | Encephalitozoon_cuniculi_GB-M1 | 85014171 | 209 |
| Fungi | Gibberella_zeae_PH-1 | 46125813 | 294 |
| Fungi | Kluyveromyces_lactis_NRRL_Y-1140 | 50303999 | 65.1 |
| Fungi | Laccaria_bicolor_S238N-H82 | 170088787 | 291 |
| Fungi | Lodderomyces_elongisporus_NRRL_YB-4239 | 149236605 | 54.7 |
| Fungi | Magnaporthe_grisea_70-15 | 145608660 | 272 |
| Fungi | Malassezia_globosa_CBS_7966 | 164658221 | 66.2 |
| Fungi | Neosartorya_fischeri_NRRL_181 | 119479651 | 277 |
| Fungi | Neurospora_crassa_OR74A | 85084500 | 299 |
| Fungi | Penicillium_marneffei_ATCC_18224 | 212535438 | 302 |
| Fungi | Phaeosphaeria_nodorum_SN15 | 169612279 | 284 |
| Fungi | Pichia_guilliermondii_ATCC_6260 | 146412714 | 79.7 |
| Fungi | Pichia_stipitis_CBS_6054 | 150951203 | 73.6 |
| Fungi | Podospora_anserina_DSM_980 | 171686902 | 299 |
| Fungi | Pyrenophora_tritici-repentis_Pt-1C-BFP | 189189950 | 295 |
| Fungi | Saccharomyces_cerevisiae | 6319585 | 69.7 |
| Fungi | Schizosaccharomyces_japonicus_yFS275 | 213409880 | 288 |
| Fungi | Schizosaccharomyces_pombe | 19115207 | 279 |
| Fungi | Sclerotinia_sclerotiorum_1980 | 156064517 | 291 |
| Fungi | Ustilago_maydis_521 | 71020457 | 310 |
| Fungi | Vanderwaltozyma_polyspora_DSM_70294 | 156837678 | 65.5 |
| Fungi | Yarrowia_lipolytica_CLIB122 | 50555435 | 244 |
| Kinetoplastida | Leishmania_braziliensis_MHOM-BR-75-M2904 | 154340319 | 43.1 |
| Kinetoplastida | Leishmania_infantum_JPCM5 | 146103804 | 47.4 |
| Kinetoplastida | Leishmania_major_strain_Friedlin | 157877241 | 47.8 |
| Kinetoplastida | Trypanosoma_brucei_TREU927 | 74025586 | 81.3 |
| Kinetoplastida | Trypanosoma_cruzi_strain_CL_Brener | 71411702 | 82 |
| Metazoa | Acyrthosiphon_pisum | 193683742 | 590 |
| Metazoa | Aedes_aegypti | 157115648 | 602 |
| Metazoa | Anopheles_gambiae_str-_PEST | 58396045 | 586 |
| Metazoa | Apis_mellifera | 110761364 | 559 |
| Metazoa | Bombyx_mori | 114051846 | 53.5 |
| Metazoa | Bos_taurus | 77736221 | 600 |
| Metazoa | Brugia_malayi | 170594019 | 529 |
| Metazoa | Caenorhabditis_briggsae_AF16 | 157762340 | 575 |
| Metazoa | Caenorhabditis_elegans | 17565034 | 579 |
| Metazoa | Canis_lupus_familiaris | 73952476 | 608 |
| Metazoa | Ciona_intestinalis | 198426722 | 576 |
| Metazoa | Culex_quinquefasciatus | 170038033 | 327 |
| Metazoa | Danio_rerio | 83025080 | 598 |
| Metazoa | Drosophila_ananassae | 194768725 | 579 |
| Metazoa | Drosophila_erecta | 194912870 | 578 |
| Metazoa | Drosophila_grimshawi | 195046036 | 580 |
| Metazoa | Drosophila_melanogaster | 24639238 | 590 |
| Metazoa | Drosophila_mojavensis | 195130251 | 579 |
| Metazoa | Drosophila_persimilis | 195169481 | 405 |
| Metazoa | Drosophila_pseudoobscura_pseudoobscura | 198470594 | 581 |
| Metazoa | Drosophila_sechellia | 195351770 | 329 |
| Metazoa | Drosophila_simulans | 195567371 | 329 |
| Metazoa | Drosophila_virilis | 195397485 | 588 |
| Metazoa | Drosophila_willistoni | 195447732 | 571 |
| Metazoa | Drosophila_yakuba | 195477858 | 557 |
| Metazoa | Equus_caballus | 194225130 | 592 |
| Metazoa | Gallus_gallus | 46048687 | 601 |
| Metazoa | Homo_sapiens | 4501893 | 600 |
| Metazoa | Macaca_mulatta | 109019881 | 601 |
| Metazoa | Monodelphis_domestica | 126306976 | 603 |
| Metazoa | Mus_musculus | 157951643 | 599 |
| Metazoa | Nasonia_vitripennis | 156544798 | 328 |
| Metazoa | Nematostella_vectensis | 156384739 | 604 |
| Metazoa | Oncorhynchus_mykiss | 185134996 | 50.1 |
| Metazoa | Ornithorhynchus_anatinus | 149607640 | 340 |
| Metazoa | Oryctolagus_cuniculus | 130505621 | 45.4 |
| Metazoa | Ovis_aries | 163914421 | 60.1 |
| Metazoa | Pan_troglodytes | 114573366 | 584 |
| Metazoa | Pongo_abelii | 197099422 | 585 |
| Metazoa | Rattus_norvegicus | 13591902 | 597 |
| Metazoa | Salmo_salar | 213512940 | 51.2 |
| Metazoa | Strongylocentrotus_purpuratus | 72018985 | 592 |
| Metazoa | Sus_scrofa | 194042529 | 601 |
| Metazoa | Takifugu_rubripes | 118344604 | 105 |
| Metazoa | Tribolium_castaneum | 91080533 | 588 |
| Metazoa | Trichoplax_adhaerens | 196006742 | 574 |
| Metazoa | Xenopus_-Silurana-_tropicalis | 52346020 | 593 |
| Metazoa | Xenopus_laevis | 148234146 | 598 |
| Mycetozoa | Dictyostelium_discoideum_AX4 | 66827249 | 1767 |
| Streptophyta | Arabidopsis_thaliana | 15219652 | 89 |
| Streptophyta | Oryza_sativa_Japonica_Group | 115464615 | 87.8 |
| Streptophyta | Physcomitrella_patens_subsp-_patens | 168021054 | 90.5 |
| Streptophyta | Zea_mays | 212722842 | 88.2 |
| Trichomonada | Trichomonas_vaginalis_G3 | 154417044 | 244 |
| **filamin** | **query:121115** | **hit gi** | **bit score** |
| Archamoebae | Entamoeba_dispar_SAW760 | 167395554 | 330 |
| Archamoebae | Entamoeba_histolytica_HM-1-IMSS | 67484090 | 331 |
| Metazoa | Acyrthosiphon_pisum | 193594163 | 213 |
| Metazoa | Aedes_aegypti | 157123651 | 199 |
| Metazoa | Anopheles_gambiae_str-_PEST | 158292049 | 266 |
| Metazoa | Apis_mellifera | 110757964 | 158 |
| Metazoa | Bos_taurus | 194666432 | 303 |
| Metazoa | Brugia_malayi | 170578963 | 187 |
| Metazoa | Caenorhabditis_briggsae_AF16 | 157759271 | 184 |
| Metazoa | Caenorhabditis_elegans | 17543920 | 202 |
| Metazoa | Canis_lupus_familiaris | 74008811 | 304 |
| Metazoa | Ciona_intestinalis | 198422452 | 216 |
| Metazoa | Culex_quinquefasciatus | 170030696 | 247 |
| Metazoa | Danio_rerio | 189535920 | 296 |
| Metazoa | Drosophila_ananassae | 194742303 | 270 |
| Metazoa | Drosophila_erecta | 194900506 | 278 |
| Metazoa | Drosophila_grimshawi | 195061451 | 280 |
| Metazoa | Drosophila_melanogaster | 62484180 | 276 |
| Metazoa | Drosophila_mojavensis | 195109448 | 267 |
| Metazoa | Drosophila_persimilis | 195165936 | 288 |
| Metazoa | Drosophila_pseudoobscura_pseudoobscura | 198450410 | 270 |
| Metazoa | Drosophila_sechellia | 195349191 | 277 |
| Metazoa | Drosophila_simulans | 195570217 | 275 |
| Metazoa | Drosophila_virilis | 195394794 | 274 |
| Metazoa | Drosophila_willistoni | 195446250 | 270 |
| Metazoa | Drosophila_yakuba | 195500100 | 276 |
| Metazoa | Equus_caballus | 194228448 | 303 |
| Metazoa | Gallus_gallus | 45383035 | 296 |
| Metazoa | Homo_sapiens | 160420317 | 306 |
| Metazoa | Macaca_mulatta | 109132798 | 302 |
| Metazoa | Monodelphis_domestica | 126336171 | 273 |
| Metazoa | Mus_musculus | 125347376 | 306 |
| Metazoa | Nasonia_vitripennis | 156548214 | 284 |
| Metazoa | Nematostella_vectensis | 156400860 | 274 |
| Metazoa | Ornithorhynchus_anatinus | 149637293 | 310 |
| Metazoa | Pan_troglodytes | 114587549 | 291 |
| Metazoa | Pongo_abelii | 197099422 | 165 |
| Metazoa | Rattus_norvegicus | 109462323 | 305 |
| Metazoa | Strongylocentrotus_purpuratus | 115774591 | 283 |
| Metazoa | Sus_scrofa | 194042529 | 164 |
| Metazoa | Takifugu_rubripes | 118344604 | 47.8 |
| Metazoa | Tribolium_castaneum | 91077512 | 161 |
| Metazoa | Trichoplax_adhaerens | 196016379 | 223 |
| Metazoa | Xenopus_-Silurana-_tropicalis | 52346020 | 168 |
| Metazoa | Xenopus_laevis | 147898867 | 169 |
| Mycetozoa | Dictyostelium_discoideum_AX4 | 66826629 | 1742 |

| **some WAVE/SCAR proteins:** | |  |  |  |  |  |  |  |  |  |
| --- | --- | --- | --- | --- | --- | --- | --- | --- | --- | --- |
|  |  |  |  |  |  |  |  |  |  |  |
| 109000020 | PREDICTED: similar to WAS protein family, member 2 [Macaca mulatta] | | | | | | |  |  |  |
| 109073063 | PREDICTED: WAS protein family, member 1 [Macaca mulatta] | | | | | |  |  |  |  |
| 109120237 | PREDICTED: similar to WAS protein family, member 3 isoform 2 [Macaca mulatta] | | | | | | | |  |  |
| 109120245 | PREDICTED: similar to WAS protein family, member 3 isoform 4 [Macaca mulatta] | | | | | | | |  |  |
| 109496483 | PREDICTED: similar to WAS protein family, member 3 [Rattus norvegicus] | | | | | | | |  |  |
| 11386183 | WAS protein family, member 2 [Homo sapiens] | | | | |  |  |  |  |  |
| 114555015 | PREDICTED: WAS protein family, member 2 isoform 1 [Pan troglodytes] | | | | | | |  |  |  |
| 114609012 | PREDICTED: similar to Wiskott-Aldrich syndrome protein family member 1 (WASP-family protein member 1) (Protein WAVE-1) (Verprolin homology domain-containing protein 1) [Pan troglodytes] | | | | | | | | | |
| 114649082 | PREDICTED: WAS protein family, member 3 isoform 8 [Pan troglodytes] | | | | | | |  |  |  |
| 114649096 | PREDICTED: WAS protein family, member 3 isoform 6 [Pan troglodytes] | | | | | | |  |  |  |
| 115452489 | Os03g0298700 [Oryza sativa (japonica cultivar-group)] | | | | | |  |  |  |  |
| 115456193 | Os03g0816900 [Oryza sativa (japonica cultivar-group)] | | | | | |  |  |  |  |
| 115511054 | WAS protein family, member 1 [Bos taurus] | | | | |  |  |  |  |  |
| 115636725 | PREDICTED: similar to ENSANGP00000006560 [Strongylocentrotus purpuratus] | | | | | | | |  |  |
| 118084989 | PREDICTED: similar to WAS protein family, member 3 [Gallus gallus] | | | | | | |  |  |  |
| 118089593 | PREDICTED: similar to WAS protein family, member 3 [Gallus gallus] | | | | | | |  |  |  |
| 118118274 | PREDICTED: similar to WASP-family protein [Gallus gallus] | | | | | |  |  |  |  |
| 118403872 | WAS protein family, member 3 [Xenopus tropicalis] | | | | | |  |  |  |  |
| 118788701 | AGAP008518-PA [Anopheles gambiae str. PEST] | | | | |  |  |  |  |  |
| 123391466 | hypothetical protein TVAG_078700 [Trichomonas vaginalis G3] | | | | | | |  |  |  |
| 123482536 | WH2 motif family protein [Trichomonas vaginalis G3] | | | | | |  |  |  |  |
| 123502252 | conserved hypothetical protein [Trichomonas vaginalis G3] | | | | | |  |  |  |  |
| 123508796 | conserved hypothetical protein [Trichomonas vaginalis G3] | | | | | |  |  |  |  |
| 123704810 | hypothetical protein LOC563632 [Danio rerio] | | | | |  |  |  |  |  |
| 125630645 | WAS protein family, member 2 [Bos taurus] | | | | |  |  |  |  |  |
| 125817664 | PREDICTED: hypothetical protein [Danio rerio] | | | | |  |  |  |  |  |
| 125820875 | PREDICTED: similar to WAS protein family, member 3 [Danio rerio] | | | | | | |  |  |  |
| 125847108 | PREDICTED: hypothetical protein [Danio rerio] | | | | |  |  |  |  |  |
| 126002276 | GA18320-PA [Drosophila pseudoobscura] | | | | |  |  |  |  |  |
| 126327399 | PREDICTED: similar to WAS protein family, member 3 [Monodelphis domestica] | | | | | | | |  |  |
| 126328707 | PREDICTED: similar to reverse transcriptase [Monodelphis domestica] | | | | | | |  |  |  |
| 126343830 | PREDICTED: similar to WAS protein family, member 3 isoform 1 [Monodelphis domestica] | | | | | | | | |  |
| 126343832 | PREDICTED: similar to WAS protein family, member 3 isoform 2 [Monodelphis domestica] | | | | | | | | |  |
| 13699803 | WAS protein family, member 3 [Homo sapiens] | | | | |  |  |  |  |  |
| 13994209 | WASP family 1 [Mus musculus] | | | |  |  |  |  |  |  |
| 148232782 | MGC84671 protein [Xenopus laevis] | | | |  |  |  |  |  |  |
| 149411038 | PREDICTED: similar to WAS protein family, member 3 isoform 1 [Ornithorhynchus anatinus] | | | | | | | | |  |
| 149411040 | PREDICTED: similar to WAS protein family, member 3 isoform 2 [Ornithorhynchus anatinus] | | | | | | | | |  |
| 149598958 | PREDICTED: similar to WASF3 protein [Ornithorhynchus anatinus] | | | | | | |  |  |  |
| 149638852 | PREDICTED: similar to WAS protein family, member 1 [Ornithorhynchus anatinus] | | | | | | | |  |  |
| 149694187 | PREDICTED: similar to SWI/SNF related, matrix associated, actin dependent regulator of chromatin, subfamily a, member 1 [Equus caballus] | | | | | | | | | |
| 149722881 | PREDICTED: similar to WAS protein family, member 1 [Equus caballus] | | | | | | |  |  |  |
| 149730044 | PREDICTED: similar to WAS protein family, member 3 [Equus caballus] | | | | | | |  |  |  |
| 154415943 | conserved hypothetical protein [Trichomonas vaginalis G3] | | | | | |  |  |  |  |
| 156362110 | predicted protein [Nematostella vectensis] | | | | |  |  |  |  |  |
| 156544367 | PREDICTED: similar to actin binding protein, putative [Nasonia vitripennis] | | | | | | | |  |  |
| 156717354 | WAS protein family, member 1 [Xenopus tropicalis] | | | | | |  |  |  |  |
| 156717788 | hypothetical protein LOC100125044 [Xenopus tropicalis] | | | | | |  |  |  |  |
| 156717910 | hypothetical protein LOC100125121 [Xenopus tropicalis] | | | | | |  |  |  |  |
| 157132758 | actin binding protein, putative [Aedes aegypti] | | | | |  |  |  |  |  |
| 157748317 | Hypothetical protein CBG18687 [Caenorhabditis briggsae AF16] | | | | | | |  |  |  |
| 167534543 | predicted protein [Monosiga brevicollis MX1] | | | | |  |  |  |  |  |
| 168002754 | predicted protein [Physcomitrella patens subsp. patens] | | | | | |  |  |  |  |
| 168007496 | predicted protein [Physcomitrella patens subsp. patens] | | | | | |  |  |  |  |
| 168042085 | predicted protein [Physcomitrella patens subsp. patens] | | | | | |  |  |  |  |
| 168051887 | predicted protein [Physcomitrella patens subsp. patens] | | | | | |  |  |  |  |
| 168055907 | predicted protein [Physcomitrella patens subsp. patens] | | | | | |  |  |  |  |
| 168057329 | predicted protein [Physcomitrella patens subsp. patens] | | | | | |  |  |  |  |
| 168059809 | predicted protein [Physcomitrella patens subsp. patens] | | | | | |  |  |  |  |
| 170028297 | actin binding protein [Culex pipiens quinquefasciatus] | | | | | |  |  |  |  |
| 170582910 | hypothetical protein Bm1_24430 [Brugia malayi] | | | | |  |  |  |  |  |
| 170595475 | WH2 motif family protein [Brugia malayi] | | | | |  |  |  |  |  |
| 186479003 | WAVE2 (WASP (WISKOTT-ALDRICH SYNDROME PROTEIN)-FAMILY VERPROLIN HOMOLOGOUS PROTEIN 2) [Arabidopsis thaliana] | | | | | | | | | |
| 186479005 | WAVE2 (WASP (WISKOTT-ALDRICH SYNDROME PROTEIN)-FAMILY VERPROLIN HOMOLOGOUS PROTEIN 2) [Arabidopsis thaliana] | | | | | | | | | |
| 19921124 | SCAR CG4636-PA [Drosophila melanogaster] | | | | |  |  |  |  |  |
| 21553113 | WAS protein family, member 3 [Mus musculus] | | | | |  |  |  |  |  |
| 23510313 | WAS protein family, member 2 [Mus musculus] | | | | |  |  |  |  |  |
| 30685943 | WAVE1 (WASP (WISKOTT-ALDRICH SYNDROME PROTEIN)-FAMILY VERPROLIN HOMOLOGOUS PROTEIN 1) [Arabidopsis thaliana] | | | | | | | | | |
| 30687512 | ITB1 (IRREGULAR TRICHOME BRANCH1) [Arabidopsis thaliana] | | | | | |  |  |  |  |
| 41055162 | hypothetical protein LOC394056 [Danio rerio] | | | | |  |  |  |  |  |
| 4507913 | Wiskott-Aldrich syndrome protein family member 1 [Homo sapiens] | | | | | | |  |  |  |
| 48095266 | PREDICTED: similar to SCAR CG4636-PA [Apis mellifera] | | | | | |  |  |  |  |
| 50744594 | PREDICTED: similar to WAS protein family, member 1 [Gallus gallus] | | | | | | |  |  |  |
| 57044069 | PREDICTED: similar to WAS protein family, member 2 [Canis familiaris] | | | | | | |  |  |  |
| 61557159 | WAS protein family, member 2 [Rattus norvegicus] | | | | |  |  |  |  |  |
| 66472704 | hypothetical protein LOC553542 [Danio rerio] | | | | |  |  |  |  |  |
| 66809177 | SCAR1 [Dictyostelium discoideum AX4] | | | |  |  |  |  |  |  |
| 68341973 | WASP family 1 [Rattus norvegicus] | | | |  |  |  |  |  |  |
| 68393243 | PREDICTED: hypothetical protein [Danio rerio] | | | | |  |  |  |  |  |
| 71989455 | WAVE (actin cytoskeleton modulator) homolog family member (wve-1) [Caenorhabditis elegans] | | | | | | | | | |
| 72013000 | PREDICTED: similar to actin binding protein, putative isoform 1 [Strongylocentrotus purpuratus] | | | | | | | | | |
| 73973743 | PREDICTED: similar to Wiskott-Aldrich syndrome protein family member 1 (WASP-family protein member 1) (WAVE-1 protein) (Verprolin homology domain-containing protein 1) isoform 1 [Canis familiaris] | | | | | | | | | |
| 73973745 | PREDICTED: similar to Wiskott-Aldrich syndrome protein family member 1 (WASP-family protein member 1) (WAVE-1 protein) (Verprolin homology domain-containing protein 1) isoform 2 [Canis familiaris] | | | | | | | | | |
| 73973747 | PREDICTED: similar to Wiskott-Aldrich syndrome protein family member 1 (WASP-family protein member 1) (WAVE-1 protein) (Verprolin homology domain-containing protein 1) isoform 3 [Canis familiaris] | | | | | | | | | |
| 73973749 | PREDICTED: similar to Wiskott-Aldrich syndrome protein family member 1 (WASP-family protein member 1) (WAVE-1 protein) (Verprolin homology domain-containing protein 1) isoform 4 [Canis familiaris] | | | | | | | | | |
| 73993386 | PREDICTED: similar to WAS protein family, member 3 isoform 1 [Canis familiaris] | | | | | | | |  |  |
| 73993388 | PREDICTED: similar to WAS protein family, member 3 isoform 3 [Canis familiaris] |  |  |  |  |  |  |  |  |  |
| 73993390 | PREDICTED: similar to WAS protein family, member 3 isoform 4 [Canis familiaris] | | | | | | | |  |  |
| 76631519 | PREDICTED: similar to WAS protein family, member 3 isoform 2 [Bos taurus] | | | | | | | |  |  |
| 79324228 | WAVE1 (WASP (WISKOTT-ALDRICH SYNDROME PROTEIN)-FAMILY VERPROLIN HOMOLOGOUS PROTEIN 1) [Arabidopsis thaliana] | | | | | | | | | |
| 79355351 | WAVE2 (WASP (WISKOTT-ALDRICH SYNDROME PROTEIN)-FAMILY VERPROLIN HOMOLOGOUS PROTEIN 2) [Arabidopsis thaliana] | | | | | | | | | |
| 79476972 | WAVE5 (WASP (WISKOTT-ALDRICH SYNDROME PROTEIN)-FAMILY VERPROLIN HOMOLOGOUS PROTEIN 5) [Arabidopsis thaliana] | | | | | | | | | |
| 79503843 | WAVE3 [Arabidopsis thaliana] | | |  |  |  |  |  |  |  |
| 91087975 | PREDICTED: similar to CG4636-PA [Tribolium castaneum] | | | | | |  |  |  |  |

Rho-family GTPases (overlapping lists):

| **cdc42 - blastres** | | **query: 45384262** | **score** |
| --- | --- | --- | --- |
| Apicomplexa | Babesia_bovis_T2Bo | 156087613 | 77.4 |
| Apicomplexa | Cryptosporidium_hominis_TU502 | 67624139 | 73.9 |
| Apicomplexa | Cryptosporidium_muris_RN66 | 209877729 | 74.3 |
| Apicomplexa | Cryptosporidium_parvum_Iowa_II | 126649339 | 73.9 |
| Apicomplexa | Eimeria_tenella_str-_Houghton | 118490878 | 45.8 |
| Apicomplexa | Plasmodium_berghei_str-_ANKA | 68073783 | 75.1 |
| Apicomplexa | Plasmodium_chabaudi_chabaudi | 70953140 | 72 |
| Apicomplexa | Plasmodium_falciparum_3D7 | 124803934 | 75.1 |
| Apicomplexa | Plasmodium_vivax_SaI-1 | 156095566 | 75.9 |
| Apicomplexa | Plasmodium_yoelii_yoelii_str-_17XNL | 82540395 | 74.7 |
| Apicomplexa | Theileria_annulata_strain_Ankara | 85001490 | 70.1 |
| Apicomplexa | Theileria_parva_strain_Muguga | 71033513 | 70.1 |
| Archamoebae | Entamoeba_dispar_SAW760 | 167394521 | 282 |
| Archamoebae | Entamoeba_histolytica_HM-1-IMSS | 67474492 | 273 |
| Chlorophyta | Chlamydomonas_reinhardtii | 159490221 | 73.9 |
| Chlorophyta | Ostreococcus_lucimarinus_CCE9901 | 145347612 | 162 |
| Choanoflagellida | Monosiga_brevicollis_MX1 | 167527023 | 350 |
| Ciliophora | Paramecium_tetraurelia_strain_d4-2 | 145519884 | 189 |
| Ciliophora | Tetrahymena_thermophila_SB210 | 118365411 | 223 |
| Diplomonadida | Giardia_lamblia_ATCC_50803 | 159111077 | 214 |
| Fungi | Ajellomyces_capsulatus_NAm1 | 154286684 | 269 |
| Fungi | Ashbya_gossypii_ATCC_10895 | 45201003 | 324 |
| Fungi | Aspergillus_clavatus_NRRL_1 | 121715298 | 311 |
| Fungi | Aspergillus_fumigatus_Af293 | 70989707 | 315 |
| Fungi | Aspergillus_nidulans_FGSC_A4 | 67901000 | 317 |
| Fungi | Aspergillus_niger_CBS_513-88 | 145234360 | 315 |
| Fungi | Aspergillus_oryzae_RIB40 | 169769430 | 312 |
| Fungi | Aspergillus_terreus_NIH2624 | 115400705 | 313 |
| Fungi | Botryotinia_fuckeliana_B05-10 | 154290186 | 316 |
| Fungi | Candida_albicans_SC5314 | 68488447 | 315 |
| Fungi | Candida_glabrata_CBS_138 | 50287543 | 324 |
| Fungi | Chaetomium_globosum_CBS_148-51 | 116191977 | 288 |
| Fungi | Coccidioides_immitis_RS | 119192818 | 313 |
| Fungi | Coprinopsis_cinerea_okayama7-130 | 169854193 | 330 |
| Fungi | Cryptococcus_neoformans_var-_neoformans_B-3501A | 134118147 | 273 |
| Fungi | Cryptococcus_neoformans_var-_neoformans_JEC21 | 58270104 | 325 |
| Fungi | Debaryomyces_hansenii_CBS767 | 50427097 | 312 |
| Fungi | Encephalitozoon_cuniculi_GB-M1 | 19074644 | 152 |
| Fungi | Enterocytozoon_bieneusi_H348 | 169806162 | 68.6 |
| Fungi | Gibberella_zeae_PH-1 | 46122139 | 319 |
| Fungi | Kluyveromyces_lactis_NRRL_Y-1140 | 50302503 | 328 |
| Fungi | Laccaria_bicolor_S238N-H82 | 170093940 | 330 |
| Fungi | Lodderomyces_elongisporus_NRRL_YB-4239 | 149247448 | 314 |
| Fungi | Magnaporthe_grisea_70-15 | 39974775 | 317 |
| Fungi | Malassezia_globosa_CBS_7966 | 164663303 | 327 |
| Fungi | Neosartorya_fischeri_NRRL_181 | 119468002 | 214 |
| Fungi | Neurospora_crassa_OR74A | 164425062 | 313 |
| Fungi | Penicillium_marneffei_ATCC_18224 | 212532883 | 310 |
| Fungi | Phaeosphaeria_nodorum_SN15 | 169619585 | 304 |
| Fungi | Pichia_guilliermondii_ATCC_6260 | 146415394 | 312 |
| Fungi | Pichia_stipitis_CBS_6054 | 126132340 | 313 |
| Fungi | Podospora_anserina_DSM_980 | 171686826 | 319 |
| Fungi | Pyrenophora_tritici-repentis_Pt-1C-BFP | 189202740 | 269 |
| Fungi | Saccharomyces_cerevisiae | 6323259 | 323 |
| Fungi | Schizosaccharomyces_japonicus_yFS275 | 213409668 | 321 |
| Fungi | Schizosaccharomyces_pombe | 19114448 | 325 |
| Fungi | Sclerotinia_sclerotiorum_1980 | 156033301 | 314 |
| Fungi | Ustilago_maydis_521 | 71003552 | 330 |
| Fungi | Vanderwaltozyma_polyspora_DSM_70294 | 156842251 | 326 |
| Fungi | Yarrowia_lipolytica_CLIB122 | 210075311 | 320 |
| Metazoa | Acyrthosiphon_pisum | 187113154 | 365 |
| Metazoa | Aedes_aegypti | 157119170 | 365 |
| Metazoa | Anopheles_gambiae_str-_PEST | 158290974 | 365 |
| Metazoa | Apis_mellifera | 66509892 | 368 |
| Metazoa | Bombyx_mori | 112983416 | 76.6 |
| Metazoa | Bos_taurus | 28461213 | 276 |
| Metazoa | Brugia_malayi | 170596121 | 356 |
| Metazoa | Caenorhabditis_briggsae_AF16 | 157750612 | 354 |
| Metazoa | Caenorhabditis_elegans | 17532607 | 353 |
| Metazoa | Canis_lupus_familiaris | 73952550 | 389 |
| Metazoa | Ciona_intestinalis | 74095867 | 364 |
| Metazoa | Culex_quinquefasciatus | 170042943 | 224 |
| Metazoa | Danio_rerio | 41055439 | 389 |
| Metazoa | Drosophila_ananassae | 194762716 | 367 |
| Metazoa | Drosophila_erecta | 194907289 | 256 |
| Metazoa | Drosophila_grimshawi | 195040617 | 366 |
| Metazoa | Drosophila_melanogaster | 17647249 | 365 |
| Metazoa | Drosophila_mojavensis | 195134318 | 366 |
| Metazoa | Drosophila_persimilis | 195162987 | 274 |
| Metazoa | Drosophila_pseudoobscura_pseudoobscura | 125981127 | 365 |
| Metazoa | Drosophila_sechellia | 195345807 | 299 |
| Metazoa | Drosophila_simulans | 195574382 | 236 |
| Metazoa | Drosophila_virilis | 195399209 | 366 |
| Metazoa | Drosophila_willistoni | 195456826 | 367 |
| Metazoa | Drosophila_yakuba | 195499435 | 195 |
| Metazoa | Equus_caballus | 194207324 | 384 |
| Metazoa | Gallus_gallus | 45384262 | 392 |
| Metazoa | Homo_sapiens | 4757952 | 391 |
| Metazoa | Macaca_mulatta | 109070989 | 388 |
| Metazoa | Monodelphis_domestica | 126341942 | 347 |
| Metazoa | Mus_musculus | 6679601 | 280 |
| Metazoa | Nasonia_vitripennis | 156541379 | 369 |
| Metazoa | Nematostella_vectensis | 156373875 | 369 |
| Metazoa | Oncorhynchus_mykiss | 185132101 | 74.7 |
| Metazoa | Ornithorhynchus_anatinus | 149599894 | 341 |
| Metazoa | Oryctolagus_cuniculus | 126722751 | 71.2 |
| Metazoa | Oryzias_latipes | 157278227 | 70.5 |
| Metazoa | Ovis_aries | 187936996 | 159 |
| Metazoa | Pan_troglodytes | 114624142 | 389 |
| Metazoa | Pongo_abelii | 197097420 | 213 |
| Metazoa | Rattus_norvegicus | 16758286 | 269 |
| Metazoa | Salmo_salar | 213512698 | 244 |
| Metazoa | Strongylocentrotus_purpuratus | 72106099 | 363 |
| Metazoa | Sus_scrofa | 118403806 | 375 |
| Metazoa | Taeniopygia_guttata | 206725460 | 70.5 |
| Metazoa | Tribolium_castaneum | 91083695 | 365 |
| Metazoa | Trichoplax_adhaerens | 196007964 | 331 |
| Metazoa | Xenopus_-Silurana-_tropicalis | 62858789 | 386 |
| Metazoa | Xenopus_laevis | 148223413 | 388 |
| Mycetozoa | Dictyostelium_discoideum_AX4 | 66816373 | 271 |
| Streptophyta | Arabidopsis_thaliana | 15236247 | 212 |
| Streptophyta | Oryza_sativa_Japonica_Group | 115445747 | 217 |
| Streptophyta | Physcomitrella_patens_subsp-_patens | 168053999 | 210 |
| Streptophyta | Zea_mays | 162464413 | 210 |
| Trichomonada | Trichomonas_vaginalis_G3 | 154413034 | 217 |

| **RhoA - blastres** | | **query: 2702398** | **score** |
| --- | --- | --- | --- |
| Apicomplexa | Babesia_bovis_T2Bo | 156088917 | 96.7 |
| Apicomplexa | Cryptosporidium_hominis_TU502 | 67610128 | 85.1 |
| Apicomplexa | Cryptosporidium_muris_RN66 | 209880089 | 88.2 |
| Apicomplexa | Cryptosporidium_parvum_Iowa_II | 126643983 | 88.2 |
| Apicomplexa | Eimeria_tenella_str-_Houghton | 118490878 | 50.1 |
| Apicomplexa | Plasmodium_berghei_str-_ANKA | 68068036 | 82.4 |
| Apicomplexa | Plasmodium_chabaudi_chabaudi | 70953492 | 73.6 |
| Apicomplexa | Plasmodium_falciparum_3D7 | 124506171 | 91.3 |
| Apicomplexa | Plasmodium_vivax_SaI-1 | 156095590 | 90.5 |
| Apicomplexa | Plasmodium_yoelii_yoelii_str-_17XNL | 82595062 | 82.4 |
| Apicomplexa | Theileria_annulata_strain_Ankara | 85001490 | 81.3 |
| Apicomplexa | Theileria_parva_strain_Muguga | 71033513 | 80.1 |
| Archamoebae | Entamoeba_dispar_SAW760 | 167387720 | 206 |
| Archamoebae | Entamoeba_histolytica_HM-1-IMSS | 67471776 | 206 |
| Chlorophyta | Chlamydomonas_reinhardtii | 159490221 | 89.4 |
| Chlorophyta | Ostreococcus_lucimarinus_CCE9901 | 145347612 | 139 |
| Choanoflagellida | Monosiga_brevicollis_MX1 | 167521293 | 277 |
| Ciliophora | Paramecium_tetraurelia_strain_d4-2 | 145510196 | 173 |
| Ciliophora | Tetrahymena_thermophila_SB210 | 118365411 | 169 |
| Diplomonadida | Giardia_lamblia_ATCC_50803 | 159111077 | 194 |
| Fungi | Ajellomyces_capsulatus_NAm1 | 154278559 | 274 |
| Fungi | Ashbya_gossypii_ATCC_10895 | 45185413 | 256 |
| Fungi | Aspergillus_clavatus_NRRL_1 | 121709978 | 272 |
| Fungi | Aspergillus_fumigatus_Af293 | 70991469 | 265 |
| Fungi | Aspergillus_nidulans_FGSC_A4 | 67539140 | 271 |
| Fungi | Aspergillus_niger_CBS_513-88 | 145255691 | 268 |
| Fungi | Aspergillus_oryzae_RIB40 | 169769677 | 261 |
| Fungi | Aspergillus_terreus_NIH2624 | 115389176 | 273 |
| Fungi | Botryotinia_fuckeliana_B05-10 | 154315828 | 263 |
| Fungi | Candida_albicans_SC5314 | 68480453 | 268 |
| Fungi | Candida_glabrata_CBS_138 | 50290369 | 263 |
| Fungi | Chaetomium_globosum_CBS_148-51 | 116205009 | 264 |
| Fungi | Coccidioides_immitis_RS | 119187973 | 271 |
| Fungi | Coprinopsis_cinerea_okayama7-130 | 169863550 | 238 |
| Fungi | Cryptococcus_neoformans_var-_neoformans_B-3501A | 134113969 | 266 |
| Fungi | Cryptococcus_neoformans_var-_neoformans_JEC21 | 58269724 | 267 |
| Fungi | Debaryomyces_hansenii_CBS767 | 50415172 | 262 |
| Fungi | Encephalitozoon_cuniculi_GB-M1 | 19074644 | 171 |
| Fungi | Enterocytozoon_bieneusi_H348 | 169806162 | 67.4 |
| Fungi | Gibberella_zeae_PH-1 | 46117116 | 271 |
| Fungi | Kluyveromyces_lactis_NRRL_Y-1140 | 50304105 | 256 |
| Fungi | Laccaria_bicolor_S238N-H82 | 170115635 | 268 |
| Fungi | Lodderomyces_elongisporus_NRRL_YB-4239 | 149246047 | 263 |
| Fungi | Magnaporthe_grisea_70-15 | 39971721 | 275 |
| Fungi | Malassezia_globosa_CBS_7966 | 164656318 | 270 |
| Fungi | Neosartorya_fischeri_NRRL_181 | 119468002 | 271 |
| Fungi | Neurospora_crassa_OR74A | 164422603 | 270 |
| Fungi | Penicillium_marneffei_ATCC_18224 | 212528366 | 265 |
| Fungi | Phaeosphaeria_nodorum_SN15 | 169603658 | 220 |
| Fungi | Pichia_guilliermondii_ATCC_6260 | 146420218 | 253 |
| Fungi | Pichia_stipitis_CBS_6054 | 126275220 | 261 |
| Fungi | Podospora_anserina_DSM_980 | 171689136 | 266 |
| Fungi | Pyrenophora_tritici-repentis_Pt-1C-BFP | 189196188 | 272 |
| Fungi | Saccharomyces_cerevisiae | 6325423 | 269 |
| Fungi | Schizosaccharomyces_japonicus_yFS275 | 213409968 | 270 |
| Fungi | Schizosaccharomyces_pombe | 19115402 | 266 |
| Fungi | Sclerotinia_sclerotiorum_1980 | 156059708 | 263 |
| Fungi | Ustilago_maydis_521 | 71023303 | 266 |
| Fungi | Vanderwaltozyma_polyspora_DSM_70294 | 156839698 | 270 |
| Fungi | Yarrowia_lipolytica_CLIB122 | 50549595 | 265 |
| Metazoa | Acyrthosiphon_pisum | 193688251 | 329 |
| Metazoa | Aedes_aegypti | 157134623 | 331 |
| Metazoa | Anopheles_gambiae_str-_PEST | 31210169 | 331 |
| Metazoa | Apis_mellifera | 66546653 | 329 |
| Metazoa | Bombyx_mori | 112983246 | 88.2 |
| Metazoa | Bos_taurus | 194680280 | 214 |
| Metazoa | Brugia_malayi | 170586398 | 327 |
| Metazoa | Caenorhabditis_briggsae_AF16 | 157754417 | 235 |
| Metazoa | Caenorhabditis_elegans | 17541992 | 325 |
| Metazoa | Canis_lupus_familiaris | 54792727 | 362 |
| Metazoa | Ciona_intestinalis | 74096011 | 320 |
| Metazoa | Culex_quinquefasciatus | 170030845 | 182 |
| Metazoa | Danio_rerio | 56118288 | 358 |
| Metazoa | Drosophila_ananassae | 194745688 | 217 |
| Metazoa | Drosophila_erecta | 194907289 | 217 |
| Metazoa | Drosophila_grimshawi | 195029683 | 330 |
| Metazoa | Drosophila_melanogaster | 17137100 | 328 |
| Metazoa | Drosophila_mojavensis | 195121790 | 272 |
| Metazoa | Drosophila_persimilis | 195171178 | 330 |
| Metazoa | Drosophila_pseudoobscura_pseudoobscura | 125773267 | 220 |
| Metazoa | Drosophila_sechellia | 195330404 | 178 |
| Metazoa | Drosophila_simulans | 195574382 | 205 |
| Metazoa | Drosophila_virilis | 195383972 | 244 |
| Metazoa | Drosophila_willistoni | 195444483 | 219 |
| Metazoa | Drosophila_yakuba | 195499435 | 179 |
| Metazoa | Equus_caballus | 194210980 | 311 |
| Metazoa | Gallus_gallus | 45382667 | 364 |
| Metazoa | Homo_sapiens | 10835049 | 364 |
| Metazoa | Macaca_mulatta | 109039680 | 279 |
| Metazoa | Monodelphis_domestica | 126331373 | 354 |
| Metazoa | Mus_musculus | 51709421 | 357 |
| Metazoa | Nasonia_vitripennis | 156544950 | 330 |
| Metazoa | Nematostella_vectensis | 156379567 | 330 |
| Metazoa | Oncorhynchus_mykiss | 185132101 | 67.8 |
| Metazoa | Ornithorhynchus_anatinus | 149534968 | 353 |
| Metazoa | Oryctolagus_cuniculus | 156119376 | 92 |
| Metazoa | Oryzias_latipes | 157278227 | 69.3 |
| Metazoa | Ovis_aries | 187936996 | 151 |
| Metazoa | Pan_troglodytes | 114586866 | 266 |
| Metazoa | Pongo_abelii | 197097420 | 365 |
| Metazoa | Rattus_norvegicus | 16923986 | 364 |
| Metazoa | Salmo_salar | 213511582 | 316 |
| Metazoa | Strongylocentrotus_purpuratus | 115942946 | 317 |
| Metazoa | Sus_scrofa | 178056616 | 214 |
| Metazoa | Taeniopygia_guttata | 206725460 | 88.2 |
| Metazoa | Tribolium_castaneum | 91086361 | 325 |
| Metazoa | Trichoplax_adhaerens | 195996687 | 334 |
| Metazoa | Xenopus_-Silurana-_tropicalis | 62857977 | 351 |
| Metazoa | Xenopus_laevis | 148225697 | 359 |
| Mycetozoa | Dictyostelium_discoideum_AX4 | 66814792 | 210 |
| Spirochaetes_-class- | Leptospira_biflexa_serovar_Patoc_strain_-Patoc_1_-Paris- | 183220912 | 44.7 |
| Streptophyta | Arabidopsis_thaliana | 15236247 | 189 |
| Streptophyta | Oryza_sativa_Japonica_Group | 115445747 | 187 |
| Streptophyta | Physcomitrella_patens_subsp-_patens | 168018751 | 187 |
| Streptophyta | Zea_mays | 162464339 | 186 |
| Trichomonada | Trichomonas_vaginalis_G3 | 123402109 | 183 |

| **Rac** | **query: 2225894** | | **score** |
| --- | --- | --- | --- |
| Archamoebae | Entamoeba_dispar_SAW760 | 167394521 | 137 |
| Archamoebae | Entamoeba_histolytica_HM-1-IMSS | 67469707 | 140 |
| Chlorophyta | Ostreococcus_lucimarinus_CCE9901 | 145347612 | 103 |
| Choanoflagellida | Monosiga_brevicollis_MX1 | 167537282 | 147 |
| Ciliophora | Paramecium_tetraurelia_strain_d4-2 | 145525152 | 119 |
| Ciliophora | Tetrahymena_thermophila_SB210 | 118365411 | 110 |
| Diplomonadida | Giardia_lamblia_ATCC_50803 | 159111077 | 113 |
| Fungi | Ajellomyces_capsulatus_NAm1 | 154286684 | 139 |
| Fungi | Ashbya_gossypii_ATCC_10895 | 45201003 | 118 |
| Fungi | Aspergillus_clavatus_NRRL_1 | 121704772 | 139 |
| Fungi | Aspergillus_fumigatus_Af293 | 71000586 | 139 |
| Fungi | Aspergillus_nidulans_FGSC_A4 | 67537146 | 138 |
| Fungi | Aspergillus_niger_CBS_513-88 | 145245389 | 127 |
| Fungi | Aspergillus_oryzae_RIB40 | 169780050 | 189 |
| Fungi | Aspergillus_terreus_NIH2624 | 115402615 | 138 |
| Fungi | Botryotinia_fuckeliana_B05-10 | 154290186 | 117 |
| Fungi | Candida_albicans_SC5314 | 68488447 | 118 |
| Fungi | Candida_glabrata_CBS_138 | 50287543 | 118 |
| Fungi | Chaetomium_globosum_CBS_148-51 | 116193453 | 136 |
| Fungi | Coccidioides_immitis_RS | 119192090 | 138 |
| Fungi | Coprinopsis_cinerea_okayama7-130 | 169854193 | 119 |
| Fungi | Cryptococcus_neoformans_var-_neoformans_B-3501A | 134118147 | 140 |
| Fungi | Cryptococcus_neoformans_var-_neoformans_JEC21 | 58261962 | 140 |
| Fungi | Debaryomyces_hansenii_CBS767 | 50423877 | 119 |
| Fungi | Gibberella_zeae_PH-1 | 46129344 | 138 |
| Fungi | Kluyveromyces_lactis_NRRL_Y-1140 | 50302503 | 117 |
| Fungi | Laccaria_bicolor_S238N-H82 | 170085235 | 141 |
| Fungi | Lodderomyces_elongisporus_NRRL_YB-4239 | 149247448 | 118 |
| Fungi | Magnaporthe_grisea_70-15 | 39970529 | 138 |
| Fungi | Malassezia_globosa_CBS_7966 | 164659348 | 120 |
| Fungi | Neosartorya_fischeri_NRRL_181 | 119468002 | 100 |
| Fungi | Neurospora_crassa_OR74A | 164429742 | 127 |
| Fungi | Penicillium_marneffei_ATCC_18224 | 212544762 | 139 |
| Fungi | Phaeosphaeria_nodorum_SN15 | 169595186 | 140 |
| Fungi | Pichia_guilliermondii_ATCC_6260 | 146415394 | 118 |
| Fungi | Pichia_stipitis_CBS_6054 | 126132340 | 118 |
| Fungi | Podospora_anserina_DSM_980 | 171684857 | 137 |
| Fungi | Pyrenophora_tritici-repentis_Pt-1C-BFP | 189202740 | 140 |
| Fungi | Saccharomyces_cerevisiae | 6323259 | 118 |
| Fungi | Schizosaccharomyces_japonicus_yFS275 | 213409668 | 117 |
| Fungi | Schizosaccharomyces_pombe | 19114448 | 117 |
| Fungi | Sclerotinia_sclerotiorum_1980 | 156033301 | 116 |
| Fungi | Ustilago_maydis_521 | 71004510 | 143 |
| Fungi | Vanderwaltozyma_polyspora_DSM_70294 | 156842251 | 118 |
| Fungi | Yarrowia_lipolytica_CLIB122 | 50553983 | 142 |
| Metazoa | Acyrthosiphon_pisum | 193625043 | 146 |
| Metazoa | Aedes_aegypti | 157124049 | 147 |
| Metazoa | Anopheles_gambiae_str-_PEST | 158294192 | 146 |
| Metazoa | Apis_mellifera | 66558220 | 147 |
| Metazoa | Bos_taurus | 28461213 | 144 |
| Metazoa | Brugia_malayi | 170571252 | 147 |
| Metazoa | Caenorhabditis_briggsae_AF16 | 157755161 | 264 |
| Metazoa | Caenorhabditis_elegans | 71982576 | 268 |
| Metazoa | Canis_lupus_familiaris | 73969756 | 144 |
| Metazoa | Ciona_intestinalis | 74096209 | 148 |
| Metazoa | Culex_quinquefasciatus | 170030845 | 114 |
| Metazoa | Danio_rerio | 189515207 | 149 |
| Metazoa | Drosophila_ananassae | 194748537 | 145 |
| Metazoa | Drosophila_erecta | 194903164 | 119 |
| Metazoa | Drosophila_grimshawi | 195017984 | 145 |
| Metazoa | Drosophila_melanogaster | 17136856 | 145 |
| Metazoa | Drosophila_mojavensis | 195125661 | 145 |
| Metazoa | Drosophila_persimilis | 195162987 | 145 |
| Metazoa | Drosophila_pseudoobscura_pseudoobscura | 125981127 | 124 |
| Metazoa | Drosophila_sechellia | 195345807 | 124 |
| Metazoa | Drosophila_simulans | 195572272 | 118 |
| Metazoa | Drosophila_virilis | 195375742 | 145 |
| Metazoa | Drosophila_willistoni | 195428178 | 145 |
| Metazoa | Drosophila_yakuba | 195499435 | 120 |
| Metazoa | Equus_caballus | 149743028 | 144 |
| Metazoa | Gallus_gallus | 45384328 | 145 |
| Metazoa | Homo_sapiens | 9845509 | 148 |
| Metazoa | Macaca_mulatta | 109107716 | 128 |
| Metazoa | Monodelphis_domestica | 126339685 | 216 |
| Metazoa | Mus_musculus | 6679601 | 144 |
| Metazoa | Nasonia_vitripennis | 156549294 | 125 |
| Metazoa | Nematostella_vectensis | 156366819 | 148 |
| Metazoa | Ornithorhynchus_anatinus | 149430121 | 144 |
| Metazoa | Pan_troglodytes | 114554566 | 168 |
| Metazoa | Rattus_norvegicus | 16758286 | 104 |
| Metazoa | Salmo_salar | 213512698 | 129 |
| Metazoa | Strongylocentrotus_purpuratus | 72124984 | 147 |
| Metazoa | Sus_scrofa | 178056616 | 128 |
| Metazoa | Tribolium_castaneum | 91092998 | 147 |
| Metazoa | Trichoplax_adhaerens | 196000442 | 144 |
| Metazoa | Xenopus_-Silurana-_tropicalis | 58332274 | 145 |
| Metazoa | Xenopus_laevis | 147906150 | 146 |
| Mycetozoa | Dictyostelium_discoideum_AX4 | 111219440 | 147 |
| Streptophyta | Arabidopsis_thaliana | 15235495 | 142 |
| Streptophyta | Oryza_sativa_Japonica_Group | 115464861 | 139 |
| Streptophyta | Physcomitrella_patens_subsp-_patens | 168018751 | 122 |
| Streptophyta | Zea_mays | 162464413 | 140 |
| Trichomonada | Trichomonas_vaginalis_G3 | 154413034 | 127 |
