# Supplementary material for: The origins of phagocytosis and eukaryogenesis
Source: Biol Direct. 2009 Feb 26;4:9. doi: 10.1186/1745-6150-4-9 (PMC2651865; doi:10.1186/1745-6150-4-9)
Supplement: Additional file 5 — Complete figure legends. [file 1745-6150-4-9-S5.doc]

**The origins of phagocytosis and eukaryogenesis**

Natalya Yutin, Maxim Y. Wolf, Yuri I. Wolf, Eugene V. Koonin*

**Complete figure legends**

Figure.1 . **A Maximum Likelihood tree of actin-related proteins**. The root position was forced between the HSP70 superfamily and the actin superfamily. The tree was constructed by analysis of 295 aligned amino acid residues (Additional File 5). Support values are indicated only for major internal branches (not within smaller monophyletic groups). The protein sequences whose structure alignment was used to correct the multiple protein alignment of actin-related proteins are denoted in red. 3DAW_A: alpha-actin, gi 194709198 (*Oryctolagus* *cuniculus*); 2BTF_A: beta-actin, gi 157881403 (*Bos* *taurus*); 1NM1_A: actin, gi 28374073 (*Dictyostelium* *discoideum*); 1K8K_B: actin-related protein 2, gi 17943200 (*Bos* *taurus*); 1K8K_A: actin-related protein 3, gi 17943199 (*Bos* *taurus*); 2ZGY_A: stable plasmid inheritance protein A (StbA), gi 167745018 (*Escherichia* *coli*); 2FSN_A: Ta0583, gi 99032295 (*Thermoplasma* *acidophilum*); 1JCF_A: MreB, gi 15988309 (*Thermotoga* *maritima*); 2E8A_A: Hsp70, gi 166007013 (*Homo* *sapiens*). Species abbreviations:

Ac Calma, *Caldivirga* *maquilingensis* IC-167; Ac Pyrae, *Pyrobaculum* *aerophilum* str. IM2; Ac Pyrar, *Pyrobaculum* *arsenaticum* DSM 13514; Ac Pyrca, *Pyrobaculum* *calidifontis* JCM 11548; Ac Pyris, *Pyrobaculum* *islandicum* DSM 4184; Ac Thene, *Thermoproteus* *neutrophilus* V24Sta; Ac Thepe, *Thermofilum* *pendens* Hrk 5; Ae Arcfu, *Archaeoglobus* *fulgidus* DSM 4304; Ae Methu, *Methanospirillum* *hungatei* JF-1; Ae Metka, *Methanopyrus* *kandleri* AV19; Ae Metsm, *Methanobrevibacter* *smithii* ATCC 35061; Ae Metth, *Methanothermobacter* *thermautotrophicus* str. Delta H; Ae Natph, *Natronomonas* *pharaonis* DSM 2160; Ae Thevo, *Thermoplasma* *volcanium* GSS1; Ak CanKo, *Candidatus* *Korarchaeum* *cryptofilum* OPF8; Ba Colae, *Collinsella* *aerofaciens* ATCC 25986; Bb Algsp, *Algoriphagus* sp. PR1; Bc Proma, *Prochlorococcus* *marinus* str. MIT 9215; Bf Anaca, *Anaerostipes* *caccae* DSM 14662; Bf Bacsp, *Bacillus* sp. SG-1; Bf Calsa, *Caldicellulosiruptor* *saccharolyticus* DSM 8903; Bf Clodi, *Clostridium* *difficile* QCD-37x79; Bf Cloph, *Clostridium* *phytofermentans* ISDg; Bf Mooth, *Moorella* *thermoacetica* ATCC 39073; Bf Rumto, *Ruminococcus* *torques* ATCC 27756; Bf Synwo, *Syntrophomonas* *wolfei* subsp. *wolfei* str. *Goettingen*; Bf Theps, *Thermoanaerobacter* *pseudethanolicus* ATCC 33223; Bf Thete, *Thermoanaerobacter* *tengcongensis* MB4; Bh Chlag, *Chloroflexus* *aggregans* DSM 9485; Bp Acicr, *Acidiphilium* *cryptum* JF-5; Bp Escco, *Escherichia* *coli*; Bp Helpy, *Helicobacter* *pylori* J99; Bp Magma, *Magnetospirillum* *magneticum* AMB-1; Bp Magry, *Magnetospirillum* *gryphiswaldense*; Bp Magsp, *Magnetococcus* sp. MC-1; Bp Serma, *Serratia* *marcescens*; Bp Sphwi, *Sphingomonas* *wittichii* RW1; Bp delpr, delta proteobacterium MLMS-1; Bt Thema, *Thermotoga* *maritima* MSB8; Bt Theme, *Thermosipho* *melanesiensis* BI429; Bv Opite, *Opitutus* *terrae* PB90-1; E7 Cyame, *Cyanidioschyzon* *merolae* strain 10D; E9 Arath, *Arabidopsis* *thaliana*; E9 Orysa, *Oryza* *sativa* (*japonica* *cultivar*-group); Ec Babbo, *Babesia* *bovis* T2Bo; Ec Cryho, *Cryptosporidium* *hominis* TU502; Ec Parte, *Paramecium* *tetraurelia* strain d4-2; Ec Tetth, *Tetrahymena* *thermophila* SB210; Ei Giala, *Giardia* *lamblia* ATCC 50803; Ej Enthi, *Entamoeba* *histolytica* HM-1:IMSS; Ek Leima, *Leishmania* major strain *Friedlin*; Ek Trybr, *Trypanosoma* *brucei* TREU927; Ek Trycr, *Trypanosoma* *cruzi* strain CL Brener; El Anoga, *Anopheles* *gambiae* str. PEST; El Caeel, *Caenorhabditis* *elegans*; El Danre, *Danio* *rerio*; El Drome, *Drosophila* *melanogaster*; El Homsa, *Homo* *sapiens*; El Klula, *Kluyveromyces* *lactis*; El Lacbi, *Laccaria* *bicolor* S238N-H82; El Musmu, *Mus* *musculus*; El Sacce, *Saccharomyces* *cerevisiae*; Ev Dicdi, *Dictyostelium* *discoideum* AX4; Ew Triva, *Trichomonas* *vaginalis* G3.

Figure 2. **Selected prokaryotic actin homologs aligned with eukaryotic actins and actin-related proteins 2 and 3**

Green boxes 1 and 2 highlight the major inserts in Arp 2, 3 distinguishing them from actins [75]: a loop in subdomain 4 of Arp3 (green box 1) and elongated loops in subdomain 3 in both Arp 2 and Arp 3 (green box 2) ALP, actin-like protein. Red boxes indicate homologous inserts shared between crenarchaeal and eukaryotic proteins. MreB_1JCG: 15988310, *Thermotoga* *maritima*; MreB_Pasmu: 15603820, *Pasteurella* *multocida*; MreB_Bacam: 154686938, *Bacillus* *amyloliquefaciens*; MreB_Ricpr: 15604602, *Rickettsia* *prowazekii*; MreB_Metth: 15679042, *Methanothermobacter* *thermautotrophicus*; ParM_2ZGY: 167745019, *Escherichia* *coli*; ParM_Shidy: 82524481, *Shigella* *dysenteriae*; Ta0583_Theac: 74544304, *Thermoplasma* *acidophilum*; ALP_CanKo: 170290893, *Candidatus* *Korarchaeum* *cryptofilum*; ALP_Thepe: 119719444, *Thermofilum* *pendens*; ALP_Calma: 159041446, *Caldivirga* *maquilingensis*; ALP_Pyrar: 145592015, *Pyrobaculum* *arsenaticum*; ALP_Pyrca: 126460240, *Pyrobaculum* *calidifontis*; ALP_Pyrae: 18313231, *Pyrobaculum* *aerophilum*; ALP_Pyris: 119872399, *Pyrobaculum* *islandicum*; ALP_Thene: 171185893, *Thermoproteus* *neutrophilus*; Arp3_Dicdi: 66810313, *Dictyostelium* *discoideum*; Arp3_Sacce: 6322525, *Saccharomyces* *cerevisiae*; Arp3_Caeel: 17510483, *Caenorhabditis* *elegans*; Arp3_1K8K_A: 17943199, *Bos* *taurus*; arp2_Dicdi: 66823841, *Dictyostelium* *discoideum*; Arp2_Sacce: 6320175, *Saccharomyces* *cerevisiae*; arp2_Caeel: 17562290, *Caenorhabditis* *elegans*; Arp2_1K8K_B: 17943200, *Bos* *taurus*; Act_1NM1: 28374073, *Dictyostelium* *discoideum*; Act_Sacce: 38372623, *Saccharomyces* *cerevisiae*; Act_Caeel: 113291, *Caenorhabditis* *elegans*; Act_Bosta: 27819614, *Bos* *taurus*.

Figure 3. **A Maximum Likelihood tree of the Ras superfamily of GTPases**

The tree was constructed using 120 aligned positions (additional File 6). The tree is unrooted but shown in a pseudo-rooted form solely for convenience. Bacterial and archaeal clusters are shown in green and red, respectively. Support values are shown only for major internal branches. Species abbreviations: Ac Censy, *Cenarchaeum* *symbiosum* A; Ac Hypbu, *Hyperthermus* *butylicus* DSM 5456; Ac Pyrae, *Pyrobaculum* *aerophilum* str. IM2; Ac Pyrar, *Pyrobaculum* *arsenaticum* DSM 13514; Ac Pyrca, *Pyrobaculum* *calidifontis* JCM 11548; Ac Sulac, *Sulfolobus* *acidocaldarius* DSM 639; Ac Thepe, *Thermofilum* *pendens* Hrk 5; Ae Arcfu, *Archaeoglobus* *fulgidus* DSM 4304; Ae Halsp, *Halobacterium* sp. NRC-1; Ae Metba, *Methanosarcina* *barkeri* str. Fusaro; Ae Metbu, *Methanococcoides* *burtonii* DSM 6242; Ae Metka, *Methanopyrus* *kandleri* AV19; Ae Metma45359278, *Methanococcus* *maripaludis* S2; Ae Metma126179736, *Methanoculleus* *marisnigri* JR1; Ae Metth15678622, *Methanosaeta* *thermophila* PT; Ae Metth15679885, *Methanothermobacter* *thermautotrophicus* str. Delta H; Ae Theko, *Thermococcus* *kodakarensis* KOD1; Ak CanKo, *Candidatus* *Korarchaeum* *cryptofilum* OPF8; Bb Flaba, *Flavobacteriales* *bacterium* HTCC2170; Bb Micma, *Microscilla* *marina* ATCC 23134; Bb Polsp, *Tenacibaculum* sp. MED152; Bc Acama, *Acaryochloris* *marina* MBIC11017; Bc Cyasp, *Cyanothece* sp.; Bh Herau, *Herpetosiphon* *aurantiacus* ATCC 23779; Bh Rosca, *Roseiflexus* *castenholzii* DSM 13941; Bp Decar, *Dechloromonas* *aromatica* RCB; Bp Desal, *Desulfatibacillum* *alkenivorans* AK-01; Bp Myxxa, *Myxococcus* *xanthus* DK 1622; Bp Pelca, *Pelobacter* *carbinolicus* DSM 2380; Bp Pseat, *Pseudoalteromonas* *atlantica* T6c; Bq Aquae, *Aquifex* *aeolicus* VF5; Bq Hydsp, *Hydrogenivirga* sp. 128-5-R1-1. Eukaryotic Ras sequences were taken from Dong et al [80]. The complete list of the 67 bacterial and archaeal proteins that belong to the Rab-Ran-Ras-Rho branch is given in Additional File 7.

Figure 4. **The proposed endosymbiotic scenario of eukaryogenesis and subsequent origin of phagocytosis**.

The evolutionary tree of archaea is shown as a multifurcation of 5 major branches: Crenarchaeota, Euryarchaeota, Korarchaeota, Thaumarchaeota, and the hypothetical Archaeal Ancestor of Eukaryotes which is depicted as an irregular shape to emphasize the likely absence of a rigid cell wall. LECA, Last Universal Eukaryotic Ancestor. HGT, Horizontal Gene Transfer. The primary radiation of eukaryotes is shown as a multifurcation of 5 supergroups: Unikonts, Chromalveolata, Excavates, Rhizaria, and Planta. At least, three of the supergroups evolved full-fledged phagocytosis (Ph).
