# Supplementary material for: The origins of phagocytosis and eukaryogenesis
Source: Biol Direct. 2009 Feb 26;4:9. doi: 10.1186/1745-6150-4-9 (PMC2651865; doi:10.1186/1745-6150-4-9)
Supplement: Additional file 6 — List of prokaryotic GTPases that cluster with the eukaryotic Rab, Ras, Ran, and Rho families. [file 1745-6150-4-9-S6.doc]

The complete list of the 67 prokaryotic proteins that belonged to the Rab-Ran-Ras-Rho branch:

**Crenarchaeota**: *Hyperthermus butylicus* DSM 5456 (124027211), *Thermofilum pendens* Hrk 5 (119718998); Euryarchaeota: *Methanosarcina acetivorans* C2A (20091139), *Methanosarcina barkeri* str. Fusaro (73669795, 73669930, 73670787);

**Chlorobi**: *Chlorobium chlorochromatii* CaD3 (78188486), *Chlorobium tepidum* TLS (21674346);

**Bacteroidetes**: *Croceibacter atlanticus* HTCC2559 (83855878), *Dokdonia donghaensis* MED134 (86130898), *Flavobacteriales* bacterium HTCC2170 (88711358), *Flavobacteria* bacterium BBFL7 (89890012), *Kordia algicida* OT-1 (163753493), *Microscilla marina* ATCC 23134 (124002318, 124002660, 124003178, 124004058, 124005782, 124009437, 124009795), *Polaribacter* sp. MED152 (86133741)*, Porphyromonas gingivalis* ATCC 33277 (188995660), *Porphyromonas gingivalis* W83 (34541475), unidentified eubacterium SCB49 (149369553);

**Cyanobacteria**: *Acaryochloris marina* MBIC11017 (158335132, 158335320, 158336136, 158337861, 158339421*), Anabaena variabilis* ATCC 29413 (75907104, 75907715), *Crocosphaera watsonii* WH 8501 (67923492, 67924809), *Cyanothece* sp. CCY 0110 (126656423, 126659427, 126659720), *Cyanothece* sp. ATCC 51142 (172039621), *Cyanothece* sp. PCC 8801 (177663635, 177665067), *Cyanothece* sp. PCC 7424 (186898775, 186899363), *Lyngbya* sp. PCC 8106 (119488987), *Nostoc punctiforme* PCC 73102 (186681684, 186682295, 186686076, 186686533), *Nostoc* sp. PCC 7120 (17227620, 17230522), *Trichodesmium erythraeum* IMS101 (113475136, 113477255);

**Chloroflexi**: *Chloroflexus aggregans* DSM 9485 (118045180), *Chloroflexus aurantiacus* J-10-fl (163846810), *Roseiflexus castenholzii* DSM 13941 (156743541);

**Planctomycetes**: *Gemmata obscuriglobus* UQM 2246 (168704801);

**Proteobacteria**: *Beggiatoa* sp. PS (153872025, 153873994, 153874425), *Desulfatibacillum alkenivorans* AK-01 (163722879), *Magnetococcus* sp. MC-1 (117923775), *Mesorhizobium* *loti* MAFF303099 (13472827), *Pseudoalteromonas* *atlantica* T6c (109900290), *Rhodospirillum* *rubrum* ATCC 11170 (83592173), *Roseobacter* sp. CCS2 (126733913), *Sagittula* *stellata* E-37 (126729792), *Sorangium* *cellulosum* 'So ce 56' (162454092), *Sulfurovum* sp. NBC37-1 (152992431);

**Chlamydiae/Verrucomicrobia group**: *Verrucomicrobium* *spinosum* DSM 4136 (171915047).
